# Development of a Global Metabo-Lipid-Prote-omics Workflow to Compare Healthy Proximal and Distal Colonic Epithelium in Mice

**DOI:** 10.1021/acs.jproteome.3c00771

**Published:** 2024-07-25

**Authors:** Maryam Hemmati, Susanne I. Wudy, Franziska Hackbarth, Verena K. Mittermeier-Kleßinger, Olivia I. Coleman, Dirk Haller, Christina Ludwig, Corinna Dawid, Karin Kleigrewe

**Affiliations:** † Bavarian Center for Biomolecular Mass Spectrometry, TUM School of Life Sciences, Technical University of Munich, 85354 Freising, Germany; ‡ Chair of Food Chemistry and Molecular Sensory Science, TUM School of Life Sciences, Technical University of Munich, 85354 Freising, Germany; § Professorship for Functional Phytometabolomics, TUM School of Life Sciences, Technical University of Munich, 85354 Freising, Germany; ∥ Chair of Nutrition and Immunology, TUM School of Life Sciences, Technical University of Munich, 85354 Freising, Germany; ⊥ ZIEL Institute for Food and Health, Technical University of Munich, 85354 Freising, Germany

**Keywords:** metabo-lipid-proteomics, methyl *tert*-butyl ether, mouse proximal and distal colons, multiomics

## Abstract

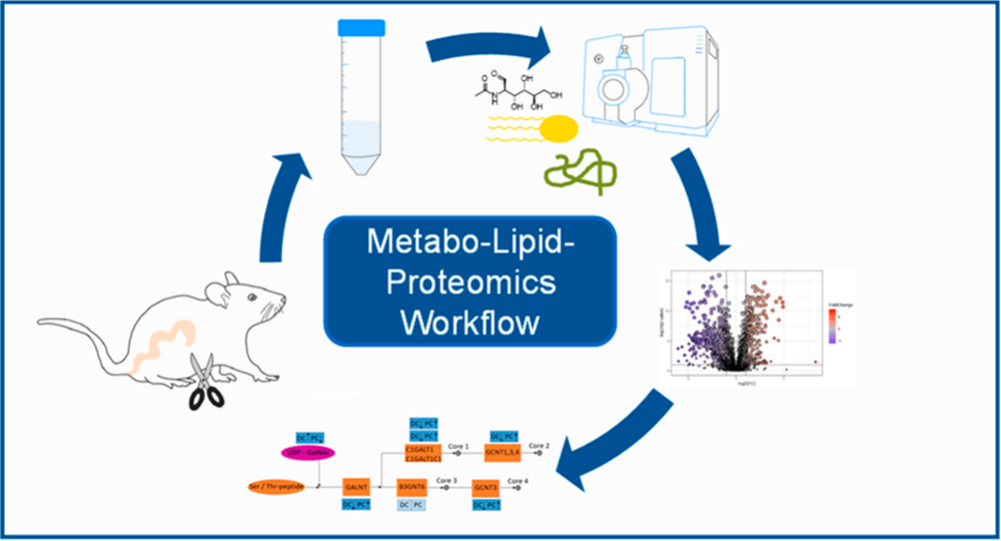

A multimetabo-lipid-prote-omics workflow was developed to characterize the molecular interplay within proximal (PC) and distal (DC) colonic epithelium of healthy mice. This multiomics data set lays the foundation to better understand the two tissue types and can be used to study, for example, colon-related diseases like colorectal cancer or inflammatory bowel disease. First, the methyl *tert*-butyl ether extraction method was optimized, so that from a single tissue biopsy >350 reference-matched metabolites, >1850 reference-matched lipids, and >4500 proteins were detected by using targeted and untargeted metabolomics, untargeted lipidomics, and proteomics. Next, each omics-data set was analyzed individually and then merged with the additional omics disciplines to generate a deep understanding of the underlying complex regulatory network within the colon. Our data demonstrates, for example, differences in mucin formation, detected on substrate level as well as on enzyme level, and altered lipid metabolism by the detection of phospholipases hydrolyzing sphingomyelins to ceramides. In conclusion, the combination of the three mass spectrometry-based omics techniques can better entangle the functional and regional differences between PC and DC tissue compared to each single omics technique.

## Introduction

For a better understanding of molecular mechanisms involved in complex biological systems, the interplay of endogenous bioactive lipids, metabolites, and proteins requires an in-depth interpretation. However, integration of different “omics” from a single biopsy involves major challenges.^1^ For an efficient multiomics approach, the following steps need to be evaluated and optimized: (1) sample preparation, (2) chromatographic and mass spectrometric measurements, (3) processing and analysis of each single omics data set, and (4) comprehensive multiomics-data integration with biological interpretation.

First, optimal multiomics sample preparation demands to fully isolate lipids, metabolites, and proteins from one single sample. Historically, the extraction protocol based on chloroform/methanol has proven to be robust and applicable for lipidomics,^2,3^ and it has been recently adapted to multiomics applications of diverse sets of human and animal biopsies and bacteria.^4,5^ Nevertheless, chloroform is not eco-friendly due to its toxicity and also has a low ability for the extraction of polar compounds.^6^ Alternatively, methyl *tert*-butyl ether (MTBE) extraction methods based on the original publication from Matyash et al.^7^ using MTBE, methanol, and water are not only safer (less carcinogenic/toxic) but also provide a good quantitative recovery of more polar lipids and metabolites.^8−11^ This biphasic organic extraction method has gained popularity in terms of the extraction efficiency, practicality, and coverage for the metabo-lipid-omics extraction from human plasma, human plasma and urine,^10^ mouse brain tissue,^12^ mouse liver and muscle tissues,^8,11^ and for the lipid-prote-omics extraction from human malignant plasma cells.^13,14^ Additionally, the MTBE extraction method exhibits high efficiency in the simultaneous extraction of metabolites, lipids, and proteins from mesenchymal cells of mouse bone marrow,^11,15^ hippocampal cells of rat brain,^14^ different tissues of mouse,^15^ and *Helicobacter pylori* bacteria.^16^ In these cases, the polar metabolites accumulate in the lower methanol/water phase, and hydrophobic metabolites as well as the lipids are transferred into the upper MTBE-phase and the proteins can be extracted from the remaining pellet. However, to reach the best comprehensive coverage of metabolites, lipids, and proteins from a single colon biopsy, optimization of the solvents and their ratios are necessary and need to be evaluated and validated for each omics-set.

Second, for optimal mass spectrometric measurements the applied analytical methods to cover the metabolome, lipidome, or proteome needs to be adapted and validated for each sample type. Targeted mass spectrometry (MS) is widely used to quantify smaller sets of prespecified metabolites, lipids, or proteins by applying spiked-in stable-isotope (SIL-IS) references at the beginning of the sample preparation. Within this manuscript, the classical multiple reaction monitoring (MRM) methods are used to quantify amino acids,^17^ acylcarnitines,^17^ short-chain fatty acids (SCFA),^18^ organic acids,^19,20^ nucleotides and nucleosides,^21^ and free fatty acids (FFA).^22^ These sets of metabolites give insights into amino acids availability for protein biosynthesis, energy supply via acylcarnitines, nucleotides, nucleosides, FFAs, and the gut-microbiome derived SCFAs and also insight into primary metabolism via the organic acids. In addition, untargeted mass spectrometry by combination of hydrophilic interaction chromatography (HILIC) and reversed-phase (RP) liquid chromatography (LC) coupled to high-resolution mass spectrometers (HR-MS) using data-dependent acquisition (DDA) are used to comprehensively identify and relatively quantify a various range of metabolites, lipids, and proteins with different polarities.^23−28^

Third, metabolomics and lipidomics mass spectrometric data is analyzed using common tools like vendor specific software, MS-Dial,^29^ MZmine,^30,31^ XCMS,^32,33^ or MetaboAnalyst.^34^ Recently, a lipidome atlas was added to the MS-Dial portfolio generating a useful tool for lipidomics applications.^35,36^ The main challenge for untargeted mass spectrometry in the field of metabolomics and lipidomics is the correct annotation of the detected mass spectrometric features. Next to publically available databases, tools like GNPS,^37^ Sirius,^38^ and MetFrag^39^ help to get a better annotation of the identified compounds. For untargeted proteomics, MaxQuant is one of the most commonly used tools in the field.^40^ After proper data processing and normalization, the next steps are statistical analysis using parametric or nonparametric testing, principal component analysis (PCA), and the functional analysis of the obtained results.^41,42^

The major benefit of a multiomics approach is that all single omics data can be combined in the fourth step. Here, novel bioinformatics tools like BioPAN^43^ can be used to find novel biological insights by discovering lipid pathways and related genes or joint pathways between metabolites, lipids, and proteins by means of joint-pathway analysis using the MetaboAnalyst platform, or Pathview.^34,44^

In this publication, the newly developed multiomics method was used to study the mechanistic interplay of metabolites, lipids, and proteins in mouse colon tissue. In general, the proximal colon tissue (PC tissue) is responsible for the absorption of water and electrolytes from the undigested food and plays a role in the formation of feces. The distal colon tissue (DC tissue) removes additional water and stores and compacts the fecal material before defecation.^45^ The PC tissue (cecum, ascending, and initial two-thirds of transverse) developed from the midgut, whereas the DC tissue (terminal one-third of transverse, descending, and sigmoid) from the hindgut.^46^ Additionally, the colon has a segmental cell heterogeneity with an impact on immune cell response.^47^ Another difference is that the blood supply to the PC tissue depends on the superior mesenteric artery, whereas the distal part of the transverse colon onward is supplied by the inferior mesenteric artery, which is in line with differences in vascularization and lymphatic drainage.^48^ Also, differences in gene expression in the proximal and distal colon have been reported.^49,50^ The regional differences also play a pivotal role in disease formation and progression. It is, for example, known that patients with colorectal cancer (CRC) on the right side (proximal) have a worse prognosis compared to those with CRC on the left side (distal).^51^ Inflammatory bowel diseases (IBD) can, for example, affect the complete gastrointestinal tract (Crohn’s disease) or specifically target the colon (ulcerative colitis).^52^ The mechanism behind is not fully clear. Starting with healthy wild-type mice, we wanted to generate a foundation of the molecular differences between the murine PC and DC epithelium. To our knowledge, there is no metabo-lipid-proteomics data set from mouse colon tissue currently available.

## Experimental Section

### Material and Methods

Metabolites from various groups of amino acids (AA), acylcarnitines (AC), nucleotides (NUC), organic acids (OA), short chain fatty acids (SCFA), and free fatty acids (FFA) were purchased from different suppliers such as Merck (Darmstadt, Germany), Toronto Research Chemicals (North York, Canada), and Cayman Chemicals (Ann Arbor, MI, USA). Stable isotopically labeled internal standards (SIL-ISs) of AA, AC, NUC, OA, SCFA, and FFA were prepared from Merck (Darmstadt, Germany), Cayman Chemicals (Ann Arbor, MI, USA), Toronto Research Chemicals (North York, Canada), and Cambridge Isotope Laboratory (MA, USA). Mouse SPLASH LIPIDOMIX Mass Spec Standard [Single-vial Prepared Lipidomic Analytical Standard in 1:1 dichloromethane:methanol (DCM: MeOH, 50:50 (*v*/*v*)] containing 14 SIL-ISs was obtained from Avanti Polar Lipids (AL, USA) (Table S1).

Solvents [acetonitrile (ACN), isopropanol (IPA), and methanol (MeOH)] used for UHPLC separation were of LC–MS grade and purchased from Honeywell (Offenbach, Germany). Formic acid (FA), acetic acid (AcOH), ammonium formate solution (BioUltra, 10 M in H_2_O), ammonium acetate solution BioUltra (NH_4_Ac; 5 M in H_2_O), methyl *tert*-butyl ether (MTBE), 3-nitrophenylhydrazine hydrochloride (3-NPH), *N*-(3-(dimethylamino)-propyl)-*N*′-ethylcarbodiimide hydrochloride (EDC), pyridine, ethylenediaminetetraacetic acid (EDTA), urea, ammonium bicarbonate, dithiothreitol (DTT), 2-chloroacetamide (CAA), and dimethyl sulfoxide (DMSO) were supplied by Merck (Darmstadt, Germany). Pierce Bicinchoninic Acid (BCA) Protein Assay Kits were prepared from Thermo Fisher Scientific Inc. (Waltham, MA, USA).

### Preparation of Stock Solutions, Internal Standard (SIL-IS), Quality Control Samples, and Calibration Curves

All information about the concentration ranges of the AAs, ACs, NUCs, OAs, SCFAs, and FFAs stock solutions, high-concentrated mix solutions for each group of metabolites, the relevant SIL-ISs solutions, the concentration levels of the calibration curves, as well as preparation of QC samples is given in the Material and Methods section of the Supporting Information.

### Mouse Colon Tissue Collection and Preparation

All animal experiments, maintenance, and breeding of mouse lines were approved by the Committee on Animal Health Care and Use of the state of Upper Bavaria (Regierung von Oberbayern: AZ ROB-55.2–2532.Vet_02–20–58). To generate homogeneous test samples for the method optimization, the entire small intestine and colon were excised from a wild-type mouse. The intestine was flushed with phosphate-buffered saline (PBS, pH 7.4) to remove all intestinal content. The intestine was snap-frozen with liquid nitrogen, ground with mortar and pestle, and 20 mg-aliquots were weighed into Precellys Lysing kits (2 mL tube prefilled with ceramic beads) (Bertin Technologies SAS, France) for optimization of the extraction solvents.

To study the molecular structure of different colon locations, the optimized protocol was applied to colon tissues from four age-matched wild-type mice (2 males and 2 females) from the proximal (PC) and distal (DC) colon. All colonic samples were washed with phosphate-buffered saline (PBS, pH 7.4) and weighed into Precellys Lysing kits (2 mL tube prefilled with ceramic beads) (Bertin Technologies SAS, France) (Table S2). To evaluate the reproducibility, three tissue samples from each colon region from each mouse were analyzed (24 samples in total).

### Optimization of the Extraction Procedure

On the basis of the recent papers on multiomics applications, the MTBE extraction method was selected. To test the extraction efficiency for metabolites, lipids, and proteins, different solvent ratios were evaluated.^10^ The solvent ratio 10:3:2.5 *v*/*v*/*v* is based on the original Matyash et al. paper.^7^ Abbott et al.^53^ used 0.15 M ammonium acetate solution to increase the partition coefficient, improve phase separation, and reduce the loss of lipids. On the basis of initial tests, by increasing concentration until 0.3 M, lipids intensity improved and then leveled off (data not shown). To be able to quantify the acetate concentration in colon tissue, we used ammonium chloride as salt. In order to find the best method covering both polar and nonpolar metabolites and lipids, we also checked the ratio 7:3:2.5 *v*/*v*/*v* in the presence and absence of ammonium chloride. Sostare et al.^10,54^ showed that smaller volume ratios of MTBE can result in the better recovery of polar metabolites. However, initial tests indicated a considerable reduction of lipid intensities by using the ratios of 5:3:2.5 *v*/*v*/*v* and 3:3:2.5 of MTBE/MeOH/H_2_O; so, they were discarded (data not shown).

In total, four MTBE extraction conditions were tested [(A) 10:3:2.5 *v*/*v*/*v* of MTBE/MeOH/H_2_O; (B) 10:3:2.5 *v*/*v*/*v* of MTBE/MeOH/NH_4_Cl (0.3 M); (C) 7:3:2.5 *v*/*v*/*v* of MTBE/MeOH/H_2_O; and (D) 7:3:2.5 *v*/*v*/*v* of MTBE/MeOH/NH_4_Cl (0.3 M)].

Each condition consisted of two steps of extraction (Ex) and re-extraction (Re-Ex). For 20 mg of the homogenized tissue, the following solvent ratios were used. (A) Ex: 225 μL MeOH/750 μL MTBE/188 μL H_2_O; Re-Ex: 60 μL MeOH/200 μL MTBE/50 μL H_2_O; (B) Ex: 225 μL MeOH/750 μL MTBE/188 μL NH_4_Cl (0.3 M); Re-Ex: 60 μL MeOH/200 μL MTBE/50 μL NH_4_Cl (0.3 M); (C) Ex: 225 μL MeOH/525 μL MTBE/188 μL H_2_0; Re-Ex: 60 μL MeOH/140 μL MTBE/50 μL H_2_0; (D) Ex: 225 μL MeOH/525 μL MTBE/188 μL NH_4_Cl (0.3 M); Re-Ex: 60 μL MeOH/140 μL MTBE/50 μL NH_4_Cl (0.3 M). All steps of tissue homogenization, extraction, and reconstitution were performed according to the next section. Two pre-extraction spiked samples and two postextraction spiked samples were prepared for each extraction condition (prespiking and postspiking refer to adding the SIL-ISs for metabolites and lipids before and after sample preparation at the same concentration levels, respectively). The concentration ranges of the spiked SIL-ISs were fixed as 10 to 50 μM for 27 SIL-IS-AAs and IS carnitine; 0.1 to 1 μM for 9 SIL-IS-ACs; 2 to 5 μM for 11 SIL-IS-nonphosphorylated-NUCs; 20 μM for 5 SIL-IS-phosphorylated-NUCs; 50 μM for SIL-IS-SCFAs; 16 μM for 7 SIL-IS-OAs, 5 μM for 12 SIL-IS-FFAs; and 0.2 to 25 μM for Mouse SPLASH LIPIDOMIX standard (containing 14 IS and minimum one per lipid class). For the preparation of pre-extraction spiked samples, all SIL-ISs were spiked in MeOH of the EX step. Postextraction spiked samples for metabolomics studies were obtained by spiking SIL-IS-AAs, SIL-IS-ACs, SIL-IS-OAs, SIL-IS-SCFAs, and SIL-IS-NUCs in the reconstitution solvent of 225 μL ACN:H_2_O (50:50, *v*/*v*). Postextraction spiked samples for lipidomics studies were prepared by spiking SIL-IS-FFAs, SIL-IS-SCFAs, and SIL-IS-ACs, and Mouse SPLASH LIPIDOMIX standard in the reconstitution solvent of 225 μL ACN:IPA (50:50, *v*/*v*). Pre- and postextraction samples were measured by using LC–MS-based targeted methods (“Targeted Metabolomics” section of the Supporting Information and Tables S3–S7). SIL-ISs of AAs, ACs (except IS C14 and IS C16), NUCs, OAs, and SCFAs (except IS HA and IS ICA) were measured in the aqueous phase (MeOH and H_2_O), while Mouse SPLASH LIPIDOMIX standard (14 SIL-ISs) and SIL-ISs of 12 FFAs, 2ACs (IS C14:0 and C16:0 L-carnitine), and 2 SCFA (IS HA and IS ICA) were measured in the MTBE phase.

### Proximal and Distal Colon Tissue Extraction

The extraction system (MTBE/MeOH/ammonium chloride (0.3 M); 10/3/2.5, *v*/*v*/*v*) was applied for the multiomics extraction workflow. First, 20 mg of colon tissue was transferred to Precellys Lysing Kits (2 mL tube prefilled with ceramic beads) followed by adding 225 μL methanol with SIL-ISs of the metabolites and lipids. (Table S2). For tissue samples more or less than 20 mg, the solvent volumes were adjusted. Internal standard concentrations were 10 to 50 μM for SIL-IS-AAs, 0.2 μM for SIL-IS-ACs (except IS carnitine and IS C2, they were fixed as 20 and 2 μM, respectively), 2 to 5 μM for SIL-IS-nonphosphorylated-NUCs, 20 μM for SIL-IS-phosphorylated-NUCs, 1.25 μM for SIL-IS of FFAs, 50 μM for SIL-IS of SCFA, and 16.5 μM for SIL-IS of OA. For mouse SPLASH LIPIDOMIX Mass Spec Standard, a 1:10 dilution was fixed for colon tissue samples, which provided good intensity for all spiked SIL-ISs of lipids. After tissue homogenization in a FastPrep-24 5G Homogenizer (MP Biomedicals, France) at 6000 rpm for 2 × 20 s (20 s break) and in the presence of dry ice, MTBE (750 μL per 20 mg tissue) was added for lipid extraction, followed by shaking for 1 h with ThermoMixer C (Eppendorf AG, Hamburg, Germany) at room temperature (900 rpm). Next, phase separation was induced by adding 188 μL NH_4_Cl (0.3 M) per 20 mg tissue, letting sit for 20 min at room temperature in the ThermoMixer (900 rpm). After centrifugation (10 min at 14,000*g*; *T*: 4 °C) with centrifuge 5424 R (Eppendorf AG, Hamburg, Germany), the upper phase (MTBE containing lipid) was transferred to a fresh reaction tube (2 mL), and the pellet with the remaining aqueous phase was used for the re-extraction of lipids. The mixture of MTBE (200 μL), MeOH (60 μL), and NH_4_Cl (0.3 M) (50 μL) (to keep the volumetric solvent ratio of 10:3:2.5) was added to the remaining aqueous phase and pellet from extraction step. The mixture was mixed with ThermoMixer for 20 min at 900 rpm (at room temperature). Afterward, it was centrifuged for 10 min at 14,000*g* at 4 °C, and the upper phase was transferred to the 2 mL reaction tube containing MTBE from the extraction step 1. The aqueous phase was also placed in another reaction tube, and the pellet was kept at −80 °C for the proteome analysis. The total MTBE and aqueous phases resulting from both extraction and re-extraction steps were evaporated by using a vacuum concentrator (45 min at 30 °C in a CentriVAP Concentrator [LabConco, Kansas-City, MO, USA)]. The final residues of MTBE phase were reconstituted in 225 μL of ACN:IPA (50:50, *v*/*v*) and the aqueous phases in 225 μL of ACN:H_2_O (50:50, *v*/*v*) for LC–MS/MS measurement. Due to the high concentration of some FFAs in DC and PC tissues, a small portion of lipidomics samples was diluted in a 1:3 (*v*/*v*) ratio with ACN:IPA (50:50, *v*/*v*) and then measured with the free fatty acid LC–MS/MS. For targeted analysis of SCFA and OA, an extra step of derivatization with 3-NPH in the presence of EDC was done.^18,20^ Briefly, 10 μL of 200 mM 3-NPH in ACN/H_2_O (50/50, *v*/*v*) and 10 μL of 120 mM EDC in ACN/H_2_O (50/50, *v*/*v*) containing 6% pyridine was added to 30 μL of both aqueous and MTBE phases. The mixtures were incubated at 40 °C for 30 min under constant shaking with ThermoMixer C (500 rpm). Then, they were filled to 0.5 mL with ACN/H_2_O (50/50, *v*/*v*), followed by measurement with SCFA and OA LC–MS/MS.^18,20^

The remaining pellet was reconstituted with 450 μL of Lysis buffer containing 8 M urea, 5 mM EDTA, 100 mM ammonium bicarbonate, and 1 mM DTT. The mix was homogenized in a FastPrep-24 5G Homogenizer at 6000 rpm for 2 × 20s with 20 s breaks. Next, protein concentration was determined by BCA assay according to the manufactures protocol^55^ by Tecan Infinite 200 PRO (Männedorf, Switzerland) at a wavelength of 562 nm and a bandwidth of 9 nm. Afterward, in solution, trypsin digestion was done with 15 μg of total protein amount. To do this, 15 μg of proteins was reduced and alkylated with 10 mM DTT for 30 min at 30 °C under constant shaking by thermomixer (400 rpm) and 55 mM CAA for 30 min in the dark at room temperature. Samples were diluted with 50 mM ammonium bicarbonate to a final concentration of 1.33 M urea, and then trypsin was added in a 1:100 enzyme/protein ratio for the first step of digestion at 30 °C by using thermomixer (400 rpm). After 2 h, trypsin was again added (in a final ratio of 1:50), followed by incubation at 30 °C overnight under constant shaking by thermomixer (400 rpm).

Afterward, samples were acidified to a final concentration of 1% FA and transferred to the next step of cleanup and desalting by using C18 tips. Tips were packed in-house with three Empore C18 disks. After column conditioning [in three steps by adding (a) ACN, (b) ACN:H_2_O (4:6, *v*/*v*) containing 0.1% FA, and (c) ACN:H_2_O (2:98, *v*/*v*) containing 0.1% FA with centrifuging at 1500 rcf for 2 min in between], the samples were slowly loaded by centrifuging at 500*g* for 5 min followed by reapplying and centrifuging. Then, column washing was done using ACN:H_2_O (2:98, *v*/*v*) containing 0.1% FA and centrifuging at 1500*g* for 2 min. Elution of proteins was done twice by using a total volume of 80 μL of ACN:H_2_O (4:6, *v*/*v*) containing 0.1% FA and centrifuging at 500*g* for 5 min. The collected samples were evaporated using a vacuum concentrator at 30 °C and redissolved in 30 μL ACN:H_2_O (2:98, *v*/*v*) containing 0.1% FA. All samples were transferred to a 96-well plate and injected into the nanoLC coupled to Q-Exactive HF-X mass spectrometer.

### Targeted Metabolomics

For targeted analysis, a QTrap 5500 triple quadrupole mass spectrometer coupled to an ExionLC UPLC system (both Sciex, Darmstadt, Germany) was used in the multiple reaction-monitoring mode (MRM). Instrumental control was performed with Analyst 1.7.2 software (Sciex, Darmstadt, Germany). The respective MRM-transitions are listed in Table S3 for amino acids and acylcarnitines,^17^Table S4 for nucleotides and nucleosides,^21^Table S5 for short chain fatty acids and organic acids,^18,20^Table S6 for free fatty acids,^22^ and Table S7 for the Mouse SPLASH Lipidomix Mass Spec Standard. The respective chromatographic separation are summarized in the “Targeted metabolomics – UPLC settings” section of the Supporting Information, and example chromatograms are depicted in Figure S5.

Quantitative LC–MS/MS data analysis was done by MultiQuant 3.0.3 (Sciex, Darmstadt, Germany). Calibration curves were obtained according to a linear regression model with weighting type 1/x (except Lac, weighting type, none). Table S9 summarizes the validation parameters listing the linear range and limit of quantitation for each metabolite. Table S10 summarizes the precision and accuracy of the measured QC-samples. The metabolite concentrations were calculated as nmol per gram of tissue and are listed in Table S11.

### Untargeted Metabo-Lipidomics-Proteomics Measurements

Untargeted metabolomics and lipidomics measurements were performed with a TripleTOF 6600 high-resolution quadrupole-time-of-flight (QTOF) mass spectrometer (Sciex, Darmstadt, Germany) coupled to a Nexera UHPLC system (Shimadzu, Duisburg, Germany). The data was recorded in the data-dependent acquisition mode (DDA) and the instrument controlled with Analyst TF 1.7.1 software (Sciex, Darmstadt, Germany).

For the untargeted analysis of metabolites, the HILIC chromatographic conditions were as follows:^26^ column and precolumn, Acquity UPLC Premier BEH Amide (130 Å, 1.7 μm, 2.1 mm × 100 mm) connected to VanGuard UPLC BEH Amide precolumn (130 Å, 1.7 μm, 2.1 mm × 5 mm) (Waters Co., MA, USA); mobile phase composition, solvent A: H_2_O containing 5 mM ammonium acetate and solvent B: ACN:H_2_O (95:5, *v*/*v*) containing 5 mM ammonium acetate; gradient program, 0 min, 100% B; 1.5 min, 100% B; 8 min, 60% B; 10 min, 20% B; 11.5 min, 20% B; 12 min, 100% B; 15 min 100% B; flow rate, 0.4 mL/min; injection volume, 5 μL; column oven temperature, 40 °C. Parameters of the autosampler: temperature, 10 °C; rinsing solvent for all channels R0, R1, R2, 10% IPA; rinse type, outer surface of the needle before and after aspiration; rinsing speed, 35 μL/s; rinsing volume, 500 μL. MS settings in the positive (negative) mode were as follows: gas 1 55 psi, gas 2 65 psi, curtain gas 35 psi, temperature 500 °C, ion spray voltage 5500 (−4500) V, declustering potential 80 (−80) V, mass range of the TOF MS and MS/MS scans 50–2000 *m*/*z*, and the collision energy 35 (−35) V with a 20 V spread. The DDA-setting were as follows: the 8 most intense ions were fragmented; after three occurrences, the precursor ions was put for 10 s on an exclusion list.

For the lipidomics measurements, we used a HILIC and RP method based on refs (25 and 56) with some modifications.

HILIC conditions: column and precolumn, Acquity UPLC Premier BEH Amide (130 Å, 1.7 μm, 2.1 mm × 100 mm) connected to VanGuard UPLC BEH Amide precolumn (130 Å, 1.7 μm, 2.1 mm × 5 mm) (Waters Co., MA, USA); mobile phase composition, solvent A: H_2_O:ACN (50:50, *v*/*v*) containing 5 mM ammonium acetate (pH:8) and solvent B: ACN:H_2_O (95:5, *v*/*v*) containing 5 mM ammonium acetate (pH:8); gradient program, 0 min, 99.9% B; 5 min, 99.9% B; 15 min, 95% B; 17 min, 5% B; 21 min, 5% B; 21.5 min, 99.9% B; 26 min, 99.9% B; flow rate, 0.3 mL/min; injection volume, 5 μL; column oven temperature, 40 °C. RP conditions: column and precolumn, Acquity UPLC Premier BEH C18 (1.7 μm, 2.1 mm × 100 mm) connected to VanGuard UPLC BEH C18 precolumn (1.7 μm, 130 Å, 2.1 mm × 5 mm) (Waters Co., MA, USA); mobile phase composition, solvent A: H_2_O:ACN (40:60, *v*/*v*) containing 10 mM ammonium formate and 0.1% formic acid and solvent B: IPA:ACN:H_2_O (45:55:5, *v*/*v*) containing 10 mM ammonium formate and 0.1% formic acid; gradient program, 0 min, 32% B; 1.5 min, 32% B; 12 min, 85% B; 21 min, 97% B; 25 min, 97% B; 25.1 min, 32% B; 30 min, 32% B (gradient program for pos mode, 0 min, 32% B; 1.5 min, 32% B; 12 min, 85% B; 21 min, 100% B; 30 min, 100% B; 30.5 min, 32% B; 35 min, 32% B); flow rate, 0.3 mL/min; injection volume, 5 μL (for positive mode, 1 μL); column oven temperature, 40 °C. Autosampler conditions for untargeted lipidomics in negative mode were fixed as rinsing solvent for all channels R0, R1, R2, 10% IPA; rinse type, outer surface of the needle for before and after aspiration; rinsing speed, 35 μL/s; rinsing volume, 500 μL; autosampler temperature, 10 °C. For untargeted lipidomics based on RP-LC separation in positive mode, parameters of autosampler were as follows: rinsing solvent for channel R0, 10% IPA; rinsing solvent for channels R1 and R2, IPA; rinse type, internal and external surface of the needle for before and after aspiration; rinse dip time, 20 s; rinsing speed, 35 μL/s; rinsing volume, 500 μL; and autosampler temperature, 10 °C. MS setting on the untargeted lipidomics based on DDA measurements in the positive (negative) mode were the same as for the untargeted metabolomics.

For untargeted proteomics, a nanoflow LC–MS/MS setup comprised of a Dionex Ultimate 3000 RSLCnano system coupled to a Q-Exactive HF-X mass spectrometer (Thermo Fisher Scientific Inc.) was used in positive ionization mode. MS data acquisition was performed in data-dependent acquisition (DDA) mode. For complete proteome analyses, 0.5 μg of peptides were delivered to a trap column (ReproSil-pur C18-AQ, 5 μm, Dr. Maisch, 20 mm × 75 μm, self-packed) at a flow rate of 5 μL/min in HPLC grade water with 0.1% (*v*/*v*) formic acid. After 10 min of loading, peptides were transferred to an analytical column (ReproSil Gold C18-AQ, 3 μm, Dr. Maisch, 450 mm × 75 μm, self-packed) and separated using a 120 min linear gradient from 4% to 32% of solvent B (0.1% (*v*/*v*) FA and 5% (*v*/*v*) DMSO in acetonitrile) at 300 nL/min flow rate. The nano-LC solvent A was 0.1% (*v*/*v*) FA and 5% (*v*/*v*) DMSO in HPLC-grade water. MS1 spectra (360–1300 *m*/*z*) were recorded at a resolution of 60,000 using an automatic gain control target value of 3 × 10^6^ and a maximum injection time of 45 ms. Up to 18 peptide precursors were selected for fragmentation. Only precursors with charge state 2 to 6 were selected and dynamic exclusion of 25 s was enabled. Peptide fragmentation was performed using higher energy collision dissociation (HCD) and a normalized collision energy of 26%. The precursor isolation window width was set to 1.3 *m*/*z*. MS2 Resolution was 15.000 with an automatic gain control target value of 1 × 10^5^ and a maximum injection time of 25 ms.

### Untargeted data processing

#### Untargeted Metabo-Lipidomics Data Processing

All LC–MS/MS-DDA raw files obtained from untargeted metabo-lipidomics were processed by MS-DIAL (version 4.70).^29,36^ Before starting untargeted data processing, the alignment of 3 QCs (at the beginning, middle, and end of analytical batch) and samples were checked based on retention times and peak intensities using PeakView software (version 2.2) (Sciex, Darmstadt, Germany) (Figures S1, S2, and S3). Next, the raw data (“Wiff” format obtained by AB Sciex) are first converted into a “ABF” format by ABF File Converter. Data processing by MS-DIAL was performed based on peak picking, deconvolution, identification, adduct type, and peak alignment. Feature collection was done based on MS1 and MS2 in the range from 50 to 2000 Da. The MS1 and MS2 tolerances were set to 0.01 and 0.025 Da, respectively, in the centroid mode. Minimum peak height detection was fixed at 1000 amplitude, and identification was performed with accurate MS1 and MS2 tolerances of 0.01 and 0.05 Da, respectively. The alignment setting was fixed by a QC-sample as a reference file with retention time tolerance of 0.8 min and a MS1 tolerance of 0.015 Da. Lipid and metabolite data normalization was done based on Mouse SPLASH LIPIDOMIX Mass Spec Standard and total ion current (TIC), respectively, followed by multivariate analyses by PCA to gain insight into the classification power of data sets. The normalized data for each untargeted method in both negative and positive modes was merged and used for further statistical analyses.

#### Untargeted Proteomics Data Processing

The untargeted proteomics data were searched against the UniProt *Mus musculus* proteome (UP000000589, downloaded July 2020, 17038 protein entries) using MaxQuant (version 1.6.3.4) with its built-in search engine Andromeda.^40,57^ As a parameter setting, Trypsin/P was specified as a proteolytic enzyme. Precursor tolerance was set to 4.5 ppm and fragment ion tolerance to 20 ppm. The minimal peptide length was defined as seven amino acids, and the “match-between-run” function was disabled. For full proteome analyses, carbamidomethylated cysteine was set as fixed modification and oxidation of methionine and N-terminal protein acetylation as variable modifications. Results were adjusted to a 1% false discovery rate (FDR) on peptide spectrum match (PSM) and protein level employing a target-decoy approach using reversed protein sequences. Label-free protein quantification intensities (LFQs) were used for protein quantification, with at least 2 peptides per protein identified.^58^ From the final data set, proteins that were specified as “only identified by site”, “potential contaminants”, and “reversed” were filtered out. Further, only proteins for which at least 2 out of 3 technical replicate measurements provided a LFQ value were considered. Missing values were imputed with a constant value that represented half of the lowest detected LFQ intensity per protein. However, if the imputed value was higher than the 20% quantile of all LFQ intensities in the data set, we used the 20% quantile as an imputed value.

Proteomic analysis was performed by using in-house software based on below criteria (https://github.com/mengchen18/omicsViewer). For differential abundance analysis, LFQ protein intensity values were logarithm transformed (base 10) and a Student’s *t* test was applied to identify proteins differentially expressed between PC and DC tissue (Table S18). The resulting *p*-values were adjusted by the Benjamini-Hochberg algorithm^59^ to control the false discovery rate (FDR). All proteins with a fold-change > 2 and an adjusted *p*-value < 0.05 were considered as significant.

### Statistical Pathway Analyses

Statistical analyses based on univariate and multivariate methods for both targeted and untargeted data and quantitative enrichment analysis (QEA) of targeted data were conducted by using MetaboAnalyst 5.0 or 6.0.^34^ To do this, targeted/untargeted data was log-transformed and Pareto-scaled. Pathway detection of targeted and untargeted metabolomics data was performed by using the KEGG database. Pathway detection of untargeted proteomics data based on over-representation analysis (ORA) was performed by in-house software. Lipid and FFA pathway detection was done with BioPAN,^43,60^ which is available on the LIPIDMAPS Lipidomics Gateway (https://www.lipidmaps.org/). By inclusion of LipidLynxX, lipid data sets from different nomenclatures and levels of structural information can be processed by BioPAN. The normalized HILIC- and RP-based untargeted lipidomics data sets were uploaded to the BioPAN Web site and data processing was performed based on the annotation level of “sum composition’ (lipid species).

In order to find the linkage of detected metabolites, and proteins within their biological context, significant proteins (as Uniprot protein ID) and metabolites (HMDB ID; KEGG Compound ID) were applied in the “Joint pathways analysis” module in Metboanalyst 5.0 and uploaded to Pathview for a better visualization of the Kegg-pathways.^44^ The mouse genome database (*Mus musculus*) was selected, and data processing was started by matching genes/proteins and compounds with the underlying databases. All integrated pathways, including metabolic and gene pathways were considered. The pathway enrichment analysis and pathway topology based on degree centrality was integrated according to “Combine *p* values (overall)”, which applies fixed weights based on the overall proportions of genes and metabolites in the combined universe across all pathways.

## Results and Discussion

**Figure 1:**
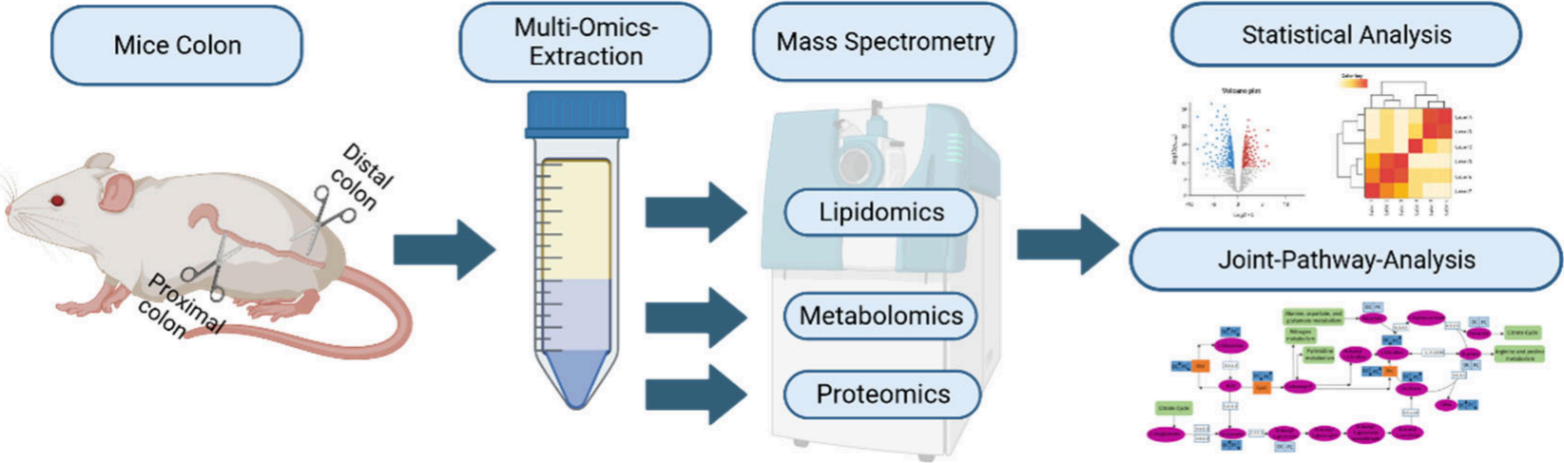
Workflow of a multiomics extraction.

The multiomics extraction methods for metabolites, lipids, and proteins were first optimized and then applied to epithelial tissue from the proximal and distal colon of healthy mice. Figure 1 depicts the metabo-lipid-proteomics workflow from the sample extraction, to the mass spectrometric and statistical analysis and the combination of all omics-data.

### Optimization and Validation of Multiomics Extraction

To develop the metabo-lipid-prote-omics method, first the sample preparation from a single tissue biopsy was optimized. We chose the MTBE extraction protocol which is based on the addition of MeOH and then MTBE to the sample to extract lipids and metabolites and precipitate proteins, followed by the addition of water for phase separation.^7^ However, significant carryover of water into the MTBE-phase (solubility: in water at 20 °C: 4–5%) can increase ion suppression and adduct formation during the MS-measurements due to the coextraction of salts and other metabolites.^6^ This influences the reproducibility of sample preparation, quantitative results, especially for amphiphilic and polar compounds, and thus the accurate multiomics analyses. To address these issues, initial experiments were performed on the MTBE extraction method based on the presence of three types of salt (ammonium formate, ammonium acetate, and ammonium chloride at different concentration levels) and their absence. Saline solution better separated the two miscible solvents (MTBE and MeOH) and efficiently extracted amphiphilic and polar compounds. However, large amounts of acetate and formate acted as interfering agents in the 3-NPH-derivatization reactions and thus adversely affected the detection of SCFA/OA-3-NPH derivatives (data not shown). So, ammonium chloride was selected for the extraction optimization. Briefly, 20 mg-aliquots of the homogenized intestine were extracted with different solvent ratios in the presence and absence of salt:^7,10,53^ [(A) 10:3:2.5, *v*/*v*/*v* of MTBE/MeOH/H_2_O; (B) 10:3:2.5, *v*/*v*/*v* of MTBE/MeOH/NH_4_Cl (0.3 M); (C) 7:3:2.5, *v*/*v*/*v* of MTBE/MeOH/H_2_O; and (D) 7:3:2.5, *v*/*v*/*v* of MTBE/MeOH/NH_4_Cl (0.3 M)]. The validation of the method was performed using the SIL-IS because analyte-free mice tissue for spiking experiments is not available. In the later experiments, the SIL-IS was used to correct the recovery and matrix effect of the analytes of interest.

To select the best extraction conditions, recovery efficiency (RE), matrix effect (ME), and process efficiency (PE) were calculated for each spiked SIL-IS by the following equations.^61,62^
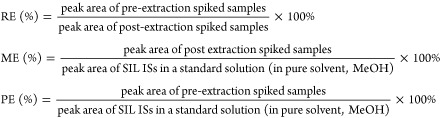


**Figure 2:**
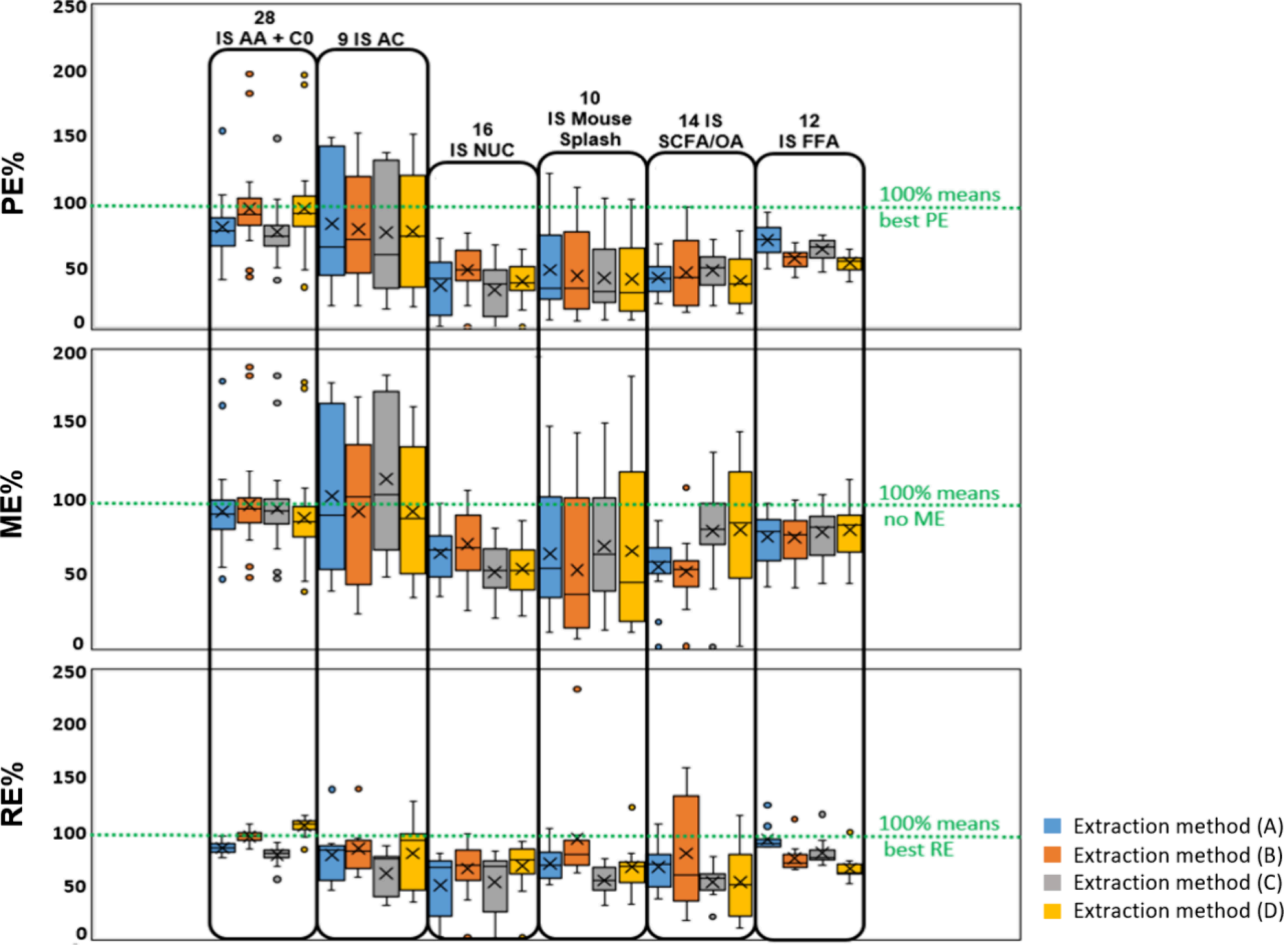
Box and Whisker Plots representing RE%, ME%, and PE% values for SIL-ISs of metabolites and lipids by using four extraction methods [(A) 10:3:2.5, *v*/*v*/*v* of MTBE/MeOH/H_2_O (with blue color); (B) 10:3:2.5, *v*/*v*/*v* of MTBE/MeOH/NH_4_Cl (0.3 M) (with orange color); (C) 7:3:2.5, *v*/*v*/*v* of MTBE/MeOH/H_2_O (with gray color); and (D) 7:3:2.5, *v*/*v*/*v* of MTBE/MeOH/NH_4_Cl (0.3 M) (with yellow color); AA, amino acid; C0, carnitine; AC, acylcarnitine; NUC, nucleotides/nucleosides; SCFA, short chain fatty acid; OA, organic acid; FFA, free fatty acid; RE%, recovery efficiency; ME%, matrix effect; and PE%, process efficiency).

RE demonstrates the extraction efficiency of a method and indicates how many analytes have been lost during sample preparation. ME is an indicator for the matrix influence on ionization of the analytes. PE takes into account both the recovery (RE%) and the matrix effect (ME%) based on following equation:
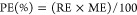


Figure 2 displays box plots summarizing the RE%, ME%, and PE% for each group of tested metabolites and lipids (SIL-ISs spiked) for four extraction conditions. The obtained values of RE%, ME%, and PE% for each single SIL-IS as well as the mean ± SD values of RE% and PE% for each group of SIL-ISs of metabolites and lipids are listed in Table S8.

For SIL-IS AA + C0, SIL-IS AC, SIL-IS Nuc, and SIL-IS Mouse Splash, we saw that the addition of salt to the extraction solvent improves the RE% and with that also the overall PE%. Acylcarnitines and the lipids of the Mouse SPLASH demonstrate a high variation in the ME% which is most likely due to their amphiphilic character. For the rest (NUCs, OAs, and SCFAs), high variation can be justified due to the various polarity within each group of metabolites. Method optimization based on PE% indicated that method B [10:3:2.5, *v*/*v*/*v* of MTBE/MeOH/NH_4_Cl (0.3 M)] was able to provide a better extraction especially for polar metabolites (92.3%, 44.8%, and 42.5% for SIL-ISs of AA, NUC, and SCFA/OA, respectively). In addition, obtained PE% values for other groups of SIL-ISs (67.3% for SIL-IS-ACs, 40.4% for Mouse SPLASH LIPIDOMIX standards, and 53.7% for SIL-IS-FFAs) were also acceptable.

In conclusion, the use of NH_4_Cl (0.3 M) as the separation agent in the MTBE extraction and the solvent ratio of 10:3:2.5, *v*/*v*/*v* for MTBE/MeOH/NH_4_Cl (0.3 M) represented the optimal workflow to achieve a relatively high PE%, RE%, and small ion-suppression effects (ME%), especially for polar metabolites. With regard to the proteome, a close similarity in the total number of quantified proteins (around 3700) using conditions without or with ammonium salt was observed (Figure S4). In addition, the solvent ratios did not have a significant effect on protein recovery.

### Metabo-Lipid-Prote-Omics of Healthy Mouse Colon Tissue

After the successful optimization of the sample preparation, healthy mouse colon tissues from the proximal and distal region were studied to get a better picture of the molecular differences between the colon regions. To test the reproducibility of the method, three replicates per colon region from the same mouse were extracted. In total, four mice (2 male, 2 female) were studied.

#### Targeted Metabolomics of Healthy Mouse Colon Tissue

**Figure 3:**
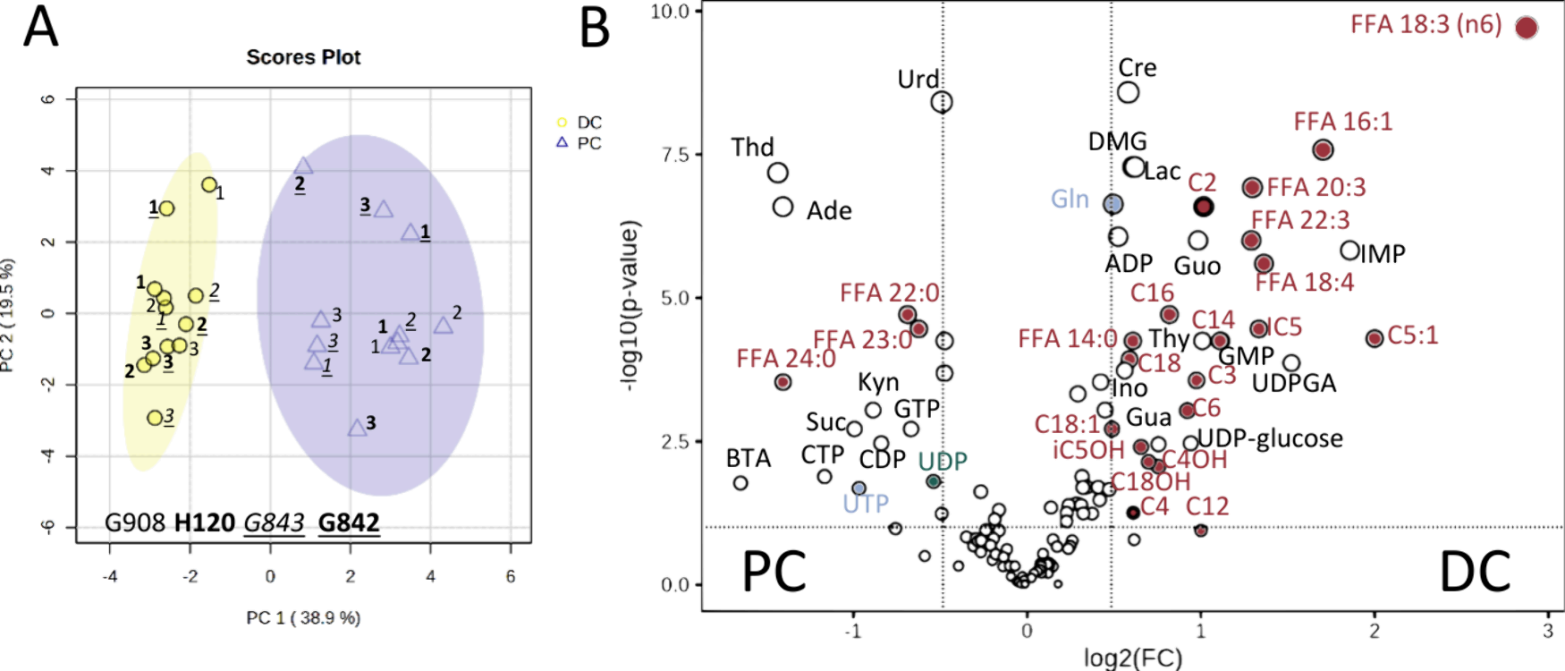
(a) PCA score plot of targeted metabolomics data of two healthy colon locations of mice (DC: distal colon, PC: proximal colon; G908, H120, G843, and G842 are the respective mouse IDs); (b) volcano plot [fold change (FC) threshold: 1.4, P-value threshold: 0.05]. The names of the metabolites that play a role in the observed lipid metabolism are colored in red, those from the hexosamine biosynthesis pathway are colored in blue, and those from the mucin-O-glycan biosynthesis are colored in green (see Figure 8 and Figure 9).

With the targeted LC–MS methods, 122 compounds, including 32 AAs (out of 37), 22 ACs (out of 22), 27 NUCs (out of 33), 28 FFAs (out of 31), and 13 SCFAs and OAs (out of 24), were quantified in 24 mouse colon samples from two groups (*n* = 12 for each group of PC and DC, consisting of 4 biological replicates and 3 biopsies per colon region). The quantitative data is listed in nmol/g tissue in Table S11. Figure 3a shows the PCA score plot for the targeted metabolomics data of healthy mouse colons. A clear separation between the two colonic tissue locations could be observed. On the basis of the Mann–Whitney U test, the nonparametric test for unpaired analysis of two independent groups, 62 compounds (out of 122 measured in healthy colons) were determined as significant features. By considering FC > 1.4 alongside *p*-values cutoff < 0.05, 15, and 32 metabolites were significantly up and down-regulated in the DC tissues, depicted in Figure 3b. Significantly increased metabolites in DC tissues compared to the PC tissue consisted of 3 AAs (Cre, DMG, Gln), 13 ACs (C2, C3, C4, C4OH, C5:1, IC5, iC5OH, C6, C14, C16, C18, C18:1, and C18OH), 6 FFAs [14:0, 16:1(9*Z*), 18:3-n6, 18:4-n3, 20:3-n6, and 22:3-n3], 9 NUCs (ADP, Guo, IMP, GMP, Thy, UDPGA, Ino, UDP-glucose, Gua), and 1 OA (Lac). Significantly decreased metabolites in the DC tissues were 2 AAs (Kyn, Cit), 3FFAs (22:0, 23:0, 24:0), 8 NUCs (Urd, Thd, Ade, GTP, CDP, CTP, UTP, UDP), 1 SCFA (BTA), and 1OA (Suc). Figure S6 shows a heatmap obtained based on Euclidean distance measure and Ward clustering algorithm with samples in columns and features in rows. It visually confirmed an acceptable hierarchical clustering of samples and features between two colon regions.

The metabolomics measurements revealed increased levels of several acyl-carnitines (C2, C3, C4, C4OH, C5:1, IC5, iC5OH, C6, C8, C10, C12, C14, C16, C18OH, and C20:4) as well as C0 in DC tissue. Acyl-carnitines are esters arising from the conjugation of fatty acids with L-carnitine. They are formed when acyl-CoAs are transported from the cytosol into the mitochondrial matrix for β-oxidation to generate energy in form of ATP.^63^ Higher levels of free fatty acids [C14, C16:1, C18:3 (9Z, 12Z, 15Z), C18:4, C20:3, and C22:3] have also been detected in DC tissue, whereas the ultralong-chain fatty acids (C22, C23, C24) were dominant in PC tissue. These findings might indicate toward a different energy demand and usage between the two different colon locations. In humans, higher levels of fecal ACs are a robust biomarker for IBD.^64^

#### Untargeted Metabolomics of Healthy Mouse Colon Tissue

**Figure 4:**
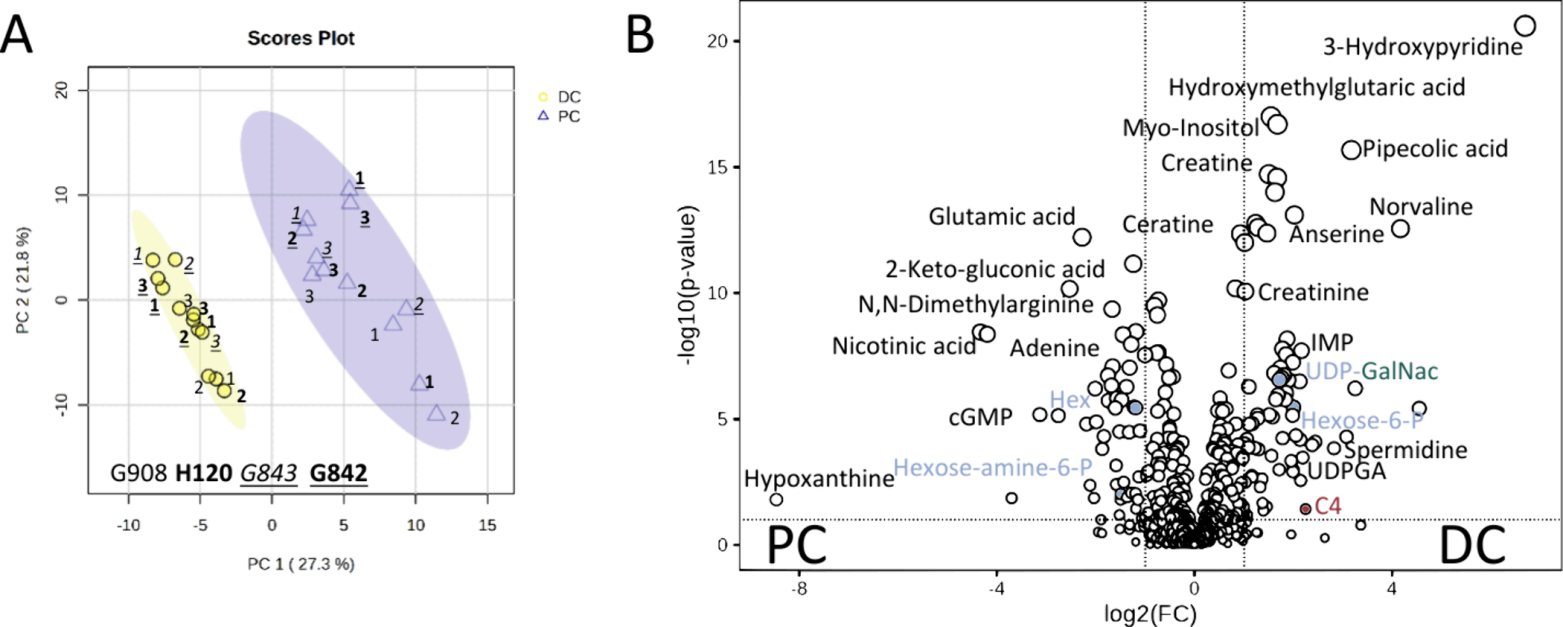
(a) PCA score plot for HILIC untargeted metabolomics data, and (b) volcano plot (FC threshold: 2, *p*-value threshold: 0.05) (DC: distal colon, PC: proximal colon, and G908, H120, G843, and G842 are the respective mouse IDs). The names of the metabolites that play a role in hexosamine biosynthesis pathway are colored in blue and those from the mucin-O-glycan biosynthesis are colored in green (see Figure 8 and Figure 9).

Additionally, the tissue biopsies were analyzed using untargeted LC–MS/MS in the data-dependent acquisition mode to gain additional information on the spatial regulated metabolic pathways in colon tissue. First, the untargeted metabolomics data were processed with MS-DIAL. Feature annotation was performed using their MS/MS-spectral library.^29^ Three-hundred and sixty-nine unique reference-matched metabolites have been identified in the untargeted HILIC-data sets (Table S12). The principal component analysis (PCA) of the HILIC untargeted metabolomics data is displayed in Figure 4a, showing a clear separation of the two colon regions. On the basis of fold change (FC) > 2 and *p*-values cutoff < 0.05, 100, and 73 features were significantly up and down-regulated in the distal colon which is shown in Figure 4b. Table S13 summarizes all metabolites significantly regulated in DC and PC tissue. A heatmap of this data is depicted in Figure S7. Appendix 1 in the Supporting Information depicts extracted ion chromatograms together with the respective MS/MS-spectra and its database hit.

To get an estimate about how much overlap exists between the targeted and untargeted data, a scatter plot was generated and correlation coefficient was determined (Figure S8). An acceptable correlation coefficient of 0.63 was achieved. The advantage of the targeted methods is that, for example, isobaric compounds like isoleucine and leucine are chromatographically separated and can be quantified. In addition, the LOD of the method is known and reference standards are always measured together with the sample set. The untargeted approach helps to generate new hypotheses and confirms hypotheses arising from the targeted approach by detecting additional metabolites that are differentially regulated within a given pathway. The combination of targeted and untargeted adds extra information to the global multiomics workflow.

#### Untargeted Lipidomics of Healthy Mouse Colon Tissue

**Figure 5:**
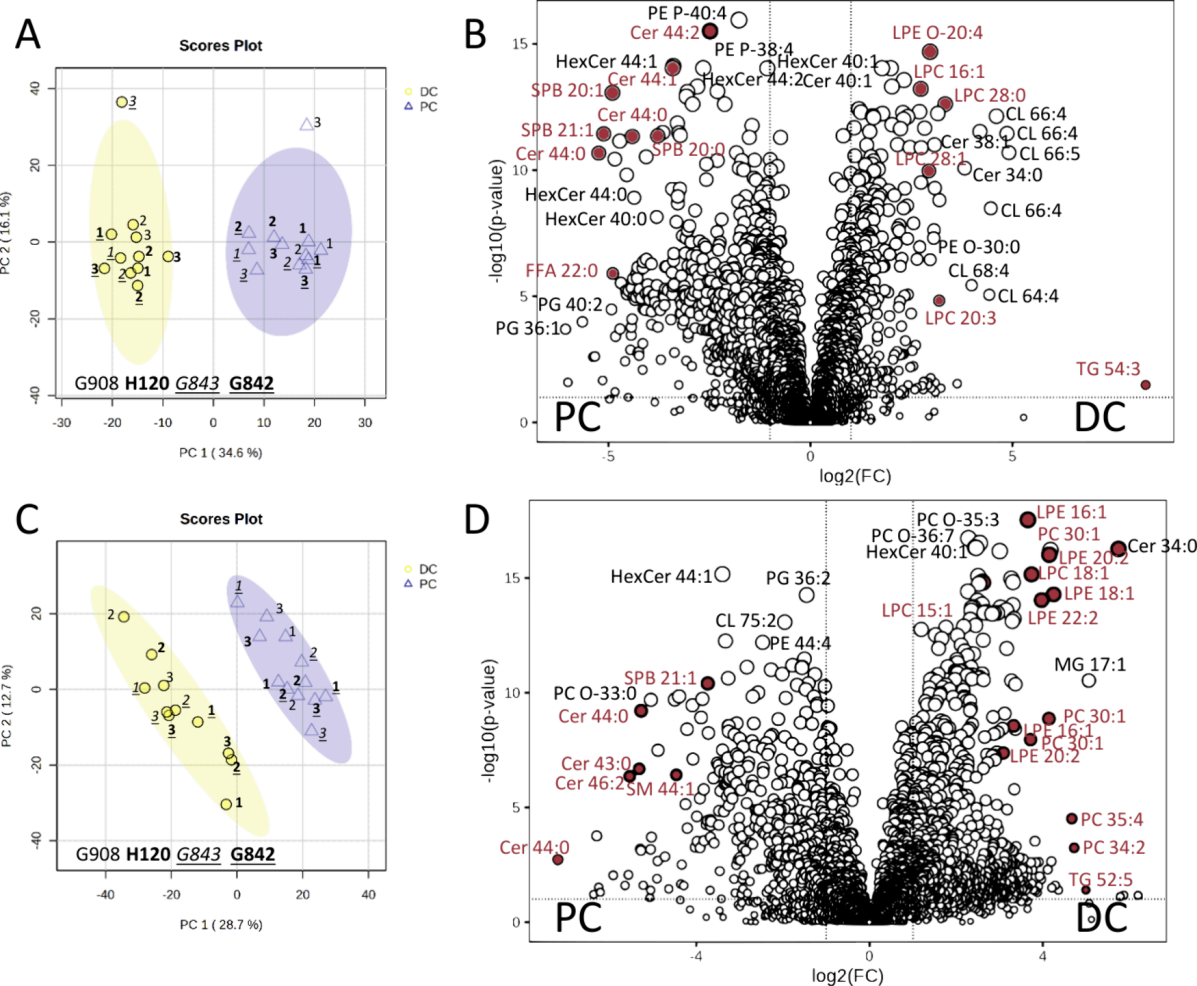
(a) PCA score plot and (b) Volcano plot for HILIC lipidomics measurement. (c) PCA score plot and (d) Volcano plot for RP lipidomics measurement (FC threshold of 2 and *p*-value threshold of 0.05; G908, H120, G843, and G842 are the respective mouse IDs). Lipid labels colored in red are discussed in this paper regarding the differential lipid metabolism in PC and DC tissue (see Figure 9).

To generate a more complete picture of the colon tissue lipid profile, HILIC and RP chromatography were used for the untargeted lipidomics measurements. HILIC separation is based on the polarity of the lipid headgroup while RP separation is dependent on the hydrophobicity of the fatty acid carbon chains (number/length/saturation and unsaturation levels). MS-DIAL 4.7 was used for the comprehensive annotation and relative quantitation of polar and nonpolar lipids in 24 colon tissues of two different locations (*n* = 12 for each group consisted of 4 biological replicates and 3 biopsies per colon region). MS-DIAL is able to provide appropriate structures of lipids at species (e.g., PC 34:1), molecular species levels (e.g., PC 18:1_16:0), and *sn*-position level (e.g., PC 18:1/16:0) or full structure annotation.^29,36^ Processing of the four DDA-based untargeted lipidomics data sets obtained from RP and HILIC separation in negative and positive ionization mode indicated 1850 and 1901 unique reference-matched lipid features, respectively (Tables S14 and S15). Figure 5a and c shows PCA score plots for the untargeted lipidomics of the HILIC and RP separation. Again, a clear separation between the two colon locations could be observed. It has to be mentioned that sample G908_dc1 was lost during sample preparation and that G843_dc3 and G908_pc3 were outliers within the HILIC-measurements but clustered fine in the RP-measurements. This is also reflected in the heatmaps depicted in Figure S9.

**Figure 6:**
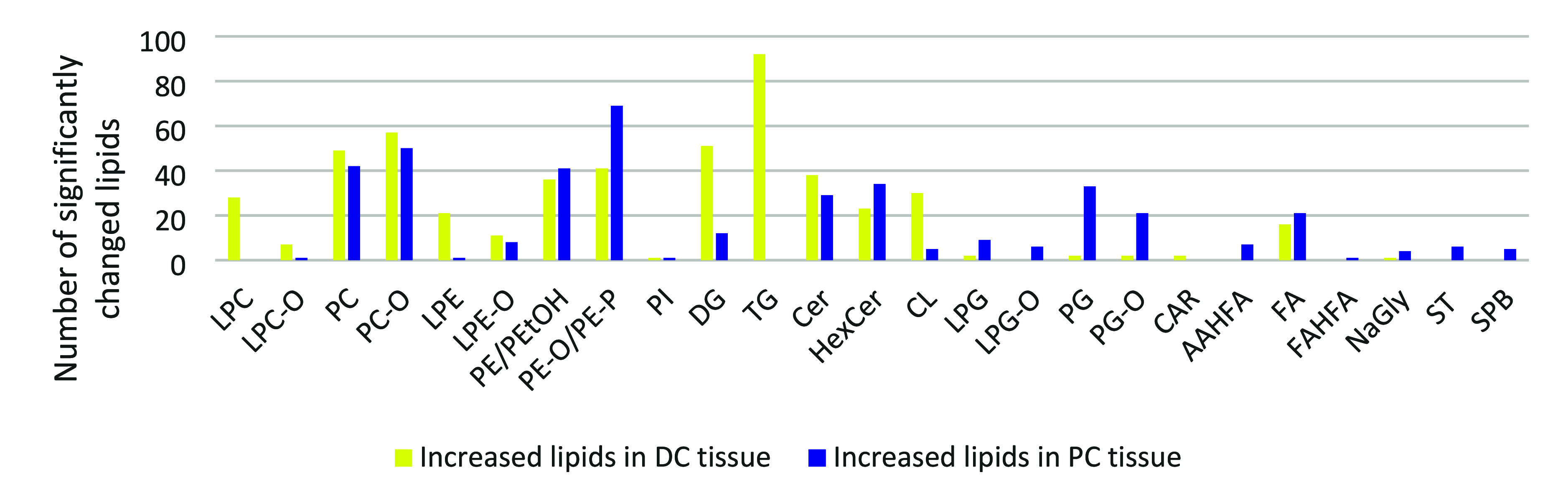
Bar chart showing significantly changed lipids classes and subclasses in DC and PC tissue based on the HILIC and RP-LC data (DC: distal colon, PC: proximal colon) (LPC, Lyso-phosphatidylcholin; LPC-O, ether-linked phosphatidylcholine; PC, phosphatidylcholin; PC-O, ether-linked phosphatidylcholine; LPE, lyso-phosphatidylethanolamine; LPE-O, ether-linked lyso-phosphatidylethanolamine; PE, phosphatidylethanolamin; PEtOH, phosphatidylethanol; PE-O, ether-linked phosphatidylethanolamine; PE-P, vinyl–ether linked phosphatidylethanolamine; PI, phosphatidylinositol; DG, diacylglycerol; TG, triacylglycerol; Cer, ceramide; HexCer, hexosylceramide; CL, cardiolipin; LPG, lyso-phosphatidylglycerol; LPG-O, ether-linked lyso-phosphatidylglycerol; PG, phosphatidylglycerol; PG-O, ether-linked phosphatidylglycerol; CAR, carnitine/acylcarnitines; AAHFA, acyl alpha-hydroxy fatty acid; FA, fatty acyl; FAHFA, fatty acid esters of hydroxy fatty acid; NaGly, N-arachidonoyl glycine; ST, sterol; and SPB, sphingoid base).

On the basis of the volcano plots of the HILIC and RP lipidomics data (Figure 5b,d), a high number of lipids (>200) with significant changes between DC and PC tissue could be observed (Table S16). As shown in Figure 6, they belong to different lipid classes and subclasses. A high number of significant TG, DG, PC, PC-O, and PE-O/PE-P are identified by the RP-LC method, while more PG, PG-O, Cer, HexCer, and CL are detected by the HILIC method. Only 87 (out of 566) up- and 97 (out of 595) down-regulated lipids are shared between the HILIC and RP lipidomics measurements (Figure S10), indicating that both untargeted lipidomics methods are needed for a comprehensive coverage of lipids with a large polarity range. Acceptable annotation of the detected lipids was confirmed based on the retention times of the Mouse SPLASH LIPIDOMIX Mass Spec Standard and similarity of the MS/MS spectra with the MS-DIAL reference database (Appendix 2 of the Supporting Information). On the basis of the HILIC and RP-LC data (Figure 6), all of the significant detected 28 LPC and 92 TG and most of the significant detected CER (38 of 67), LPC-O (7 of 8), DG (51 of 63), CL (30 of 35), and LPE (21 of 22) were increased in the DC tissue, while most PG (33 of 35), PG-O (20 of 23), LPG (9 of 11), LPG-O (6 of 6), NAGly (4 of 5), AAHFA (7 of 7), FAHFA (1 of 1), ST (6 of 6), and SPB (5 of 5) were increased in PC tissue (Table S16). A general observation was that longer chain fatty acids are present in PC, PC-O, PE, PE-O, and PE-P in the PC tissue.

#### Untargeted Proteomics of Healthy Mouse Colon Tissue

**Figure 7:**
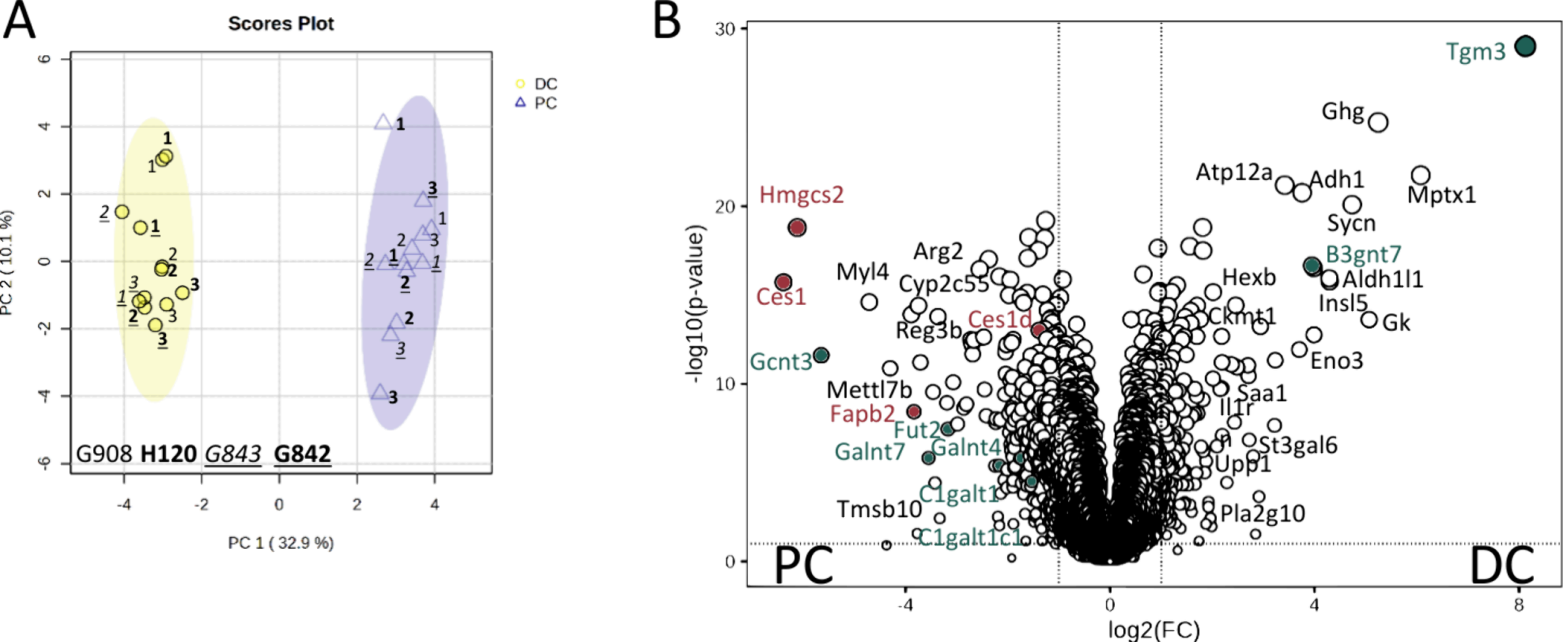
(a) PCA score plots for colon tissue untargeted proteomics and (b) volcano diagram (FC threshold: 2, FDR threshold: 0.05, direction of comparison: DC/PC) (DC: distal colon, PC: proximal colon; G908, H120, G843, and G842 are the respective mouse IDs). In red are the proteins colored which play a role in the discussed lipid metabolism and those involved in the mucin O-glycan biosynthesis are colored in green (see Figure 8 and Figure 9).

To gain more information about the enzymatic reactions involved in the metabolic and lipidomic phenotypes observed, DC and PC tissue samples were analyzed by label-free untargeted proteomics (*n* = 12 for each group consisted of 4 biological replicates and 3 biopsies per colon region). The MaxQuant output file “proteinGroups.txt”, the imputed protein expression values plus GO-term annotations are given in Tables S17, S18, S19, and S20. Figure 7a depicts the PCA of the proteomics data showing again a clear separation between the two colon tissue types. Figure 7b displays a volcano plot based on FDR < 0.05 and FC > 2. Out of the 4500 quantified proteins, 157 were significantly upregulated in the DC tissue and 263 were significantly upregulated in the PC tissue (Table S19). Figure S12 summarizes significant gene ontology terms identified within the proteomics data set specific for the PC and DC tissue (*p* adjusted < 0.05), which have an impact on the metabolome and lipidome.

Our proteomics results are in good agreement to previous publications, which studied the regional transcriptome of the colon. For example, Parigi et al. examined the spatial transcriptome of colon tissue during wound healing. They also identified Hmgcs2, Mettl7b, Fabp2, Pcp4, S100g, Cyp2c55, Muc2, Agr2, Tmsb10, and Txndc5 as highly expressed in the PC tissue and Prdx6, Tgm3, Eno3, Saa1, Atp12a, Slc37a2, Il1rn, and Csrp1 enriched in the DC tissue.^50^ Triff et al. in 2013 performed chromatin immunoprecipitations next-generation sequencing analysis (ChIP-Seq) with mRNA transcription (RNA-Seq) of the distal and proximal colon. Their RNA expression experiments also revealed an upregulation in DC tissue of Slc37a2, B3gnt7, Tgm3, and Atp12a and a downregulation of Reg3b, Reg3g, and Lgals2.^65^

### Metabo-Lipid-Prote-Omic Spatial Differences in Healthy Colon Tissue

Next, the metabolomics, lipidomics, and proteomics data sets were combined to obtain a fundamental understanding about metabolic pathways and the involved biochemical reactions. First, a joint-pathway analysis was performed using the tool MetaboAnalyst^34^ to find connections between the significant metabolites detected by both untargeted and targeted methods and the significant proteins found by untargeted proteomics. To visualize the up- and- down-regulated proteins and metabolites within the KEGG-pathway, Pathview was used.^44^ Detected pathways plus the involved metabolites and proteins are given in Table S21 and are visualized in Figures S13–S17.

**Figure 8:**
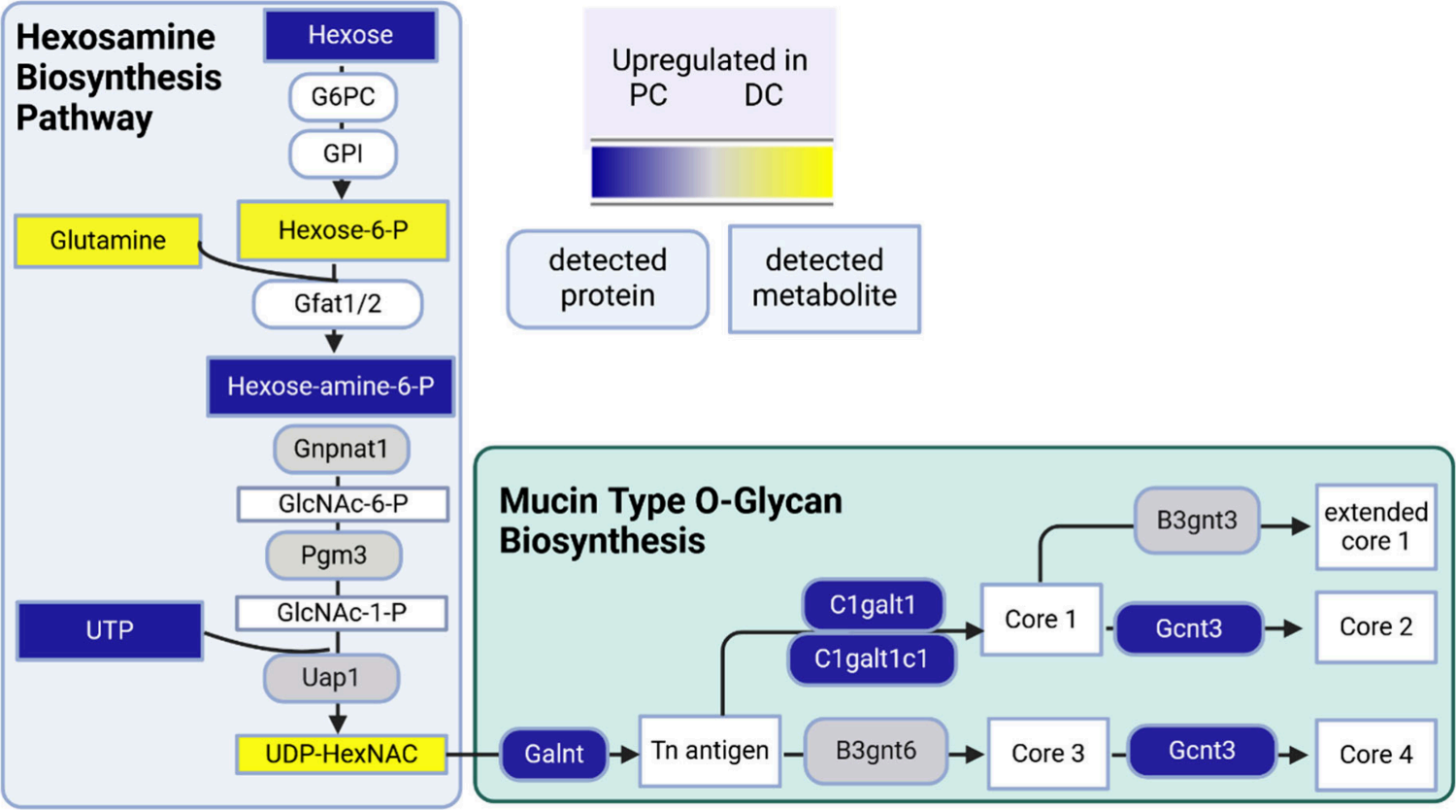
Hexosamine biosynthesis pathway and mucin-type O-glycan biosynthesis in DC and PC tissue. Proteins (squares with rounded edges) and metabolites (squares with sharp edges) upregulated in DC tissue are colored in yellow, and upregulated in PC tissue are colored in blue. The color gray indicates that the protein was detected but not significantly changed between the two tissue types.

We first looked at the mucin type O-glycan biosynthesis because of its biological function in the intestine (Figure 8). Mucins are highly glycosylated proteins, which function as lubricant of the mucosal surface, which protect the epithelial tissue against microbes and support the exchange between the luminal content and the colon epithelial.^66^ In our data set, Gcnt3 (Q5JCT0), C1galt1c1 (Q9JMG2), Galnt3 (P70419), Galnt4 (O08832), Galnt6 (Q8C7U7), Galnt7 (Q80VA0), and Galnt12 (Q8BGT9) were enriched in the PC tissue. Gcnt3 (Q5JCT0) mediates core 2 and core 4 *O*-glycan branching, two important steps in mucin-type biosynthesis. The C1galt1c1 (Q9JMG2) is also involved in *O*-linked glycosylation of mucins by generating precursors for many extended *O*-glycans in the glycoproteins. High-levels of UDP-GalNAc were observed in the DC tissue, indicating that this precursor was consumed by the O-glycan forming proteins in the PC tissue. In addition, the GO-enrichment analysis yielded the GO-term GO:0004653_F: polypeptide N-acetylgalactosamyltransferase which contained Galnt4, as well as Galnt3, Galnt7, and Galnt12 (Figure 8, Table S20, and Figure S13). These proteins catalyze the initial transfer of an N-acetyl-d-galactosamine to a serine or threonine residue and have activity toward EA2, Muc1a, Muc1b, Muc2, Muc5, Muc7, and EPO-T which are proteins partially involved in mucin formation. These proteins are increased in the PC tissue, indicating an effect on mucin-biosynthesis. Furthermore, Fut2 (Q9JL27), a galactoside alpha-(1,2)-fucosyltransferase, and B3galt5, a beta-1,2-galatosyltransferase, were also enriched in the PC tissue. Whereas B3gnt7 (Q8K0J2), a UDP-GlcNac:betaGal beta-1,2-N-acetylglucosaminyltransferase, and St3gal6 (Q8VIB3), a type 2 lactosamine alpha-2,3-sialyltransferase, were significantly increased in the DC tissue. Because of these findings, we had a deeper look at which glycosyltransferases were present in the colon and were enriched in a specific region. We searched for all glycosyltransferases reported by Taniguchi et al.^67^Table 1 depicts the list of all detected glycosyltranferases in the colon tissue within our data set and their enrichment within the DC and PC tissue. This data indicates that the highly glycosylated mucins are regionally modified within the colon. Next to the glycosyltransferase enzymes, we observed another protein involved in mucin stabilization: Tgm3 (Q08189), a protein-glutamine gamma-glutamyltransferase E, which was strongly expressed in the DC tissue and has an important cross-linking activity to stabilize the colonic mucus gel network.^68^ In our study as well as in Parigi et al., the Tgm3 expression was the highest in the distal colon indicating toward a spatial role of Tgm3 in the intestine.^50^ Most likely, differences in mucus producing goblet cells, the presence of different cell types, and different bacteria within the different regions of the colon might be responsible for the observed differences.^46,69^

**Table 1:** Detected Mouse Glycosyltransferase Proteins in the Colon Tissue

Enzymatic activity	Proteins detected but not significantly regulated	Significantly increased in PC tissue	Significantly increased in DC tissue
ALG			
Dol-P-Man Synthesis	Dpm1		
	Dpm3		
Cytolasmic ALG (ManT)	Alg2		
Fucosyltransferase			
Fucosyltransferase		Fut2	
O-Fucosyltranserase	Pofut1		
	Pofut2		
Galactosyltransferase			
Beta 1,3		B3galt5	
		C1galt1	
Beta 1,4	B4galt1		
N-Acetylgalactosaminyl-transferase			
UDP-GalNAc:polypeptide N-acetylgalactosaminyltransferases	Galnt5	Galnt3	
	Galnt10	Galnt4	
		Galnt6	
		Galnt7	
		Galnt12	
N-Acetylglucosaminyl-transferase			
Alpha 1,4	A4gnt		
Beta 1,3	B3gnt3		B3gnt7
	B3gnt6		
Beta 1,6 (core2)		Gcnt3	
MGAT family	Mgat4a	Mgat3	
	Mgat4c		
O-linked	Ogt		
Sialyltransferase			
Beta-Gal alpha 2,3-sialyltransferase	St3gal4		St3gal6
Beta-GalNAC alph 2,6-sialyltransferase	St6galnac6		
Sulfotransferase			
HNK	Chst4		
Gal 3-O-sulfotransferase			Gal3st2
Xylosyltranferase	Gxylt1		
Others		C1galt1c1	

Next, we looked how the substrate UDP-GalNAC was produced. In literature, the formation of UDP-GalNAC is reported via the hexosamine biosynthesis pathway. First, glucose is conjugated with phosphate to form glucose-6-phosphate and subsequently fructose-6-phosphate. Since our untargeted screening could not differentiate between sugar moieties and phosphate positions, we summarized this step in Figure 8 as hexose-6-P. The latter is then converted via the proteins Gnpnat1 (Q9JK38), Pgm3 (Q9CYR6), and Uap1 (Q91YN5) to UDP-GalNAC which then modifies the mucin core proteins. Uap1 catalyzes the reaction from UTP and galactosamine-1-phosphate to UDP-GalNAc. In summary, mucin production and mucin-type O-glycan biosynthesis demonstrate regional differences based on enzyme expression and availability of the substrate in the colon which is summarized in Figure 8.

The challenges of this metabo-lipid-prote-omics data set were to connect the three Omics-disciplines to obtain a deeper understanding of the differences between PC and DC tissue. When we mapped the data to KEGG biosynthetic pathways often only a few metabolites and proteins matched. For example, “Arginine biosynthesis” was the most significant pathway within the joint pathway analysis (Figure S14 and Table S21). High levels of Orn and Cit (AA) in the PC tissue correlated well with the high expression of Otc (P11725, 2.1.3.3), Cps1 (Q8C196, 6.3.4.16), and Arg2 (O08691, 3.5.3.1) in that tissue. The same hold true for Glul (P15105, 6.3.1.2) expression in DC tissue and higher levels of Gln in the respective tissue. In the “Arginine and proline metabolism” pathway (Figure S15), we observed high levels of creatine and creatinine in the DC tissue which correlates with the high expression of Ckmt1 (P30275, 2.7.3.2) in that respective tissue. The creatine-phosphocreatine cycle plays an essential role in the energy distribution in cells. Its role has recently been discussed in murine colitis.^70^

Other altered KEGG-pathways were the “Pyrimidine metabolism” and “Purine metabolism” (Figure S16 and Figure S17). The pyrimidine pathway affects nucleic acids and phospholipids synthesis, glucose metabolism, and protein glycosylation.^71^ In our case, several nucleotides like CTP, CDP, Cyd, Thd, UTP, UDP, and Urd were upregulated in PC tissue. For example, an intracellular CTP pool is essential for the phospholipid biosynthesis^71^ and might be related to the altered phospholipid metabolism of the DC and PC colon. Furthermore, a strong protein expression of Upp1, a uridine phosphorylase (P52624, 2.4.2.3), was observed for the DC tissue and a strong protein expression for Dpyd, a dihydropyrimidine dehydrogenase (Q8CHR6, 1.3.1.2), and Cda, a cytidine deaminase (P56389, 3.5.4.5), in the PC tissue. In addition, the GO-term GO:0015020_F:glucuronosyltransferase activity was increased in PC tissue which represents 5 UDP-glucuronosyltransferases [Ugt2b17 (P17717), Ugt1a1 (Q63886), Ugt1a6 (Q64435), Ugt1a7c (Q6ZQM8), and Ugt2a3 (Q8BWQ1, 2.4.1.17)]. Higher levels of the substrate UDP-glucuronide and its precursor UDP-glucose were observed in the DC tissue, indicating the usage of the UDP-glucuronide pool in PC tissue. In line with that, the protein expression of UDP-glucose-6-dehydrogenase (UGDH, O70475, 1.1.1.22) was increased in the PC tissue. This enzyme catalyzes the reaction from UDP-glucose to UDP-glucuronic acid. UTP is also involved in the formation of UDP-N-acetylglucosamine (UDP-GlcNAc) which is the substrate for O-linked glycosylation reactions of proteins (see Table 1).^71^ In summary, each single visualization of a KEGG biosynthetic pathways only depicts a small amount of relevant spatial reactions in the mouse colon. To understand the impact of these reactions on the characteristics of PC and DC tissue still requires manual work and a profound literature search.

Another limitation is that lipids are underrepresented in the KEGG-pathways. To identify pathways related to the lipid metabolism and the respective proteins involved, the BioPAN tool of LIPIDMAPS was applied to the untargeted lipidomics data.^43^ 1271 lipids (out of 2341) of HILIC- and RP-based untargeted lipidomics data set were recognized by BioPAN and processed. Pathway names based on lipid subclasses, Z-score for involved reactions, as well as the genes predicted for each pathway are given in Tables S22 and S23. Z-score considers the mean and the standard deviation of the experiment assuming a normally distributed data of lipid subclasses. Reactions are categorized into two activated or suppressed groups based on the direction of change, and significant ones are determined at a *p*-value < 0.05, which corresponds to a *Z*-score > 1.645.^43^ The results of the BioPAN analysis are depicted in Figures S18 and S19.

The most significant active pathways in PC tissue were the biosynthesis of sphingosine-1-phosphate (Cer→SPB) and the biosynthesis of sphingomyelin (Cer→SM), in which according to BioPAN, Acer1, Acer2, Acer3, Asah1, Asah2, Asah2B, Sgms1, Sgms2, and Cert1 proteins were involved. Asah1 (Q9WV54) was also found in the total list of the detected proteins in healthy mouse colon tissues but was not differentially regulated between the two tissue types. Additionally, the ORA analysis demonstrated increased levels in the DC tissue of Smpd2 (O70572) and Smpd3 (Q9JJY3). These are are sphingomyelin phosphodiesterases hydrolyzing sphingomyelin to form ceramides which could explain the differences in sphingolipids (GO-term GO:0004620_F:phospholipase activity). The most significant suppressed pathways in PC tissue were the triglycerol metabolism (MG → DG → TG), in which Mgat and Dgat2 proteins are involved according to BioPAN. These two proteins were not detected within our proteomics data set. Significant enriched levels of TGs were detected in the DC tissue, which correlates to the suppression of the triglycerol metabolism in PC tissue and might also explain the higher levels of acylcarnitines in the DC tissue. The GO-term GO:0004806_F:triglyceride lipase activity summarizes several carboxylesterases [Ces1e (Q64176), Ces2e (Q8BK48), Ces1 (Q8VCC2), Ces1d (Q8VCT4), and Ces1f (Q91WU0)] which were increased in the PC tissue which also might explain the reduced levels of TG in the PC tissue.^72^

**Figure 9:**
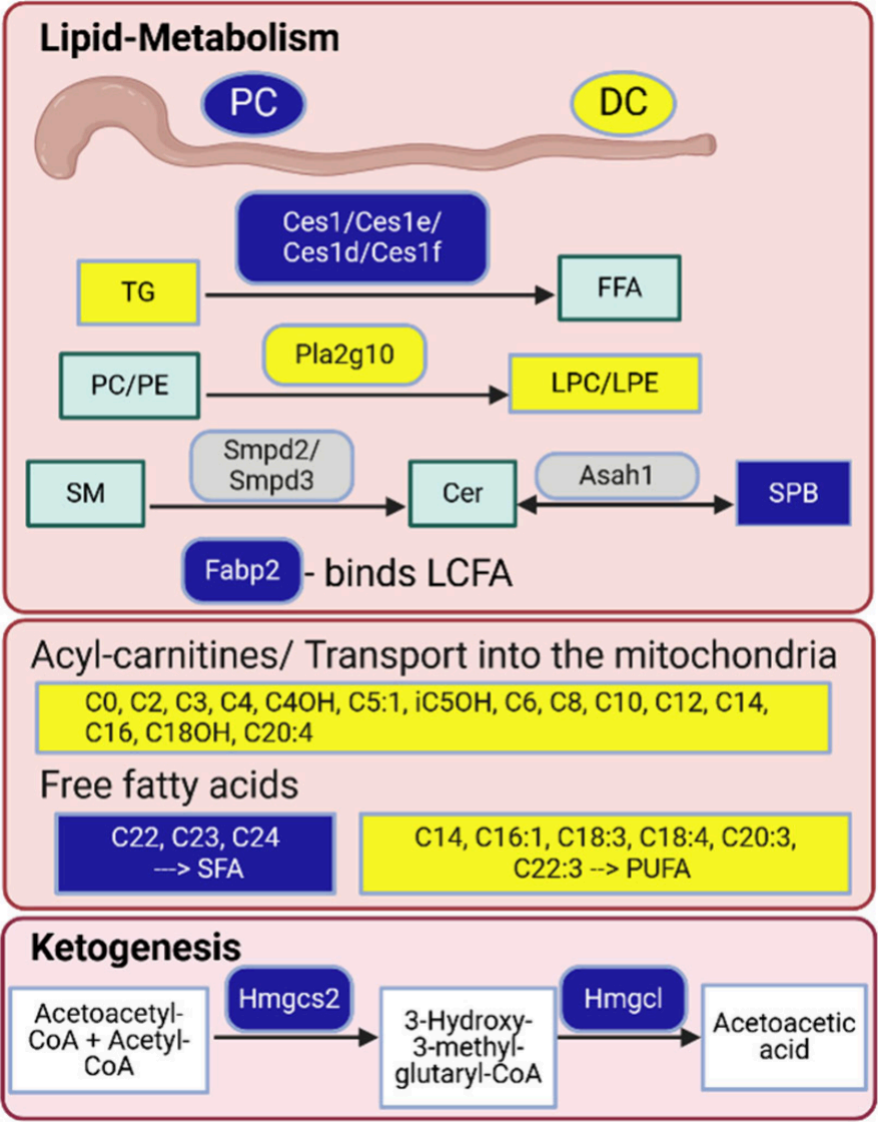
Summary of the differences in lipid metabolism between PC and DC tissue. Proteins (squares with rounded edges) and lipids (squares with sharp edges) upregulated in DC tissue are colored in yellow and upregulated in PC tissue are colored blue. The color gray indicates detected but not significantly changed. Lipid main classes whose lipids are significantly increased in DC or PC tissue are colored in green.

The FFA active and suppressed pathway in PC tissue is shown in Figure S19. High levels of long chain saturated fatty acids (22:0, 23:0, and 24:0) were detected in PC tissue. Elovl1 and Elovl5 are the proteins suggested in the elongation of the very long chain fatty acids. These two proteins were not detected within our proteomic data set. Instead, Acsl1 (P41216), Acsl3 (Q9CZW4), and Acsl5 (Q8JZR0), which are long-chain-fatty-acids-CoA ligases, are present but not significantly changed in their expression levels between PC and DC tissue. Eventually, Fabp2 (P55050), a fatty acid-binding protein, which can bind saturated long-chain fatty acids with high affinity, is involved in the high levels of very long chain fatty acids because it was significantly increased in the PC tissue. Long-chain fatty acids are nutrient sources, function as signaling molecules, and modulate the virulence of enteric pathogens.^73^ Baxter et al. quantified metabolites and lipids from human biopsies of the ascending and descending colon from normal weight, overweight, and obese patients, which also demonstrated differences in long chain fatty acid as well as lipid metabolism.^74^ Unfortunately, their panel did not contain the ultralong chain fatty acids, lignoceric acid, behenic acid and tricosanoic acid, which were increased in the mouse PC tissue. Next, the BioPAN data suggest that Pla2g2e, Pla2g2a, Pla2g2d, Pla2g2f, Pla2g1b, Pla2g4a, Pla2g4b, Pla2g4c, Pla2g4d, Pla2g4e, and Pla2g4f genes are involved in the formation of LPC out of PC. Within our data set, we could only detect Pla2g4a (P47713), Pla2g16 (Q8R3U1), Pla2g15 (Q8VEB4), and Pla2g10 (Q9QXX3). Only the latter one was significantly enriched in the DC tissue. This protein hydrolyzes the ester bond of a fatty acid group attached at sthe n-2 position of phospholipids with preference for PC and PG over PE. This might explain the higher levels of LPC and LPE in the DC tissue. The GO-term GO:0019915_P:lipid storage with the enriched proteins Hexb, Hexa, Cd36, and Gm2a might also have an impact on the detected lipid levels. In general, our data suggest a different energy supply of the two tissue types. Several ACs, FFAs, and TGs were significantly enriched in the DC tissue, whereas Hmgcs2 (P54869) is strongly expressed in the PC tissue. The latter catalyzes the first reaction of ketogenesis, a metabolic pathway that provides lipid-derived energy under carbohydrate deprivation (Figure 9). In summary, BioPAN and ORA are excellent tools for the lipid pathway analysis to connect lipids to proteins. The lipid metabolism of the PC and DC tissue is summarized in Figure 9. Unfortunately, within our data set most of the suggested proteins of the BioPAN-tool were neither differentially regulated nor detected.

## Summary and Conclusion

The newly developed mass spectrometry-based workflow enables for the first time the absolute and relative quantitation of metabolites, lipids, and proteins from a single tissue biopsy. The analysis of distal and proximal colon mouse tissue demonstrated spatial differences on metabolite, lipid, and protein levels. Multiomics analysis tools revealed regional differences, among others, in mucin modifications and lipid metabolism. The detected differences in healthy colon might derive from the regional differences in cell types, the disparate blood supply, lymphatic drainage, as well as the altered development of the mid- and hindgut and different microbial and metabolite impact on the respective colon region. Our study stresses the importance of taking spatial differences of the colonic epithelium into account when studying colonic diseases such as IBD or CRC in mice.

## Supporting Information Available

(Table S1) Standards and stable isotopically labeled-internal standards; (Table S2) sample features; (Tables S3–S7) MRM conditions for measuring metabolites, FFAs, and Mouse SPLASH LIPIDOMIX Mass Spec Standard; (Table S8) recovery efficiency, matrix effect, and process efficiency for SIL-ISs; (Table S9) external calibration curves and figures of merit; (Table S10) precision and accuracy of the QC samples; (Table S11) targeted colon tissue metabolomics and FFAs data; (Table S12) untargeted colon tissue metabolomics; (Table S13) significantly changed metabolites in the colon tissue; (Table S14) untargeted colon tissue lipidomics obtained by HILIC method; (Table S15) untargeted colon tissue lipidomics obtained by RP method; (Table S16) volcano plot data of the HILIC and RP lipidomics data; (Table S17) MaxQuant output file; (Table S18) imputed protein expression values; (Table S19) significantly changed proteins and genes; (Table S20) significant protein pathways in the colon tissue; (Table S21) joint pathways analysis results; (Table S22) lipid class and FFA pathways in the colon tissue (BioPAN results); and (Table S23) lipid class and FFA reactions (BioPAN results) (XLSX)
Supporting Information Text File contains additional information on Material and Methods (page 4), the Targeted Metabolomics Methods (5), and on supporting figures: (Figure S1) QC chromatograms obtained by HILIC based untargeted metabolomics; (Figure S2) QC chromatograms obtained by HILIC-based untargeted lipidomics; (Figure S3) QC chromatograms obtained by RP-based untargeted lipidomics; (Figure S4) identified/quantified protein groups for the extraction conditions with or without ammonium salt; (Figure S5) QC chromatograms obtained by UHPLC–MS/MS for amino acids, acylcarnitines, short chain fatty acids and organic acids, nucleotides, and free fatty acids; (Figure S6) heatmap for targeted metabolomics of the colon tissue; (Figure S7) heatmap for HILIC-based colon tissue untargeted metabolomics; (Figure S8) scatter plot of the ratios PC and DC of the targeted and untargeted metabolomics data; (Figure S9) heatmaps for HILIC and RP-LC-based colon tissue lipidomics; (Figure S10) Venn diagrams for significantly up/down-regulated lipids in DC tissue based on both HILIC and RP-LC-based colon tissue lipidomics; (Figure S11) pie charts showing percentages of significantly changed lipid classes and subclasses detected by HILIC and RP-LC methods; (Figure S12) significant gene ontology terms; (Figure S13) mucin-type O-glycan biosynthesis; (Figure S14) arginine biosynthesis; (Figure S15) arginine and proline metabolism; (Figure S16) pyrimidine metabolism; (Figure S17) purine metabolism; (Figure S18) BioPAN lipid subclasses networks and reactions for PC tissue versus DC tissue; (Figure S19) BioPAN networks based on fatty acid for PC tissue versus DC tissue based on active and suppressed pathways; (Appendix I) MS/MS annotation for metabolites by using MS DIAL; (Appendix II) MS/MS annotation for lipids by using MS DIAL (PDF)

## Notes

All proteomics raw files as well as the MaxQuant output files have been deposited to the ProteomeXchange Consortium via the PRIDE partner repository and can be accessed using the identifier PXD046811 (https://proteomecentral.proteomexchange.org/cgi/GetDataset?ID=PXD046811). The targeted and untargeted metabolomics and lipidomics data has been deposited at MASSIVE (https://massive.ucsd.edu) under the number MSV000093241. Figures were created with BioRender.com.

## Abbreviations

AA: amino acids
AAHFA: acyl alpha-hydroxy fatty acid
AC: acylcarnitine
ACN: acetonitrile
AcOH: acetic acid
Ans: anserine
BA: bile acid
BioPAN: bioinformatics Methodology for Pathway Analysis
BSA: bovine serum albumin
BCA: bicinchoninic Acid
CL: cardiolipin
CA: cholic acid
CAA: two chloroacetamide
Car: carnitine
Cer: ceramides, Cho, choline
creat: craetinine
CRC: colorectal cancer
CV: coefficient of variation
DCM: dichloromethane
DCA: deoxycholic acid
DG: diacylglycerol
GlcN-6-P: d-glucosamine 6-phosphate
DDA: data-dependent acquisition
DTT: dithiothreitol
DC tissue: distal colon tissue
EDC: *N*-(3-(dimethylamino)-propyl)-*N*′-ethylcarbodiimide hydrochloride
EDTA: ethylenediaminetetraacetic acid
Ex: extraction
FA: formic acid
FA (2): fatty acyl
FAHFA: fatty acid esters of hydroxy fatty acid
FFA: free fatty acids
FC: fold change
FDR: false discovery rate
GDP-glucose: guanosine diphosphate-glucose
GDP-mannose: guanosine diphosphate mannose
GDP-L-fucose: guanosine diphosphate fucose
HCD: higher energy collision dissociation
HILIC: hydrophilic interaction liquid chromatography
HR-MS: high resolution-mass spectrometry
iBAQ: intensity-based absolute quantification
inflammatory bowel diseases: IBD
IPA: isopropanol
LDA: lipid data analyzer
LFQ: label-free quantitation
Lyso-PL: lysophospholipid
LOQ: limit of quantification
LPC: lysophosphatidylcholine
LPC-O: ether-linked lysophosphatidylcholine
LPE: lysophosphatidylethanolamine
LPE-O: ether-linked lysophosphatidylethanolamine
LPG: lysophosphatidylglycerol
LPG-O: ether-linked lysophosphatidylglycerol
LC-PUFA: long-chain polyunsaturated fatty acid
LCFA: long-chain fatty acids
M6P: D-mannose 6-phosphate
MeOH: methanol
MG: monoacylglycerol, MS, mass spectrometry
MRM: multiple reaction monitoring
MTBE: methyl *tert*-butyl ether
NaGly: N-arachidonoyl glycine
NAG: N-acetyl-l-glutamate
3-NPH: 3-nitrophenylhydrazine hydrochloride
NUC: nucleotides
AdoMet: S-adenosyl-l-methionine
OA: organic acid
ORA: over-representation analysis
PA: phosphatidic acid
PC: phosphatidylcholine
PC-O: ether-linked phosphatidylcholine
PE: phosphatidylethanolamine
PE-O: ether-linked phosphatidylethanolamine
PG: phosphatidylglycerol
PG-O: ether-linked phosphatidylglycerol
PI: phosphatidylinositol
PS: phosphatidylserine
PCA: principal component analysis
PLS-DA: partial least-squares discriminant analysis
PSMs: peptide-to-spectrum matches
PBS: phosphate-buffered saline
PC tissue: proximal colon tissue
PL: phospholipid
QC: quality control
QEA: quantitative enrichment analysis
QTOF: quadrupole-time-of-flight
Re-Ex: re-extraction
RP-LC: reverse-phase liquid chromatography
SCFA: short chain fatty acids
SIL-IS: stable isotopically labeled-internal standard
ST: sterol
SD: standard deviation
Spd: spermidine
Spm: spermine
TIC: total ion current normalization
TCA cycle: tricarboxylic acid cycle
TG: triacylglycerol
UDP-GlcNAc: uridine diphosphate N-acetylglucosamine
UDP-GalNAc: uridine diphosphate-N-acetyl-d-galactosamine
UDP-D-xylose: uridine diphosphate-D-xylose (for additional abbreviations of chemicals, see Table S1).
