# Supplementary material for: Development of a Global Metabo-Lipid-Prote-omics Workflow to Compare Healthy Proximal and Distal Colonic Epithelium in Mice
Source: J Proteome Res. 2024 Jul 25;23(8):3124–40. doi: 10.1021/acs.jproteome.3c00771 (PMC11301693; doi:10.1021/acs.jproteome.3c00771)
Supplement: Supplementary file 2 — pr3c00771_si_002.pdf [file pr3c00771_si_002.pdf]

Supporting information

Development of a global metabo-lipid-prote-omics workflow to compare healthy proximal and distal colonic epithelium in mice

Maryam Hemmati [1], Susanne I. Wudy [1], Franziska Hackbarth [1], Verena K. Mittermeier-Kleßinger [2], Olivia I. Coleman [4], Dirk Haller [4,5], Christina Ludwig [1], Corinna Dawid [1,2,3,5], Karin Kleigrewe [1]*

[1] Bavarian Center for Biomolecular Mass Spectrometry, TUM School of Life Sciences, Technical University of Munich, 85354 Freising, Germany
[2] Chair of Food Chemistry and Molecular Sensory Science, TUM School of Life Sciences, Technical University of Munich, 85354 Freising, Gernany
[3] Professorship for Functional Phytometabolomics, TUM School of Life Sciences, Technical University of Munich, 85354 Freising, Germany
[4] Chair of Nutrition and Immunology, TUM School of Life Sciences, Technical University of Munich, 85354 Freising, Germany
[5] ZIEL Institute for Food and Health, Technical University of Munich, 85354 Freising, Germany[1, 2]
*(*karin.kleigrewe@tum.de)*

Table of Content

Supplemental-Information Tables……………………………………………………………………see Excel Sheet

Material and Methods……………………………………………………………………………………S5

Targeted metabolomics – UPLC settings………………………………………………………………S6

Supplemental Figures……………………………………………………………………………………S8

References…………………………………………………………………………………………………S27

Appendix…………………………………………………………………………………………………S27

Table of Supplemental Tables (extra Excel-Sheet)

Table S1.Standards and stable isotopically labelled-internal standards with more information about their abbriviations and suppliers.
Table S2. Sample features.
Table S3. MRM conditions for measuring amino acids and acyl carnitines.
Table S4. MRM conditions for measuring nucleotides.
Table S5. MRM conditions for measuring short chain fatty acids and organic acids.
Table S6. MRM conditions for measuring free fatty acids.
Table S7. MRM conditions for measuring Mouse SPLASH® LIPIDOMIX® Mass Spec Standard.
Table S8. Recovery efficiency (RE%), matrix effect (ME%), and process efficiency (PE%) for SIL-ISs of metabolites and lipids.
Table S9. External calibration curves and validation parameters.
Table S10. Precision and accuracy of the QC samples.
Table S11. Targeted metabolomics and FFAs data normalized as nmol/g tissue.
Table S12. Alignment file normalized based on TIC, obtained by MS-Dial, for HILIC based untargeted colon tissue metabolomics method in both negative and positive modes.
Table S13. Significantly-changed metabolites in DC and PC tissues.
Table S14. Alignment file normalized with SPLASH® LIPIDOMIX® Standard, obtained by MS-Dial, for HILIC based untargeted lipidomics method in both negative and positive modes.
Table S15. Alignment file normalized with SPLASH® LIPIDOMIX® Standard, obtained by MS-Dial, for RP based untargeted lipidomics method in both negative and positive modes.
Table S16. Volcano plot data of the HILIC and RP lipidomics data.
Table S17. MaxQuant output file.
Table S18. Imputed log10 protein expression values, log fdr p values and mean difference for PC and DC tissues
Table S19.Significantly-changed proteins and genes in DC (row 2) and PC (row 178).
Table S20. Significant protein pathways in DC and PC tissues (DC (row 2) and PC (row 229) refer to distal colon and proximal colon, respectively).
Table S21. Joint pathways results.
Table S22. Lipid class and FFA pathways (active and suppressed) for PC versus DC tissues as well as predicted genes, based on untargeted data.
Table S23. Lipid class and FFA reactions (active and suppressed) for PC versus DC tissues as well as predicted genes, based on untargeted data.

Table of Supplemental Figures

Figure S1 (a and b). QC chromatograms obtained by HILIC based untargeted metabolomics method in negative and positive modes, respectively..8
Figure S2 (a and b). QC chromatograms obtained by HILIC based untargeted lipidomics method in negative and positive modes, respectively...9
Figure S3 (a and b). QC chromatograms obtained by RP based untargeted lipidomics method in negative and positive modes, respectively...10
Figure S4 Identified/quantified protein groups for the extraction conditions with or without ammonium salt (A: MTBE/MeOH/H_2O (10:3:2.5, v/v/v), B:MTBE/MeOH/Ammonium salt solution (0.3M) (10:3:2.5, v/v/v), C: MTBE/MeOH/H_2O (7:3:2.5, v/v/v), D: MTBE/MeOH/ Ammonium salt solution (0.3M) (7:3:2.5, v/v/v)). 11
Figure S5: (a, b, c, d, and e). QC Chromatograms obtained by UHPLC-MS/MS for amino acids, acyl carnitines, short chain fatty acids and organic acids, nucleotides, and free fatty acids, respectively. 12
Figure S6: Heatmap (Distance measure: Euclidean, Cluster algorithm: Ward) for targeted metabolomics of the colon tissue (DC: distal colon, PC: proximal colon). 13
Figure S7 Heatmap (Distance measure: Euclidean, Cluster algorithm: Ward) for HILIC based colon tissue untargeted metabolomics (DC: distal colon, PC: proximal colon). 14
Figure S8 Scatter plot of the ratios PC and DC of the targeted and untargeted metabolomics data of each single biopsy sample of each respective metabolite. Only metabolites, which were present and correctly annotated in the targeted and untargeted datasets, were used. The correlation coefficient is 0.63. 15
Figure S9 Heatmaps (Distance measure: Euclidean, Cluster algorithm: Ward) for a) HILIC and b) RP-LC based colon tissue lipidomics, respectively (DC: distal colon, PC: proximal colon). 16
Figure S10 (a and b) Venn diagrams for significantly up/down-regulated lipids in DC tissue based on both HILIC and RP-LC based colon tissue lipidomics (DC: distal colon, PC: proximal colon). 17
Figure S11 Pie charts showing percentages of significantly changed lipid classes and subclasses detected by HILIC and RP-LC methods, respectively. 17
Figure S12 Significant gene ontology terms specific for the PC (bottom) and DC (top) tissue (-$\log_{10}$(FDR)). The black shaded area present the percentage of the size overlap and the gene set. 18
Figure S13 Mucin type O-glycan biosynthesis based on KEGG. Increased metabolite and protein concentrations in the PC tissue are colored in blue and green and increased metabolite and protein concentrations in DC tissue in yellow and red. The figure was created with Pathview.[10] 19
Figure S14 Arginine Biosynthesis based on KEGG. Increased metabolite and protein concentrations in the PC tissue are colored in blue and green and increased metabolite and protein concentrations in DC tissue in yellow and red. The figure was created with Pathview.[10] 20
Figure S15 Arginine and proline metabolism based on KEGG. Increased metabolite and protein concentrations in the PC tissue are colored in blue and green and increased metabolite and protein concentrations in DC tissue in yellow and red. The figure was created with Pathview.[10] 21
Figure S16 Pyrimidine metabolism based on KEGG. Increased metabolite and protein concentrations in the PC tissue are colored in blue and green and increased metabolite and protein concentrations in DC tissue in yellow and red. The figure was created with Pathview.[10] 22
Figure S17 Purine metabolism based on KEGG. Increased metabolite and protein concentrations in the PC tissue are colored in blue and green and increased metabolite and protein concentrations in DC tissue in yellow and red. The figure was created with Pathview.[10] 23
Figure S18 BioPAN lipid sub-classes networks and reactions for PC tissue versus DC tissue based on an active status, (obtained from the merged data set of HILIC and RP-based untargeted lipidomics data) (White nodes: no status, Green nodes: active status, Green arrows: reactions with a positive Z

score, Purple arrows: reactions with a negative Z score, Green shaded arrows (for both green and purple arrows): Significant reactions based on p value < 0.05 (Z-score >1.645), no paired data). 24
Figure S19 (a and b) BioPAN networks based on fatty acid for PC tissue versus DC tissue based on active and suppressed pathways, respectively (both HILIC and RP-based untargeted lipidomics data), (White nodes: no status, Green nodes: active/suppressed status, Green arrows: reactions with a positive Z score, Purple arrows: reactions with a negative Z score, Green shaded arrows (for both green and purple arrows): Z-score (positive or negative) implied in an active/suppressed status, p value < 0.05, and no paired data). 25

APPENDIX

Appendix 1 MS/MS annotation for metabolites by using MS DIAL. 28
Appendix 2 MS/MS annotation for lipids by using MS DIAL. 49

Material and Methods

Stock and working solutions

Stock solutions of 37 AAs in the range from 1.1 mM to 96.5 mM in H_2O:ACN (9:1, v/v) (except cysteine (in H_2O)), 22 ACs in the range from 1.1 mM to 4.3 mM in MeOH, 33 NUCs in the range from 0.2 µM to 15.6 mM in H_2O/MeOH (9:1, v/v) (except GMP, GDP, GTP, Ade, UTP, Xao, Ura, Urd, 2′,3′-cGAMP, 3′,2′-cGAMP, and c-di-GMP (all in H_2O); Guo and Xan (in DMSO); and Gua and Hyp (in 25% FA)), 15 SCFAs in the range from 27.5 mM to 469.2 mM in ACN/H_2O (5:5, v/v), 9 OAs in the range from 6.8 mM to 171 mM in H_2O:MeOH (8:2, v/v), and 29 FFAs in the range from 0.3 mM to 39.3 mM in MeOH were prepared. Next, high-concentrated mix solutions for each group of metabolites (250 µM for 37 AAs plus carnitine, 100 µM for 21 ACs, concentration range of 0.02 µM to 200 µM for 33 NUC, 1000 µM for 24 SCFAs and OAs, and 60 µM for 29 FFAs (except C22:5, 30 µM)) were prepared. These solutions were then diluted for the preparation of calibration curves, as described in the next part. Stock solutions of 27 SIL-IS-AAs in the range from 2.1 mM to 126 mM in H2O:ACN (9:1, v/v), 10 SIL-IS-ACs in the range from 2.1 mM to 8.6 mM in MeOH, 11 SIL-IS -non-phosphorylated-NUC in the range from 0.5 mM to 6.4 mM in H_2O:MeOH (8:2, v/v) (except IS Guo and IS Xan in DMSO; IS Gua, IS Hyp, and IS Thy in 5% FA:MeOH (8:2, v/v), 11 SIL-IS-SCFAs in the range from 6.8 mM to 29 mM in ACN:H2O (5:5, v/v), 7 SIL-IS-OAs in the range from 1.6 mM to 21.4 mM in H2O:MeOH (8:2, v/v), and 12 SIL-IS-FFAs in the range from 0.2 to 10.1 mM in MeOH were prepared. Then, a SIL-IS mix solution for each group was obtained with the concentration ranges as follows: 100 µM to 500 µM for the 27 SIL-IS-AAs plus IS carnitine, 10 µM to 100 µM for 9 SIL-IS-ACs, 100 µM for the SIL-IS-phosphorylated-NUCs, 80 µM to 200 µM for the SIL-IS-non-phosphorylated NUCs, 150 µM for the 11 SIL-IS-SCFAs, 100 µM for the of 7 SIL-IS-OAs, and 40 µM for the 12 SIL-IS-FFAs.

Calibration curves and QC samples

To quantify metabolite in the colon tissues, calibration curves were constructed with a set of calibrator levels (15 levels from 0.005 µM to 200 µM for 37 AAs and carnitine, 15 levels from 25 nM to 15 µM for 21 ACs, 11 levels for 33 NUCs from 0.125 µM to 150 µM for 4 triphosphorylated-NUCs, 0.05 µM to 75 µM for 6 diphosphorylated-NUCs, 0.025 µM to 30 µM for 9 monophosphorylated-NUCs, 0.03 µM to 45 µM for Ado, Cyt, and Cyd, and 0.01µM to 15 µM for 11 rest compounds), and 11 levels from 125 nM to 5 µM for 29 FFAs. One zero calibrator level (blank plus IS) for each group of metabolites was also prepared. SIL-IS concentrations were kept at the constant level for all calibrator levels of each group of metabolite (10 µM to 50 µM for 27 SIL-IS-AAs and IS carnitine, 0.1 µM to 1 µM for 9 SIL-IS-ACs, 20 µM for 5 SIL-IS-phosphorylated-NUCs, 2 µM to 5µM for 11 SIL-IS-non-phosphorylated-NUCs, and 1.25 µM for 12 SIL-IS-FFAs). SCFA and OA calibration curve solutions were prepared at 12 levels from 0.25 µM to 1000 µM. Then, the extra step of derivatization with 3-NPH was perfomred. [3, 4] To do this, 30 µL of each calibrator level combined with 10 µL of SIL-IS mix solution of 12 SCFAs and 7 OAs (50 µM of the SIL-IS-OAs solution and 150 µM of the SIL-IS-SCFAs solution) was mixed with 10 µL 3-NPH and 10 µL EDC, followed by incubation at 40 °C for 30 min, and finally 0.44 mL H_2O/ACN (5:5,v/v) was added. All calibration curves samples were prepared freshly and measured by using LC-MS-based targeted methods**.** The middle calibrator level of each group of metabolite was selected as QC sample for targeted analysis, which was analyzed at the beginning of the targeted-analytical run and regularly at every 8 injections throughout the batch. The samples were measured in a randomized manner.

For untargeted metabolomics, QC was prepared by taking 5 µL of each metabolomics sample and thoroughly mixing them together. QC for untargeted lipidomics was also obtained by mixing 5 µL of each lipidomics sample. QC samples were measured at the beginning and every 10 injections within the randomized analytical batches.

To determine the total protein concentration of each pellet, BSA protein standard calibration curve with 9 BSA dilutions series (0 mg/mL to 2 mg/mL) was prepared. To do this, a mixed BCA solution was first prepared by mixing reagents A and B of the BCA kit in a ratio of 50:1. Then, 200 µL of the above solution was combined with 5 µL of each calibrator level in the 96 well plate. The protein amount was quantified by using Tecan Infinite 200 PRO (Männedorf, Switzerland) at wavelength of 562 nm and bandwidth 9 nm. Calibration curves samples were prepared freshly. For proteomics, the Thermo Scientific Pierce HeLa Protein Digest Standard, a highly validated mammalian protein digest, was used as a QC sample at the beginning and the end of the analytical batch to ensure equal LC-MS/MS performance.

Targeted metabolomics – UPLC settings

Amino acids and acylcarnitines

The quantitation method for amino acids and acylcarnitines is based on ref [5]. Briefly, chromatographic separation: Column and pre-column, Acquity® UPLC Premier BEH Amide (130 Å, 1.7 µm, 2.1 mm X 100 mm) connected to VanGuard UPLC BEH Amide pre-column (130 Å, 1.7 µm, 2.1 mm X 5 mm) (Waters Co., MA, USA); mobile phase composition, solvent A: 5 mM ammonium acetate (pH:3) and solvent B: ACN:H_2O (95:5, v/v) containing 5 mM ammonium acetate (pH:3); gradient program, 0 min, 100% B; 1.5 min, 100% B; 3.5 min, 92% B; 7 min, 90% B; 10 min, 78% B; 12 min, 65% B, 14 min, 2% of B; 15 min, 2% B; 16 min, 100% B; 22 min, 100% B; flow rate, 0.4 mL/min; injection volume, 1 µL; column oven temperature, 40 °C. Autosampler conditions: temperature, 10 °C; rinsing solvent, MeOH:H_2O (5:5, v/v) (channels R1, R2) and 10% IPA (channel R0); rinsing program, outer surface of the needle before and after aspiration with rinsing speed 35µL/s and rinsing volume 500µL. Electrospray ionization source parameters: ionization mode, positive; Gas 1 55 psi, Gas 2 65 psi, curtain gas 35 psi, temperature 400 °C, ions spray voltage 5500 V, entrance potential, 10 V. MRM conditions for measuring amino acids and acylcarnitines are given in **Supplementary Data, Table S3**.

Nucleotides and nucleosides

The method was established based on ref [6] with some extensions:

Chromatographic separation: column and pre-column, Acquity® UPLC Premier BEH Amide (130 Å, 1.7 µm, 2.1 mm X 100 mm) connected to VanGuard UPLC BEH Amide pre-column (130 Å, 1.7 µm, 2.1 mm X 5 mm) (Waters Co., MA, USA); mobile phase composition, solvent A: ACN:H_2O (50:50, v/v) containing 5 mM ammonium acetate (pH:9.5) and solvent B: ACN:H_2O (95:5, v/v) containing 5 mM ammonium acetate (pH:9.5); gradient program, 0 min, 97% B; 16 min, 50% B; 17:3 min, 50% B; 22 min, 0% B; 24 min, 97% B; 30 min, 97% B; flow rate, 0.4 mL/min; injection volume, 1 µL; column oven temperature, 40 °C. Autosampler conditions: temperature, 10 °C; rinsing solvent, MeOH:H_2O (5:5, v/v) (channels R1, R2) and 10% IPA (channel R0); rinsing program, outer surface of the needle before and after aspiration with rinsing speed 35µL/s and rinsing volume 500µL. Electrospray ionization source parameters: ionization mode, negative; Gas 1, 55 psi; Gas 2, 65 psi; Curtain gas, 35 psi; temperature, 450 °C; ions spray voltage, -4500 V; entrance potential, -10 V. MRM conditions for detection of nucleotides and nucleosides are given in **Supplementary Data, Table S4**.

Short chain fatty acids/organic acids-3-nitrophenylhydrazines (3-NPH) derivatives

The method was developed based on mixing the two developed methods by Han *et al.*[3, 4]:

Chromatographic separation: column and pre-column, Kinetex C18 (1.7 µm C18 100 X 2.1 mm) connected to VanGuard pre-column (Phenomenex, Aschaffenburg, Germany); mobile phase composition, solvent A: 0.1% formic acid and solvent B: ACN with 0.1% formic acid; gradient program, 0 min, 23% B; 3 min, 23% B; 4 min, 30% B; 6.5 min, 40% B; 8 min, 100% B; 9.5min, 100% B; 9.6 min, 23% B; 12min, 23% B; flow rate, 0.4 mL/min; injection volume, 1 µL; column oven temperature, 40 °C. Autosampler conditions: temperature, 10 °C; rinsing solvent, MeOH:H_2O (5:5, v/v) (channels R1, R2) and 10% IPA (channel R0); rinsing program, internal/external surface of the needle before and after aspiration with dip time 5 s, rinsing speed 35µL/s and rinsing volume 500µL. Electrospray ionization source parameters: ionization mode, negative; Gas 1, 55 psi; Gas 2, 65 psi; Curtain gas, 35 psi; temperature, 450 °C; ions spray voltage, -4500 V; entrance potential, -10 V. MRM conditions for detection of short chain fatty acids and organic acids are given in **Supplementary Data, Table S5**.

Free fatty acids

The method was developed according to ref [6, 7] with some modifications and extensions.

Chromatographic separation: Column and pre-column, Acquity® UPLC Premier BEH C18 (1.7 µm, 2.1 mm X 100 mm) connected to VanGuard UPLC BEH C18 pre-column (130 Å, 1.7 µm, 2.1 mm X 5 mm) (Waters Co., MA, USA); mobile phase composition, solvent A: ACN:H_2O (60:40, v/v) containing 5 mM ammonium acetate (pH:5) and solvent B: IPA:ACN:H_2O (45:55:5, v/v) containing 5 mM ammonium acetate (pH:5); gradient program, 0 min, 32% B; 1.5 min, 32% B; 3.8 min, 45% B; 5 min, 45 % B; 10 min, 65% B; 15 min, 97% B; 17.5 min, 97% B; 17.8 min, 32%B;

22 min, 32%B; flow rate, 0.3 mL/ min; injection volume, 2 µL; column oven temperature, 40 °C; 40 °C. Autosampler conditions: temperature, 10 °C; rinsing solvent, MeOH:H_2O (5:5, v/v) (channels R1, R2) and 10% IPA (channel R0); rinsing program, outer surface of the needle before and after aspiration with rinsing speed 35µL/s and rinsing volume 500µL. Electrospray ionization source parameters: ionization mode, negative; Gas 1, 55 psi; Gas 2, 65 psi; Curtain gas, 35 psi; temperature, 450 °C; ions spray voltage, -4500 V; entrance potential, -10 V. MRM conditions for detection of free fatty acids are given in **Supplementary Data, Table S6**.

Mouse SPLASH® LIPIDOMIX® Mass Spec Standard

The method was fixed based on ref [8] [9] with some modification.

Chromatographic separation: Column and pre-column, Acquity® UPLC Premier BEH Amide (1.7 µm, 2.1 mm X 100 mm) connected to VanGuard UPLC BEH Amide pre-column (130 Å, 1.7 µm, 2.1 mm X 5 mm) (Waters Co., MA, USA); mobile phase composition, solvent A: H_2O:ACN (50:50, v/v) containing 5 mM ammonium acetate (pH:8) and solvent B: ACN:H_2O (95:5, v/v) containing 5 mM ammonium acetate (pH:8); gradient program, 0 min, 99.9% B; 5 min, 99.9% B; 15 min, 95% B; 17 min, 5% B; 21 min, 5% B; 21.5min, 99.9% B; 26 min, 99.9% B; flow rate, 0.3 mL min^{-1}; injection volume, 5 µL; column and pre-column temperature, 40 °C. Autosampler conditions: temperature, 10 °C; rinsing solvent, MeOH:H_2O (5:5, v/v) (channels R1, R2) and 10% IPA (channel R0); rinsing program, outer surface of the needle before and after aspiration with rinsing speed 35µL/s and rinsing volume 500 µL. Electrospray ionization source parameters: ionization, positive (negative) mode; Gas 1, 50 psi; Gas 2, 60 psi; Curtain gas, 40 psi; temperature, 500 °C; ions spray voltage, 5500 (-4500 V); entrance potential, 10 (-10 V). MRM conditions for detection of Mouse SPLASH® LIPIDOMIX® Mass Spec Standard are given in **Supplementary Data, Table S7**.

Supplemental Figures

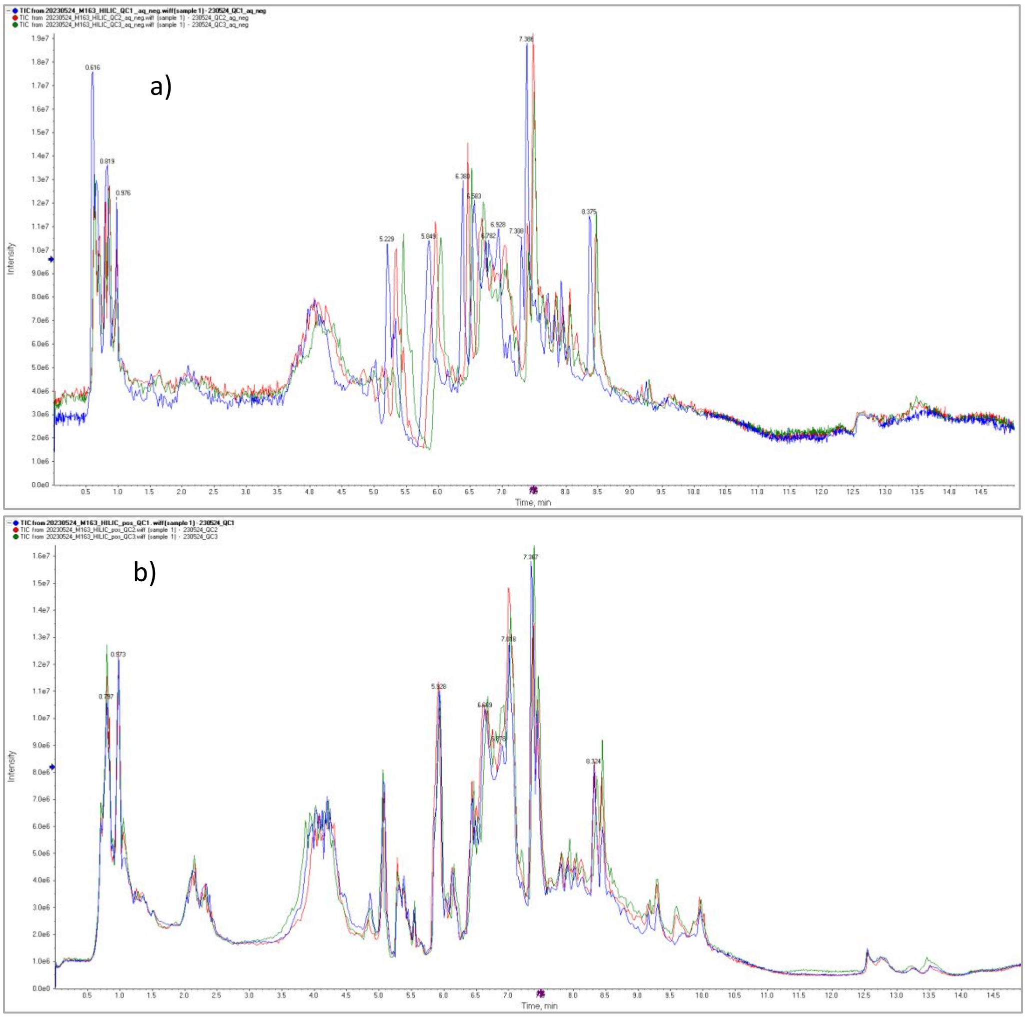

Figure S1 (a and b). QC chromatograms obtained by HILIC based untargeted metabolomics method in negative and positive modes, respectively.

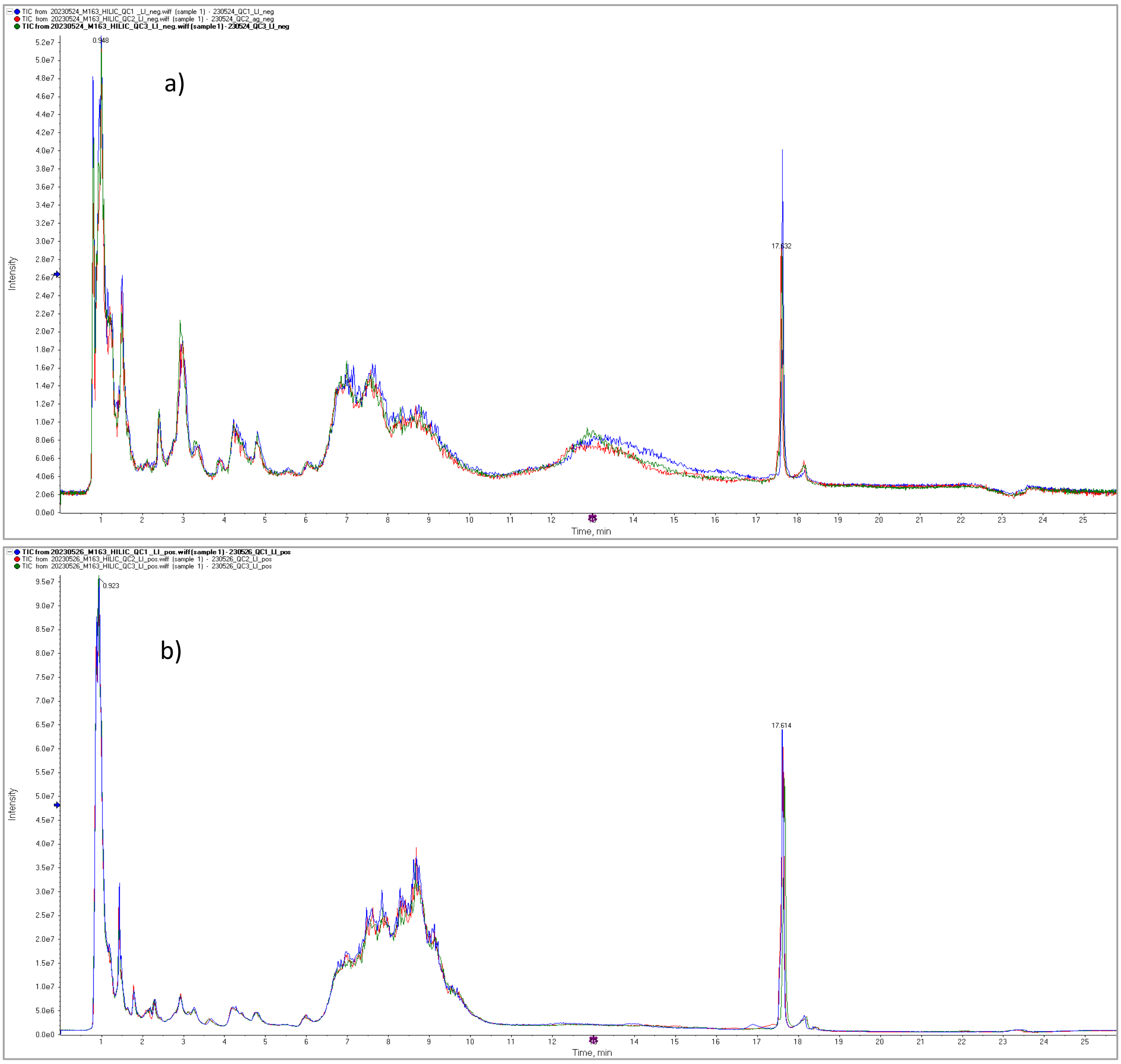

Figure S2 (a and b). QC chromatograms obtained by HILIC based untargeted lipidomics method in negative and positive modes, respectively.

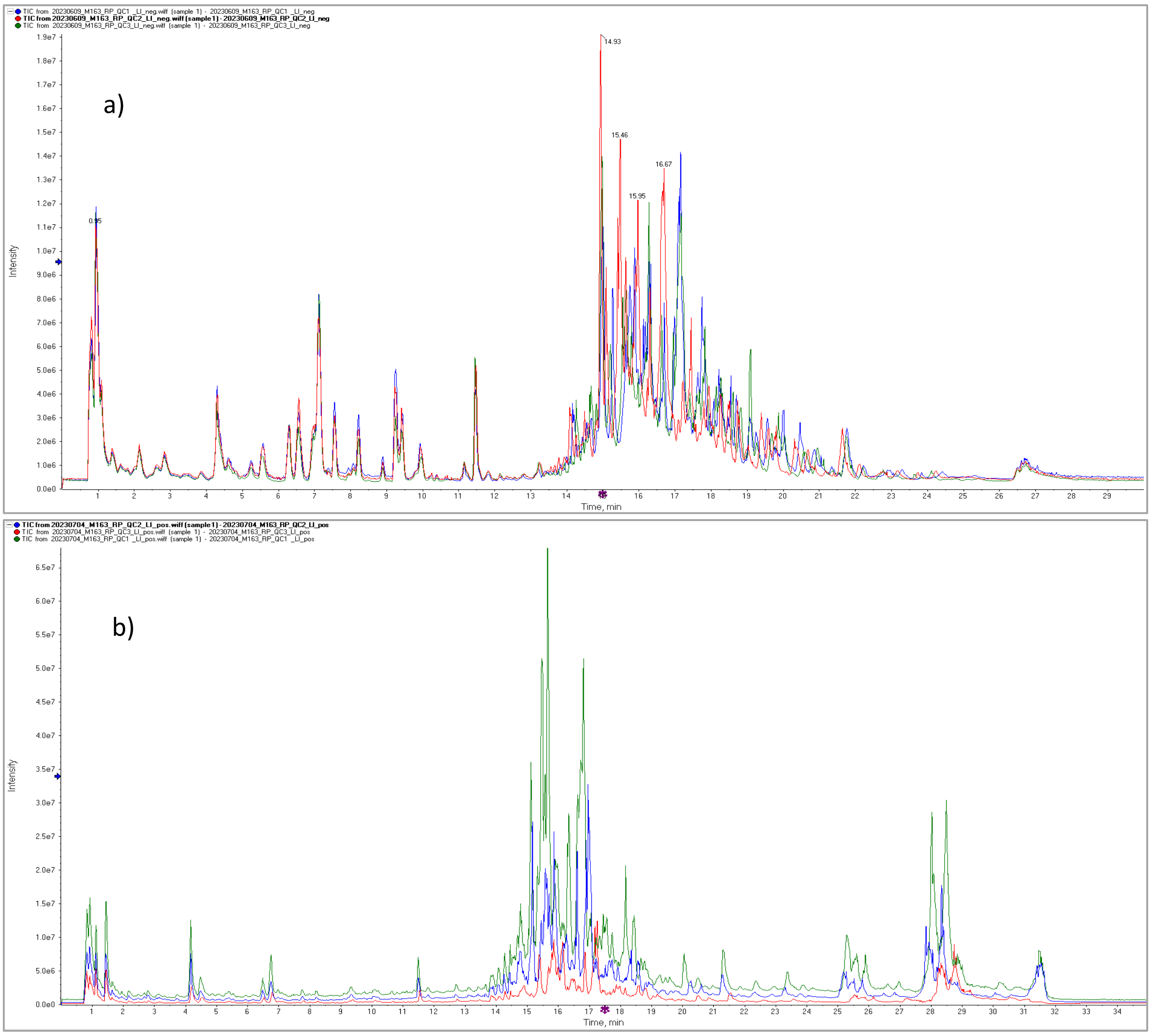

Figure S3 (a and b). QC chromatograms obtained by RP based untargeted lipidomics method in negative and positive modes, respectively.

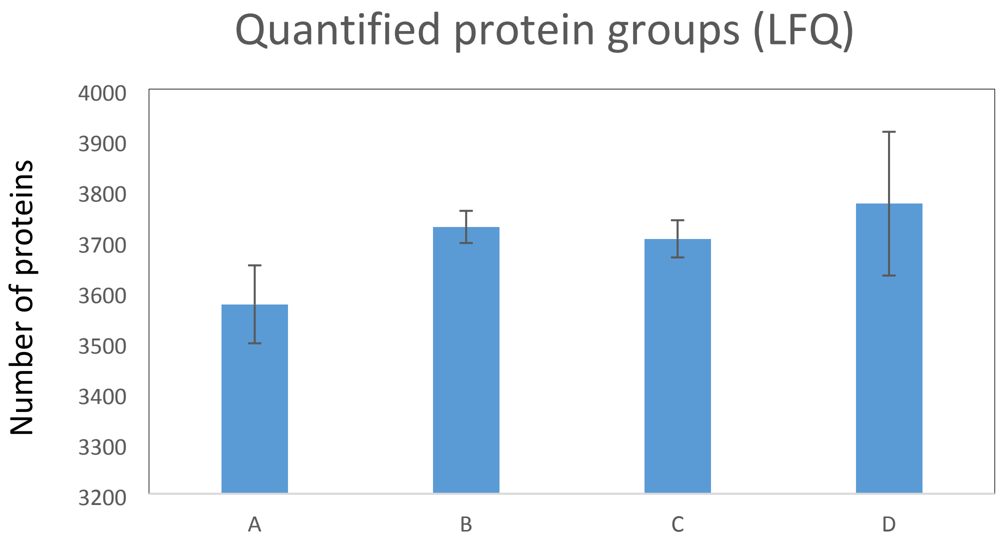

Figure S4 Identified/quantified protein groups for the extraction conditions with or without ammonium salt (A: MTBE/MeOH/H_2O (10:3:2.5, v/v/v), B:MTBE/MeOH/Ammonium salt solution (0.3M) (10:3:2.5, v/v/v), C: MTBE/MeOH/H_2O (7:3:2.5, v/v/v), D: MTBE/MeOH/ Ammonium salt solution (0.3M) (7:3:2.5, v/v/v)).

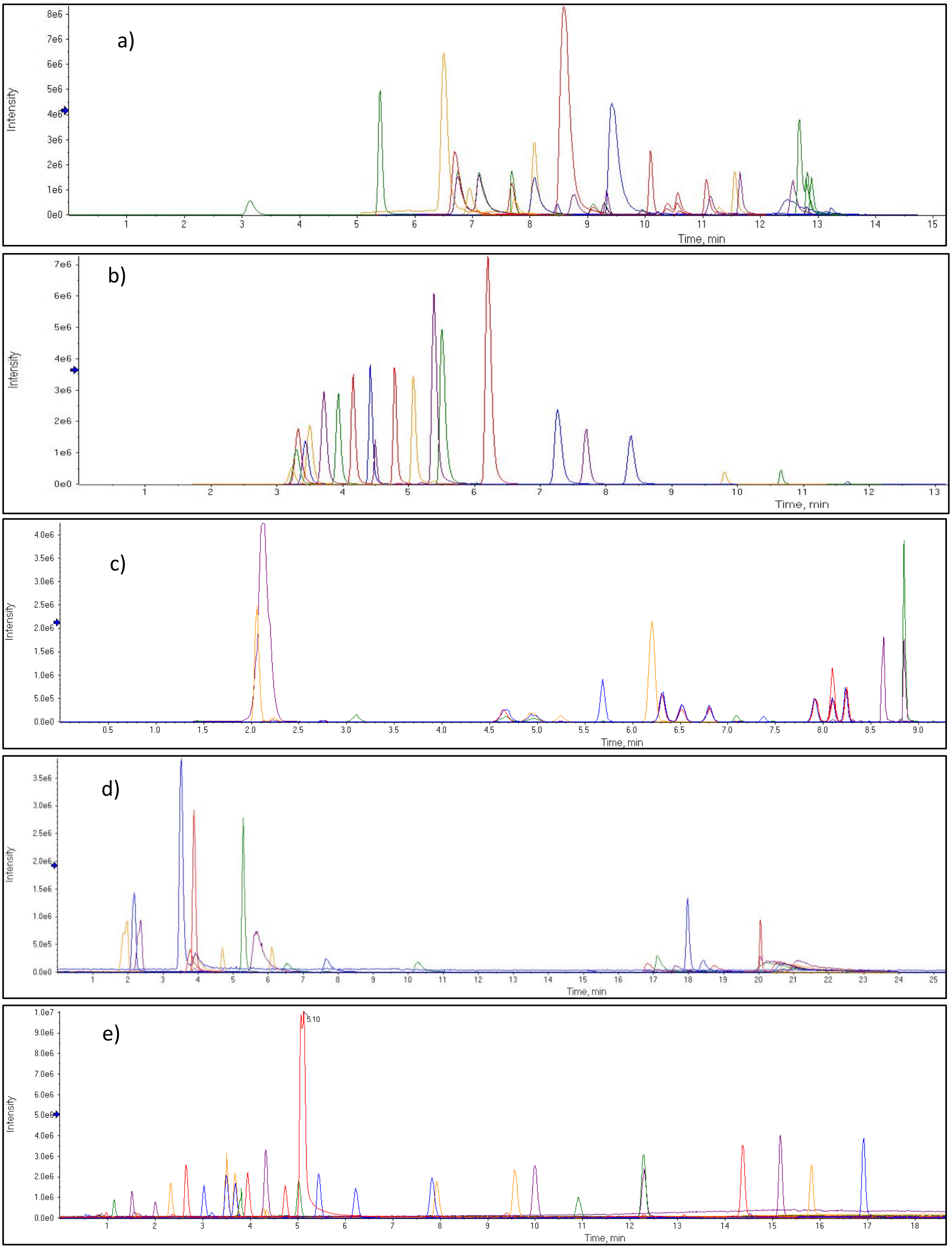

Figure S5: (a, b, c, d, and e). QC Chromatograms obtained by UHPLC-MS/MS for amino acids, acyl carnitines, short chain fatty acids and organic acids, nucleotides, and free fatty acids, respectively.

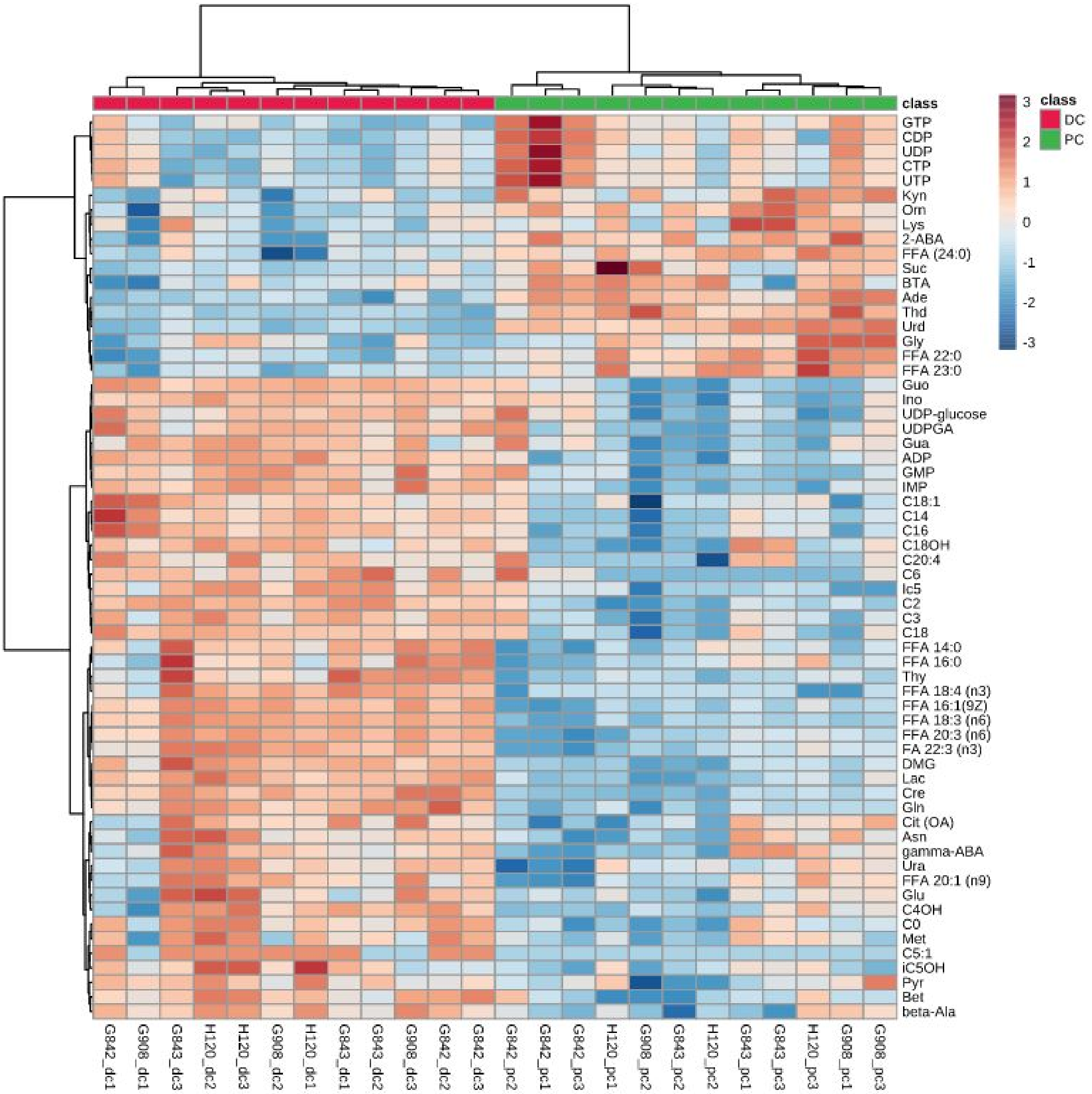

Figure S6: Heatmap (Distance measure: Euclidean, Cluster algorithm: Ward) for targeted metabolomics of the colon tissue (DC: distal colon, PC: proximal colon).

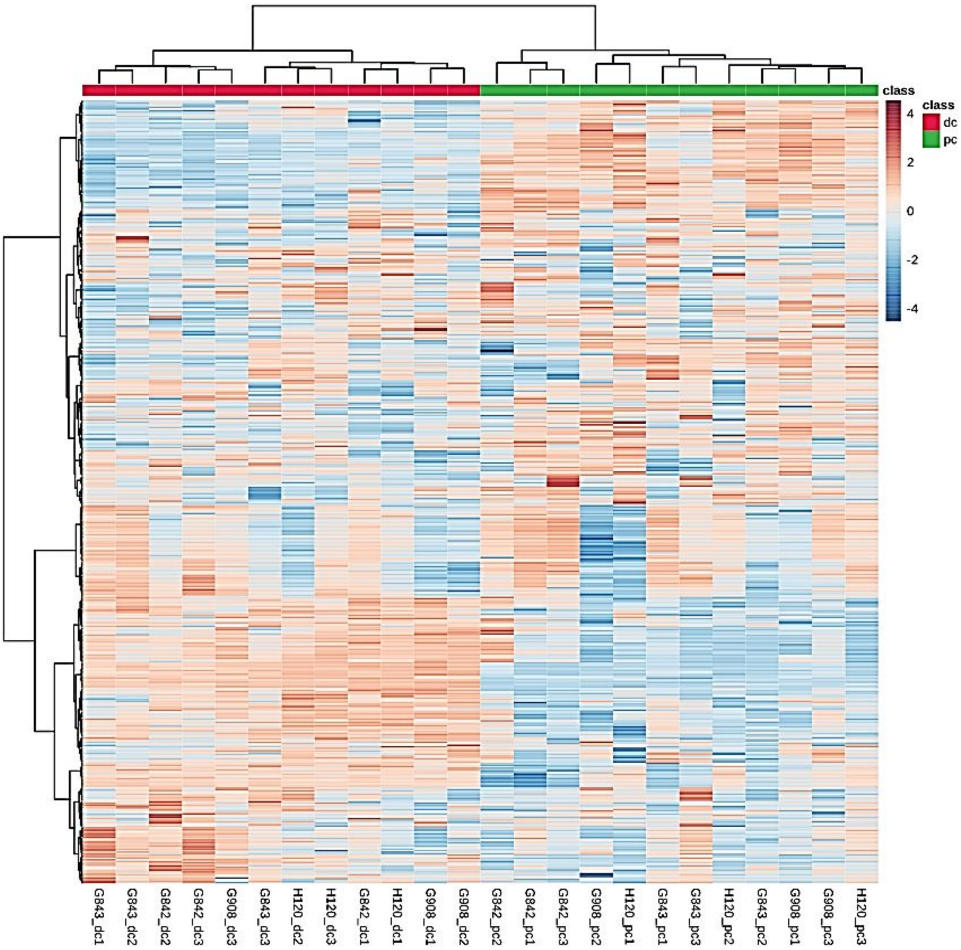

Figure S7 Heatmap (Distance measure: Euclidean, Cluster algorithm: Ward) for HILIC based colon tissue untargeted metabolomics (DC: distal colon, PC: proximal colon).

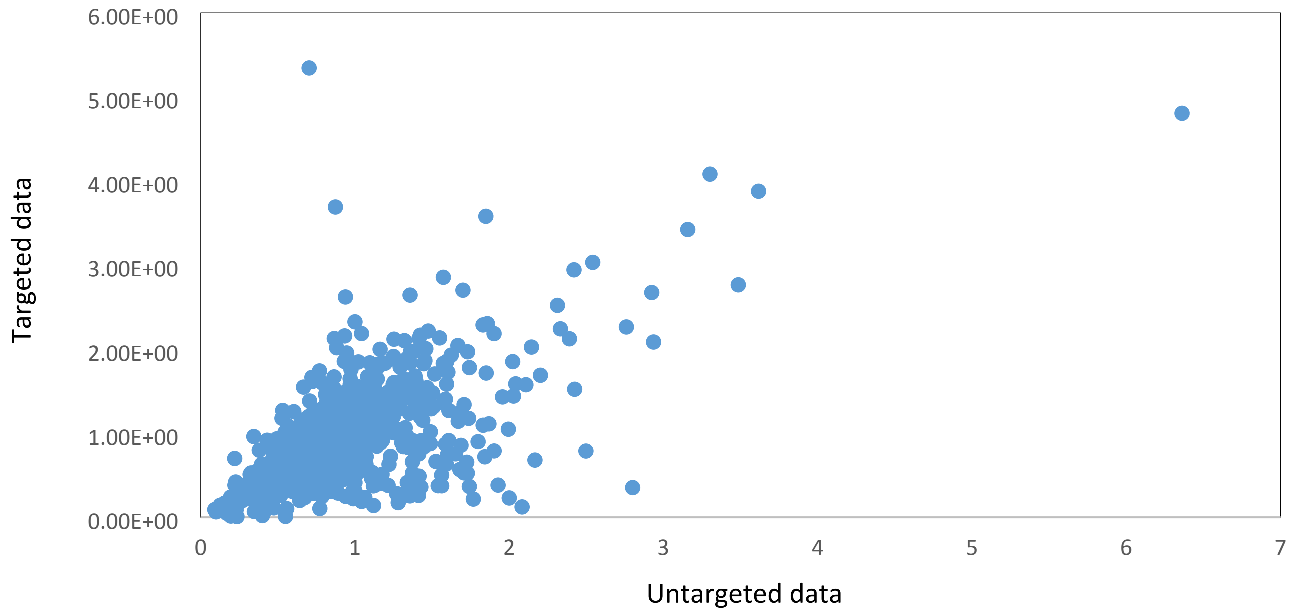

Figure S8 Scatter plot of the ratios PC and DC of the targeted and untargeted metabolomics data of each single biopsy sample of each respective metabolite. Only metabolites, which were present and correctly annotated in the targeted and untargeted datasets, were used. The correlation coefficient is 0.63.

(a)

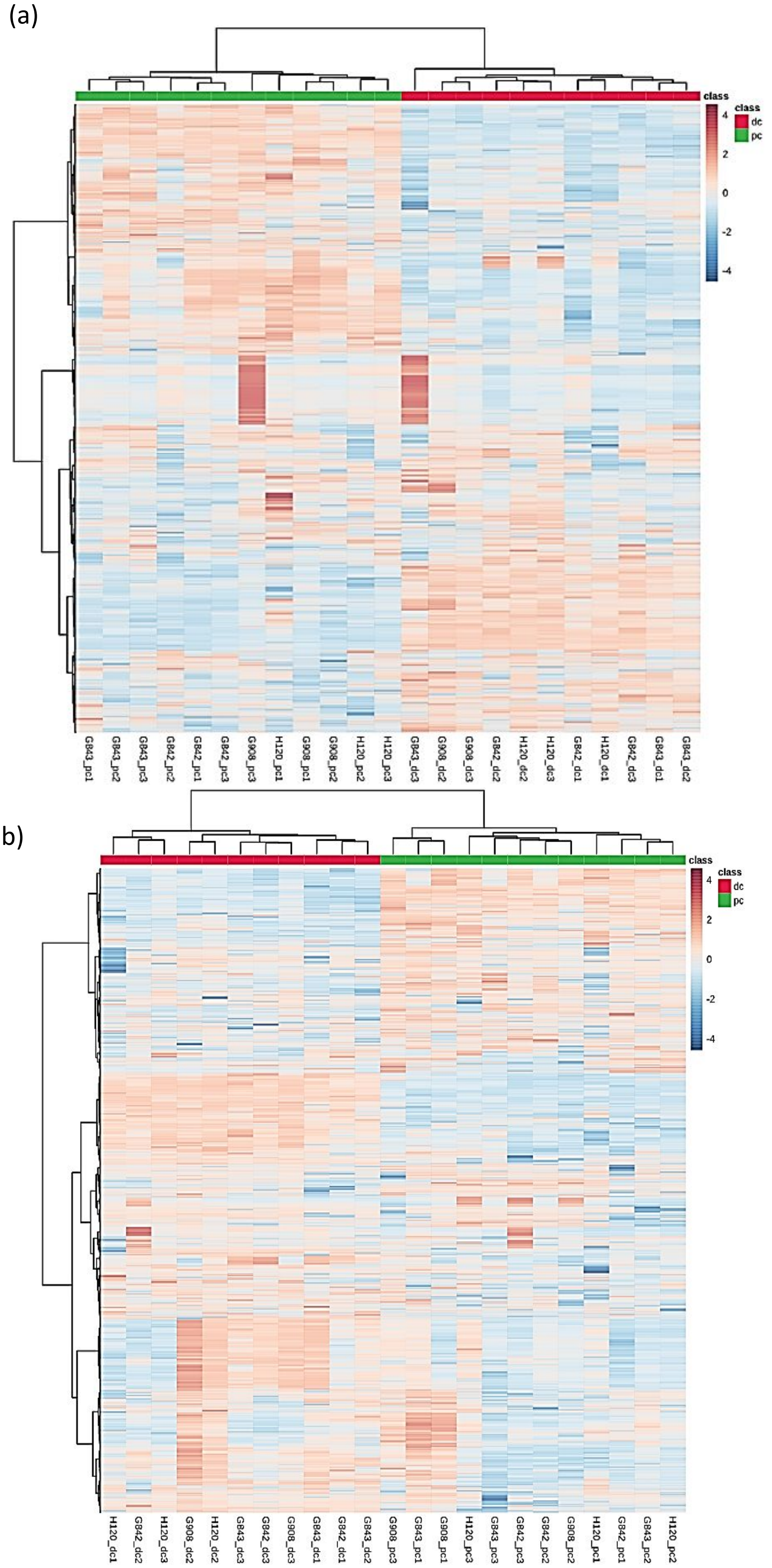

Figure S9 Heatmaps (Distance measure: Euclidean, Cluster algorithm: Ward) for a) HILIC and b) RP-LC based colon tissue lipidomics, respectively (DC: distal colon, PC: proximal colon).

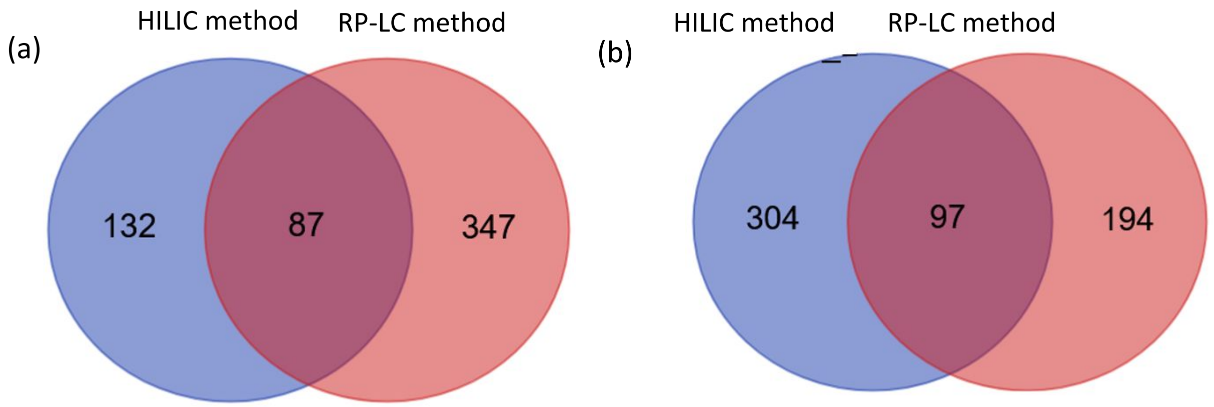

Figure S10 (a and b) Venn diagrams for significantly up/down-regulated lipids in DC tissue based on both HILIC and RP-LC based colon tissue lipidomics (DC: distal colon, PC: proximal colon).

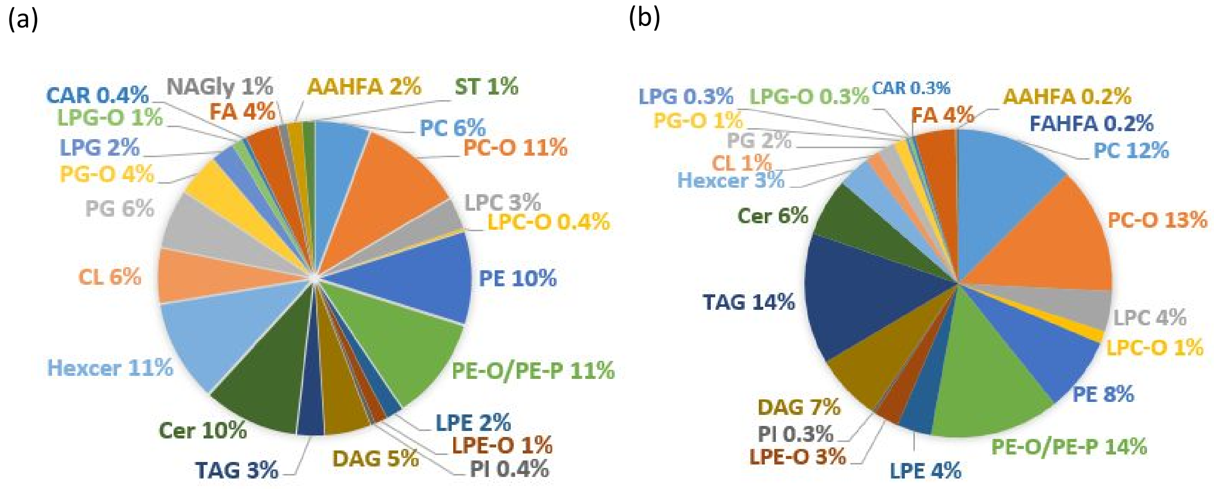

Figure S11 Pie charts showing percentages of significantly changed lipid classes and subclasses detected by HILIC and RP-LC methods, respectively.

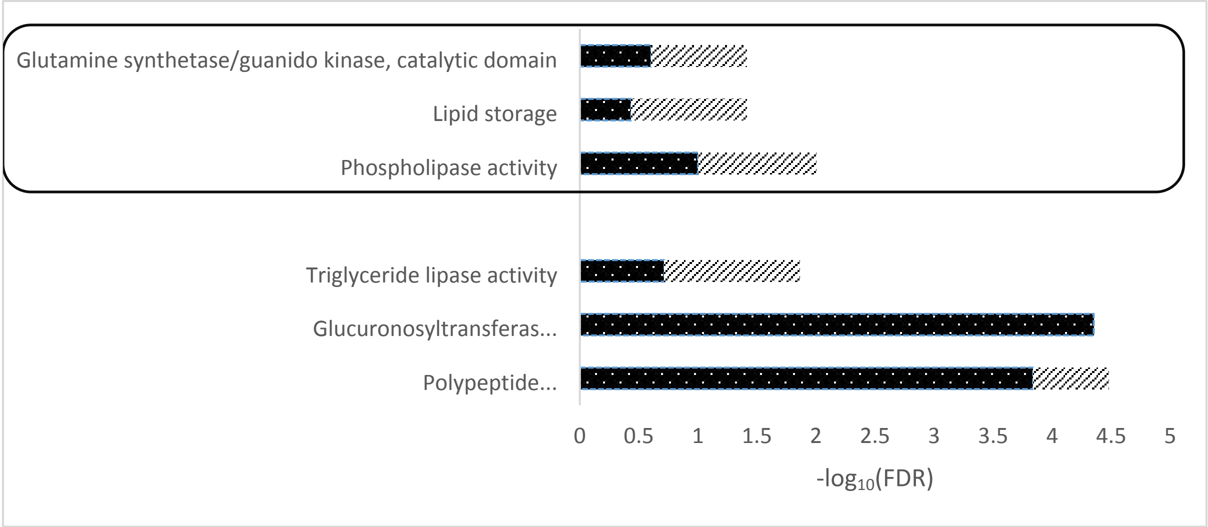

Figure S12 Significant gene ontology terms specific for the PC (bottom) and DC (top) tissue (-log_{10}(FDR)). The black shaded area present the percentage of the size overlap and the gene set.

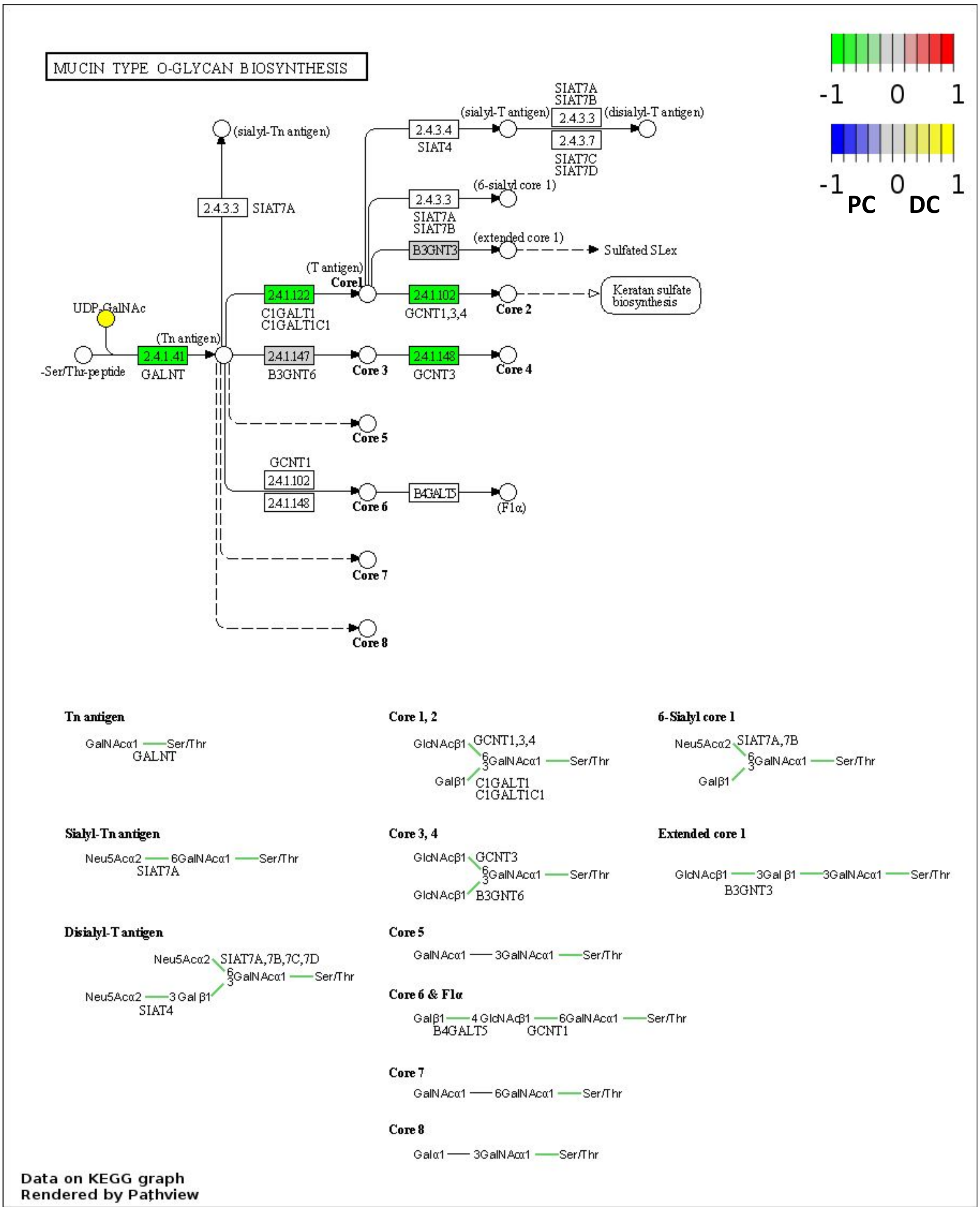

Figure S13 Mucin type O-glycan biosynthesis based on KEGG. Increased metabolite and protein concentrations in the PC tissue are colored in blue and green and increased metabolite and protein concentrations in DC tissue in yellow and red. The figure was created with Pathview.[10]

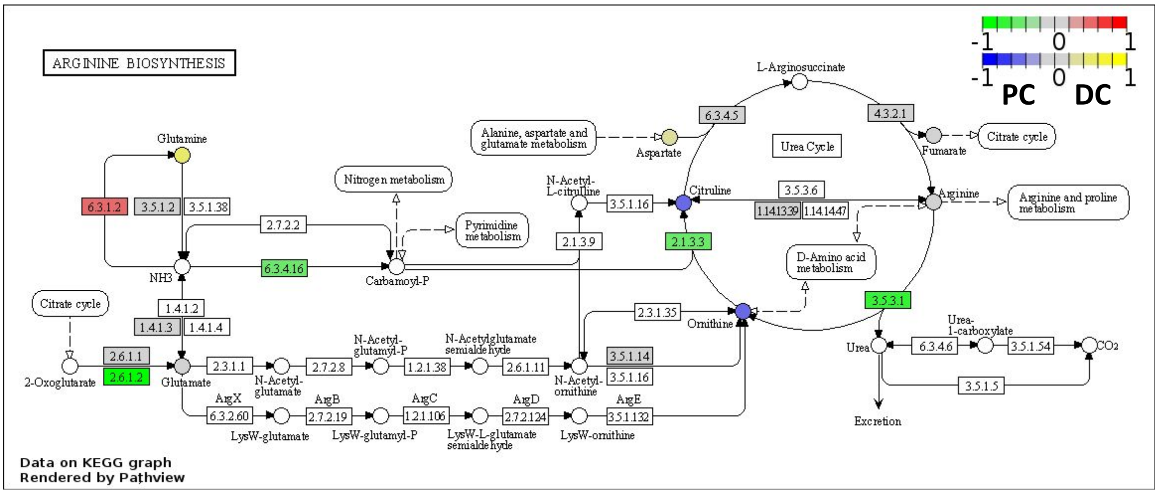

Figure S14 Arginine Biosynthesis based on KEGG. Increased metabolite and protein concentrations in the PC tissue are colored in blue and green and increased metabolite and protein concentrations in DC tissue in yellow and red. The figure was created with Pathview.[10]

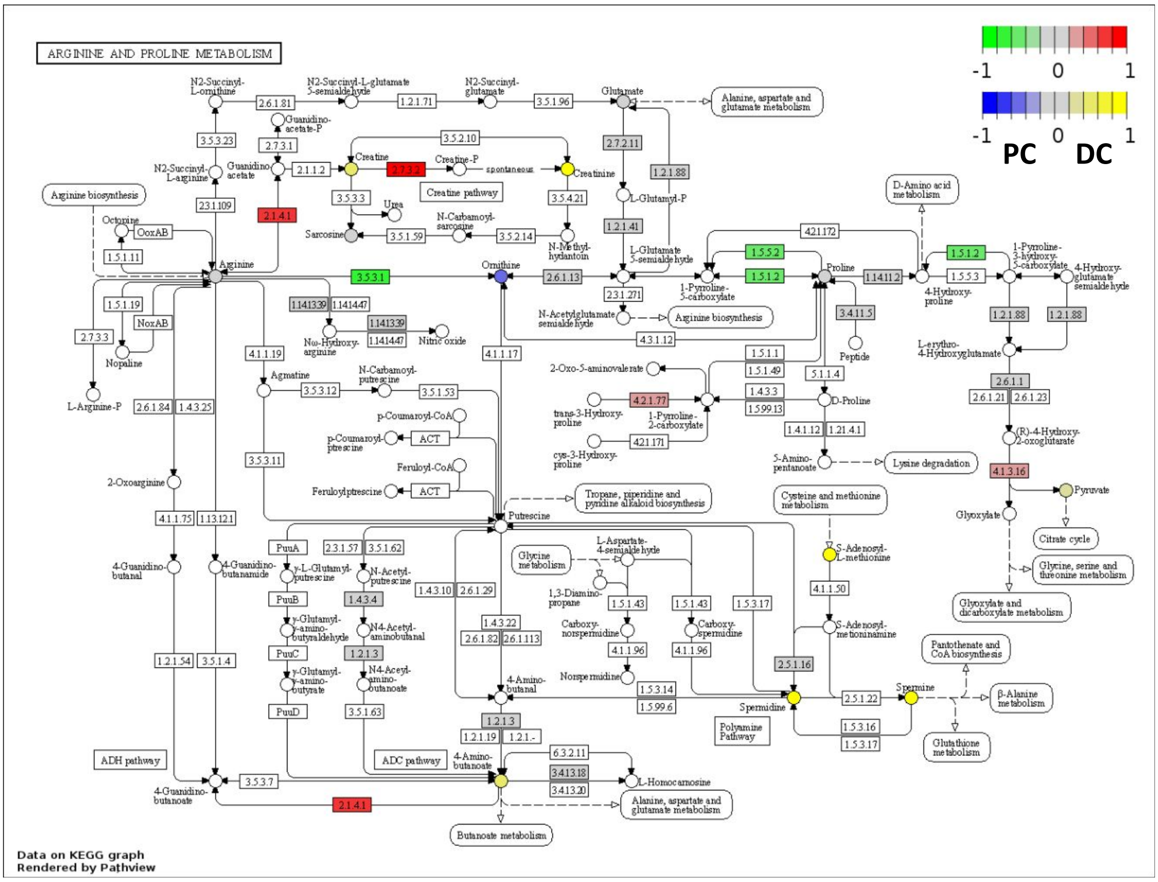

Figure S15 Arginine and proline metabolism based on KEGG. Increased metabolite and protein concentrations in the PC tissue are colored in blue and green and increased metabolite and protein concentrations in DC tissue in yellow and red. The figure was created with Pathview.[10]

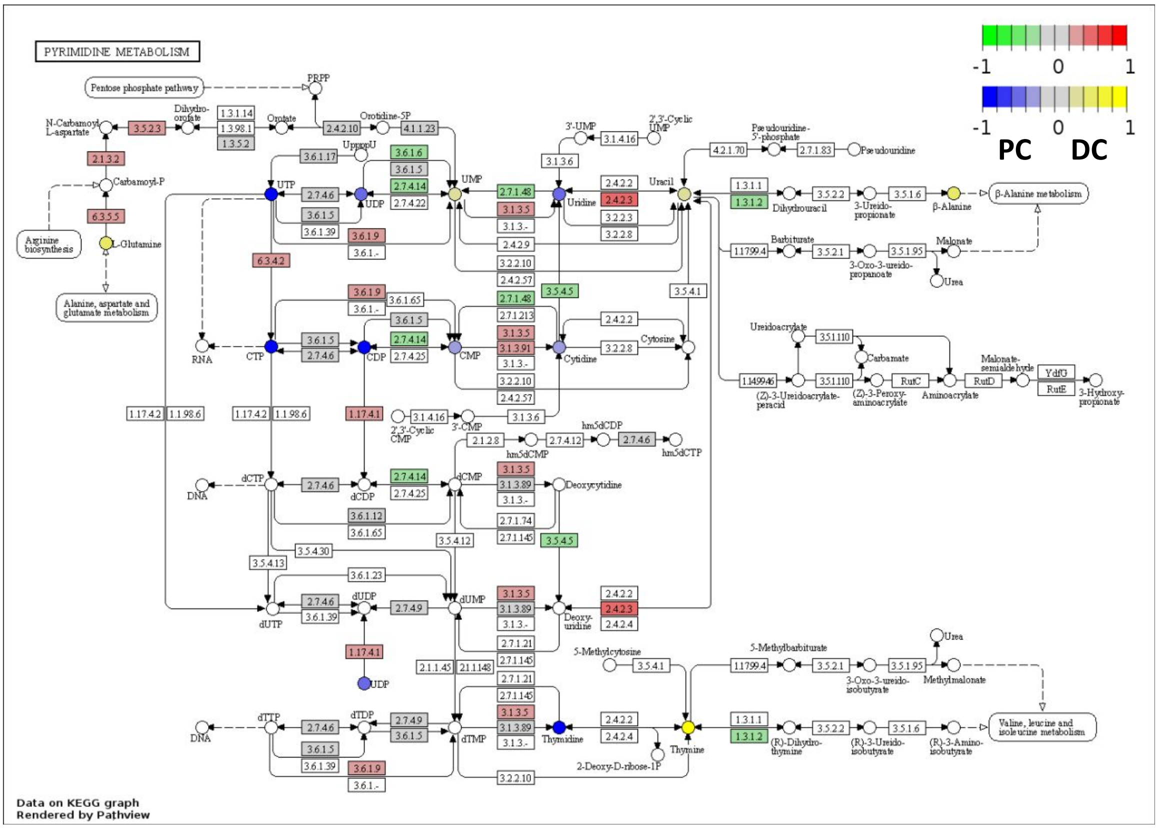

Figure S16 Pyrimidine metabolism based on KEGG. Increased metabolite and protein concentrations in the PC tissue are colored in blue and green and increased metabolite and protein concentrations in DC tissue in yellow and red. The figure was created with Pathview.[10]

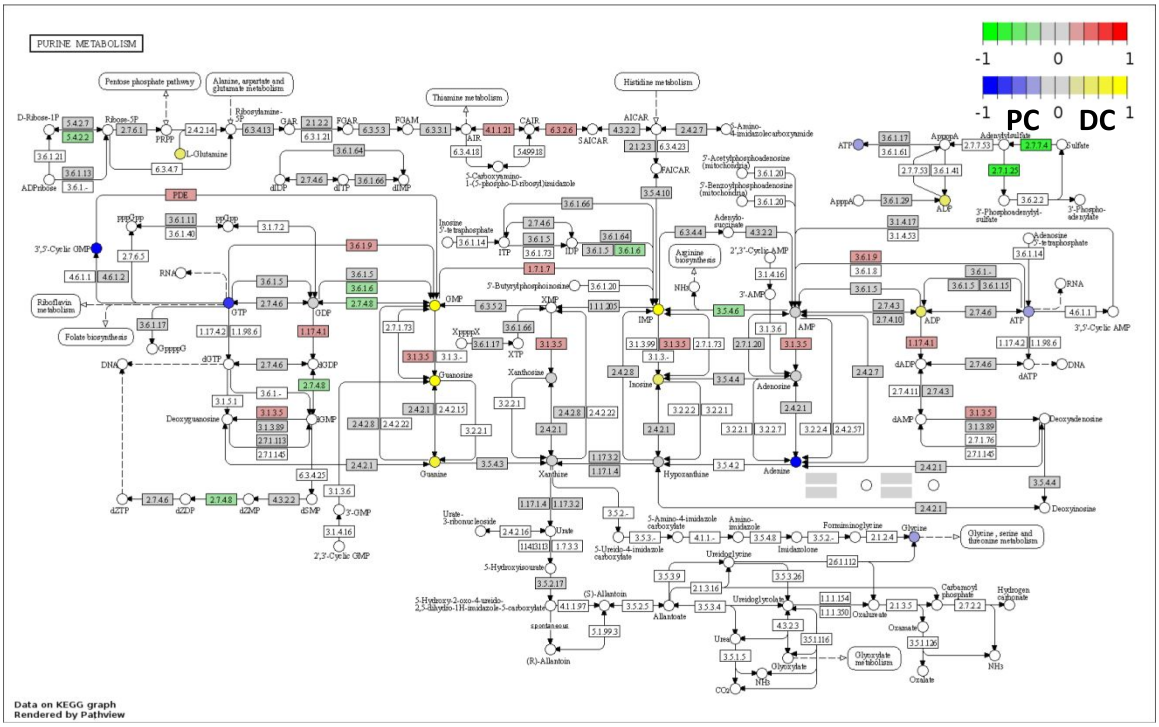

Figure S17 Purine metabolism based on KEGG. Increased metabolite and protein concentrations in the PC tissue are colored in blue and green and increased metabolite and protein concentrations in DC tissue in yellow and red. The figure was created with Pathview.[10]

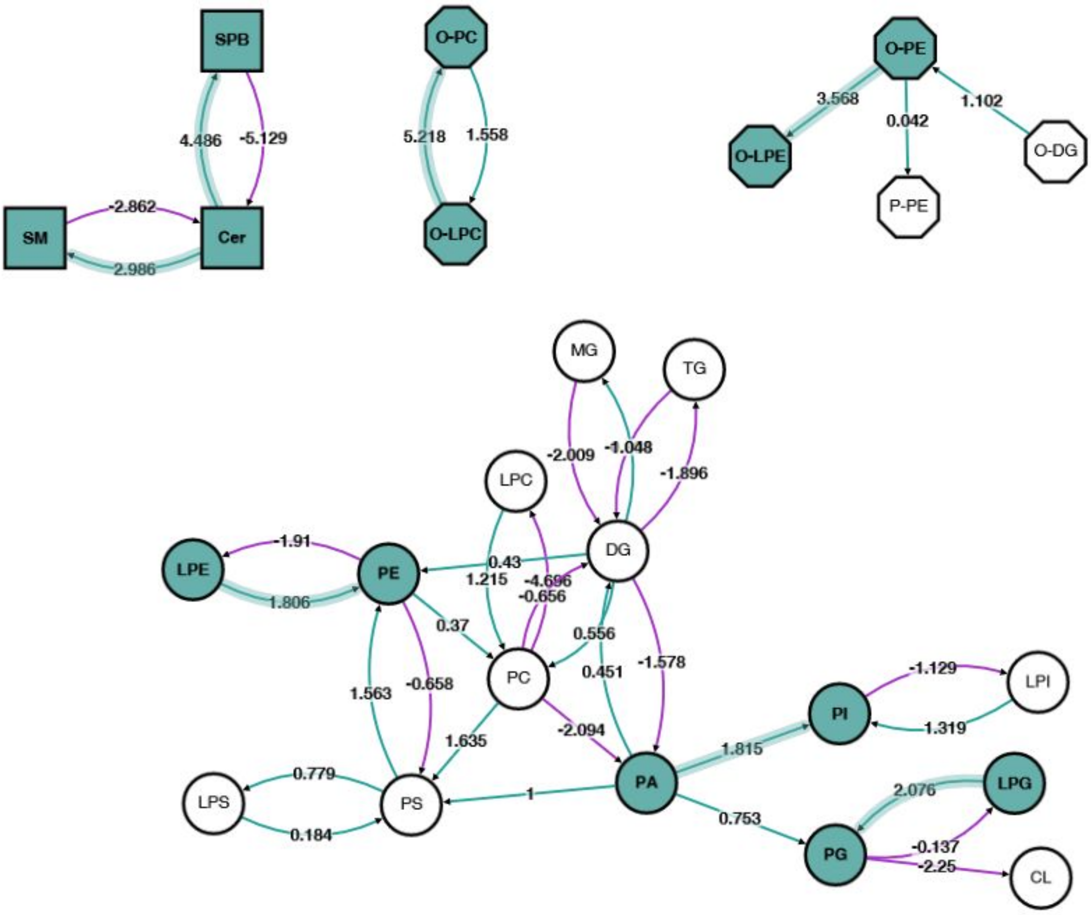

Figure S18 BioPAN lipid sub-classes networks and reactions for PC tissue versus DC tissue based on an active status, (obtained from the merged data set of HILIC and RP-based untargeted lipidomics data) (White nodes: no status, Green nodes: active status, Green arrows: reactions with a positive Z score, Purple arrows: reactions with a negative Z score, Green shaded arrows (for both green and purple arrows): Significant reactions based on p value < 0.05 (Z-score >1.645), no paired data).

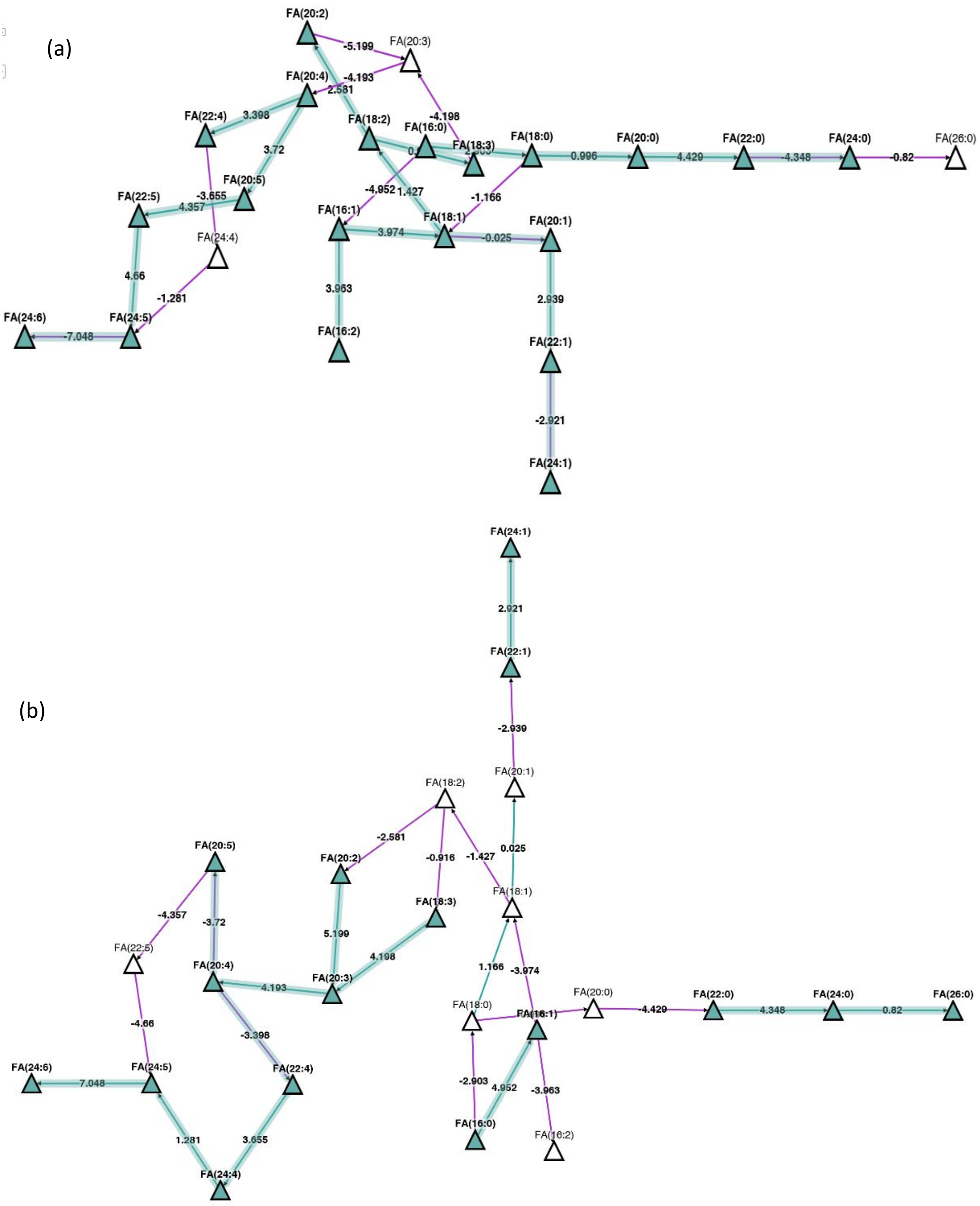

Figure S19 (a and b) BioPAN networks based on fatty acid for PC tissue versus DC tissue based on active and suppressed pathways, respectively (both HILIC and RP-based untargeted lipidomics data), (White nodes: no status, Green nodes: active/suppressed status, Green arrows: reactions with a positive Z score, Purple arrows: reactions with a negative Z score, Green shaded arrows (for both green and purple arrows): Z-score (positive or negative) implied in an active/suppressed status, p value < 0.05, and no paired data).

References

(1) Patterson, R. E.; Ducrocq, A. J.; McDougall, D. J.; Garrett, T. J.; Yost, R. A. Comparison of blood plasma sample preparation methods for combined LC-MS lipidomics and metabolomics. *Journal of chromatography. B, Analytical technologies in the biomedical and life sciences* **2015**, *1002*, 260-266. DOI: 10.1016/j.jchromb.2015.08.018 From NLM Medline.

(2) Sostare, J.; Di Guida, R.; Kirwan, J.; Chalal, K.; Palmer, E.; Dunn, W. B.; Viant, M. R. Comparison of modified Matyash method to conventional solvent systems for polar metabolite and lipid extractions. *Anal Chim Acta* **2018**, *1037*, 301-315. DOI: 10.1016/j.aca.2018.03.019 From NLM Medline.

(3) Han, J.; Gagnon, S.; Eckle, T.; Borchers, C. H. Metabolomic analysis of key central carbon metabolism carboxylic acids as their 3-nitrophenylhydrazones by UPLC/ESI-MS. *Electrophoresis* **2013**, *34* (19), 2891-2900. DOI: 10.1002/elps.201200601 From NLM.

(4) Han, J.; Lin, K.; Sequeira, C.; Borchers, C. H. An isotope-labeled chemical derivatization method for the quantitation of short-chain fatty acids in human feces by liquid chromatography–tandem mass spectrometry. *Analytica Chimica Acta* **2015**, *854*, 86-94. DOI: http://dx.doi.org/10.1016/j.aca.2014.11.015.

(5) Wudy, S. I.; Mittermeier-Klessinger, V. K.; Dunkel, A.; Kleigrewe, K.; Ensenauer, R.; Dawid, C.; Hofmann, T. F. High-Throughput Analysis of Underivatized Amino Acids and Acylcarnitines in Infant Serum: A Micromethod Based on Stable Isotope Dilution Targeted HILIC-ESI-MS/MS. *J Agric Food Chem* **2023**, *71* (22), 8633-8647. DOI: 10.1021/acs.jafc.3c00962 From NLM Medline.

(6) Fromme, T.; Kleigrewe, K.; Dunkel, A.; Retzler, A.; Li, Y.; Maurer, S.; Fischer, N.; Diezko, R.; Kanzleiter, T.; Hirschberg, V.; et al. Degradation of brown adipocyte purine nucleotides regulates uncoupling protein 1 activity. *Molecular metabolism* **2017**, *8*, 77-85. DOI: https://doi.org/10.1016/j.molmet.2017.12.010.

(7) Hellmuth, C.; Weber, M.; Koletzko, B.; Peissner, W. Nonesterified fatty acid determination for functional lipidomics: comprehensive ultrahigh performance liquid chromatography-tandem mass spectrometry quantitation, qualification, and parameter prediction. *Anal Chem* **2012**, *84* (3), 1483-1490. DOI: 10.1021/ac202602u From NLM.

(8) Mackenzie Pearson, S. K. K.; Norris, P.; Hunter, C. Achieve broad lipid quantitation using a high-throughput targeted lipidomics method. *SCIEX* **2018**.

(9) Bhaskar, A. K.; Naushin, S.; Ray, A.; Singh, P.; Raj, A.; Pradhan, S.; Adlakha, K.; Siddiqua, T. J.; Malakar, D.; Dash, D. A high throughput Lipidomics method using scheduled multiple reaction monitoring. *Biomolecules* **2022**, *12* (5), 709.

(10) Luo, W.; Pant, G.; Bhavnasi, Y. K.; Blanchard, S. G., Jr.; Brouwer, C. Pathview Web: user friendly pathway visualization and data integration. *Nucleic acids research* **2017**, *45* (W1), W501-w508. DOI: 10.1093/nar/gkx372 From NLM.

APPENDIX

Appendix 1 MS/MS annotation for metabolites by using MS DIAL.

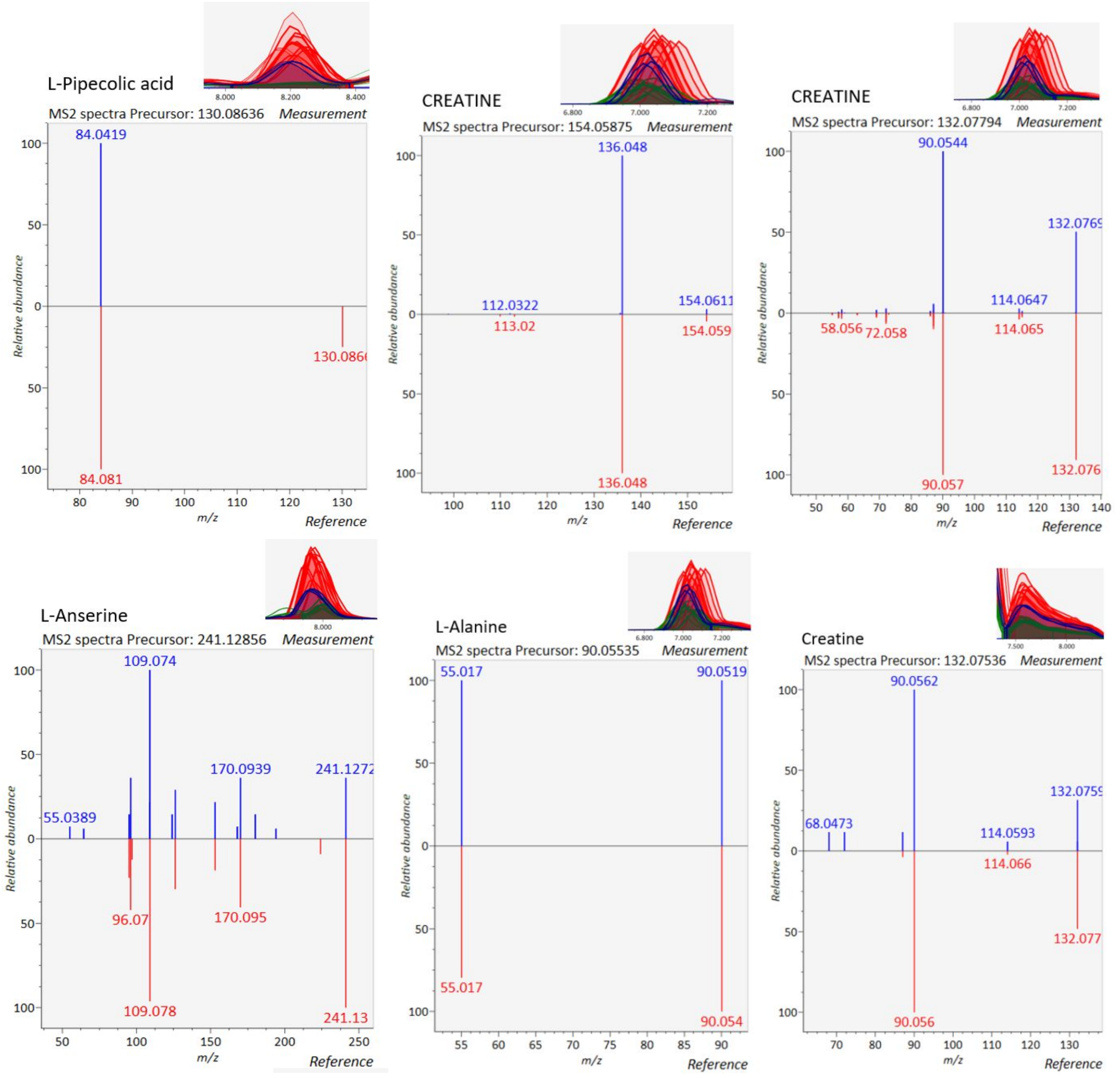

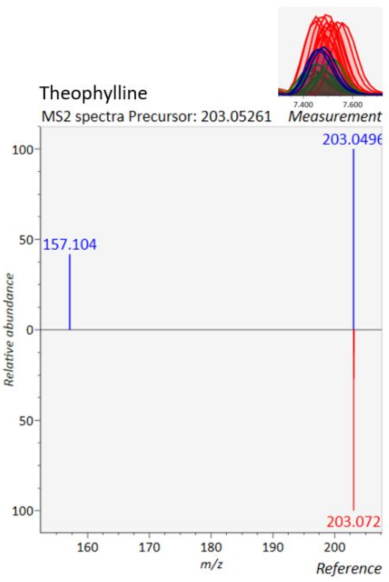
Theophylline
MS2 spectra Precursor: 203.05261
Measurement
157.104
203.0496
203.072
Relative abundance
m/z
Reference

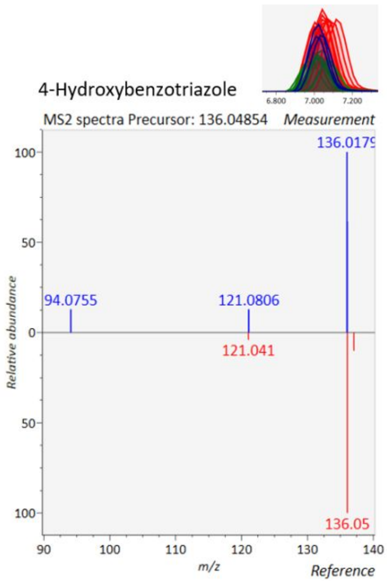
4-Hydroxybenzotriazole
MS2 spectra Precursor: 136.04854
Measurement
94.0755
121.0806
136.0179
121.041
136.05
Relative abundance
m/z
Reference

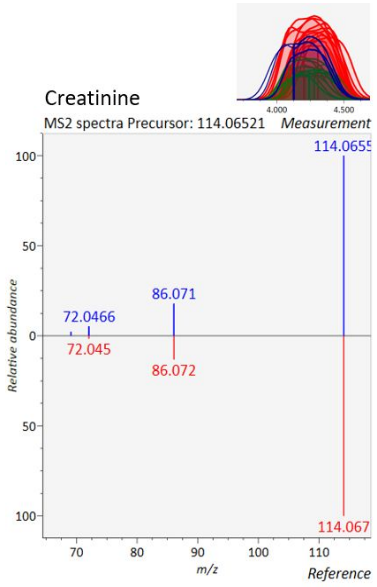
Creatinine
MS2 spectra Precursor: 114.06521
Measurement
72.0466
86.071
114.0655
72.045
86.072
114.067
Relative abundance
m/z
Reference

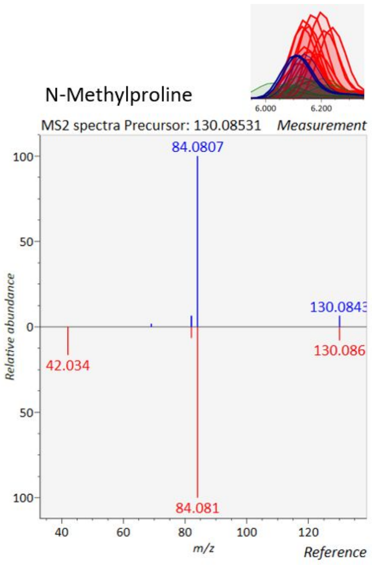
N-Methylproline
MS2 spectra Precursor: 130.08531
Measurement
84.0807
130.0843
42.034
130.086
84.081
Relative abundance
m/z
Reference

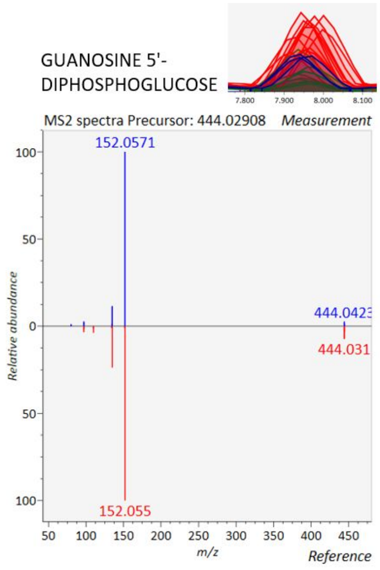
GUANOSINE 5'-
DIPHOSPHOGLUCOSE
MS2 spectra Precursor: 444.02908
Measurement
152.0571
444.0423
444.031
152.055
Relative abundance
m/z
Reference

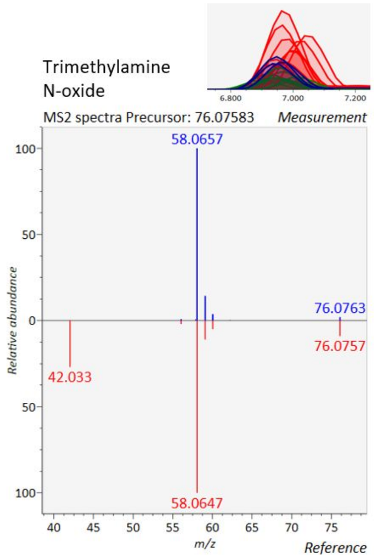
Trimethylamine
N-oxide
MS2 spectra Precursor: 76.07583
Measurement
58.0657
76.0763
76.0757
42.033
58.0647
Relative abundance
m/z
Reference

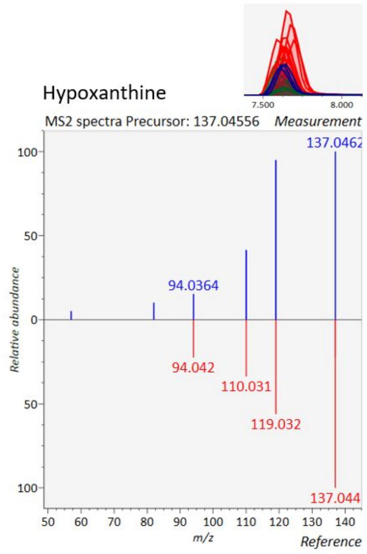
Hypoxanthine
MS2 spectra Precursor: 137.04556
Measurement
137.0462
94.0364
94.042
110.031
119.032
137.044
Relative abundance
m/z
Reference

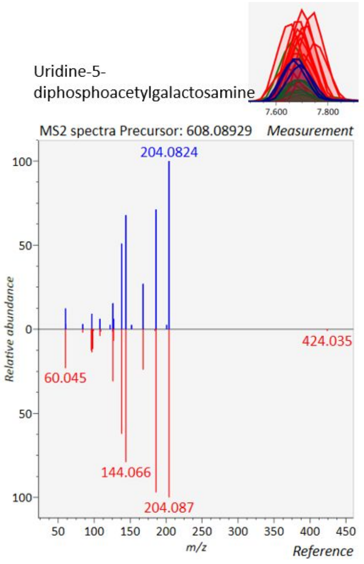
Uridine-5-
diphosphoacetylgalactosamine
MS2 spectra Precursor: 608.08929
Measurement
204.0824
60.045
424.035
144.066
204.087
Relative abundance
m/z
Reference

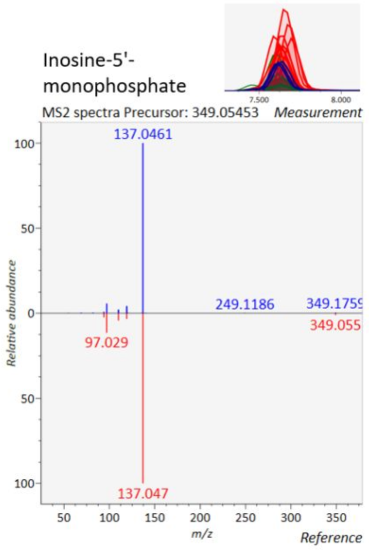
Inosine-5'-
monophosphate
MS2 spectra Precursor: 349.05453
Measurement
137.0461
249.1186
349.1759
97.029
349.055
137.047
Relative abundance
m/z
Reference

Adenosine_Diphosphate

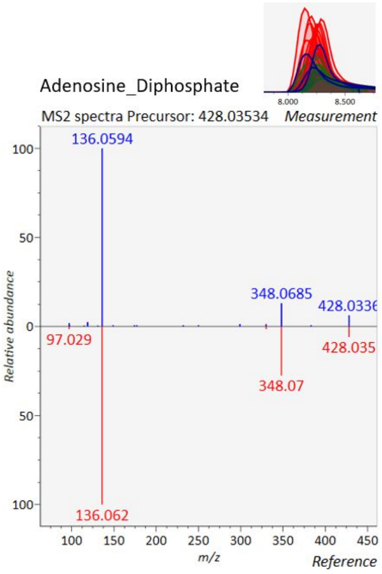

Pyrrolidine

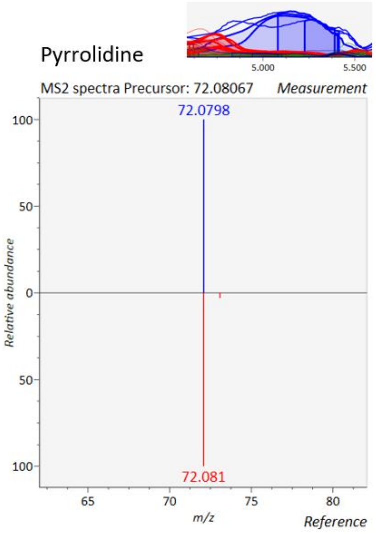

Spermidine

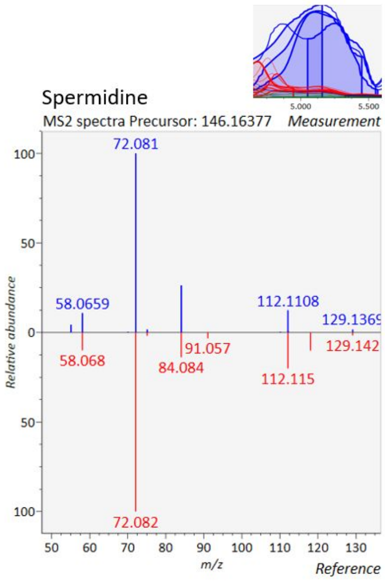

GLYCEROL 2-PHOSPHATE

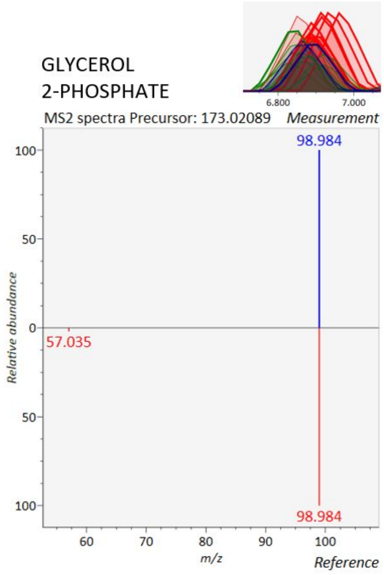

CARNITINE

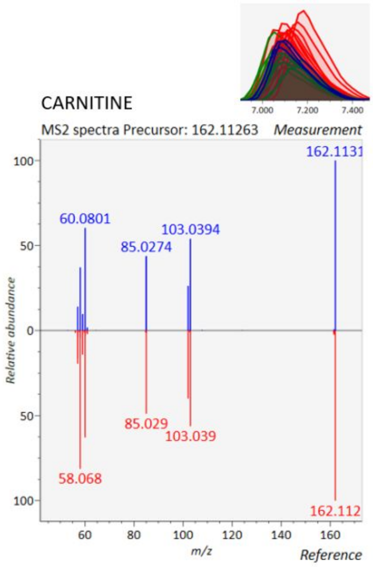

S-Adenosyl-L-homocysteine

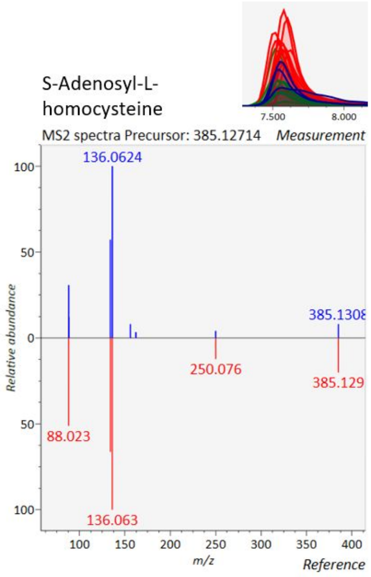

Pyrrolidine

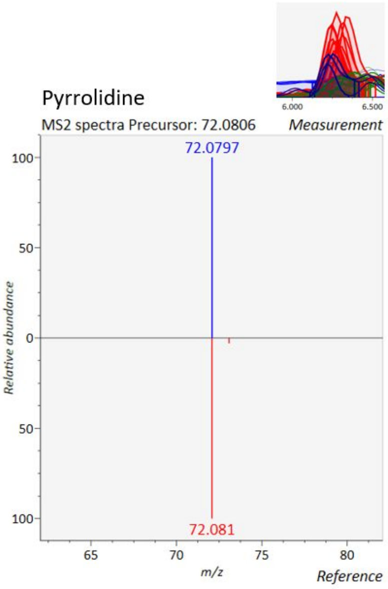

ISOLEUCINE

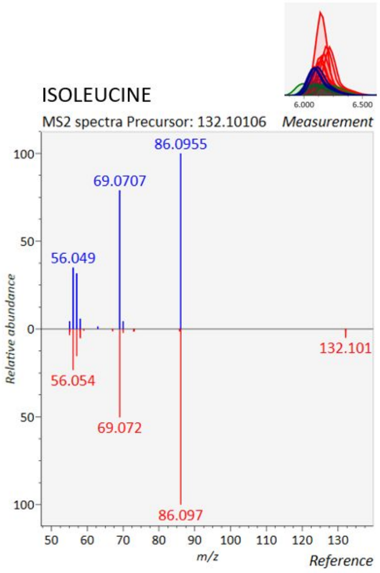

GUANOSINE 5'-MONOPHOSPHATE

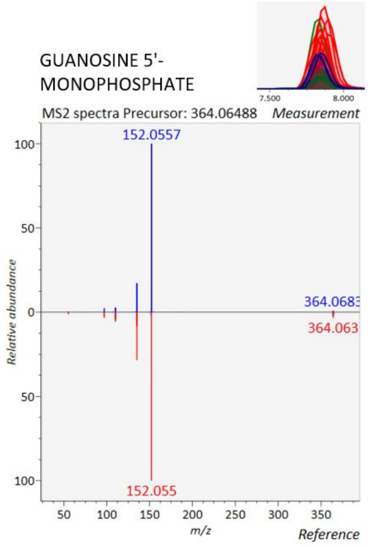

Glutamine

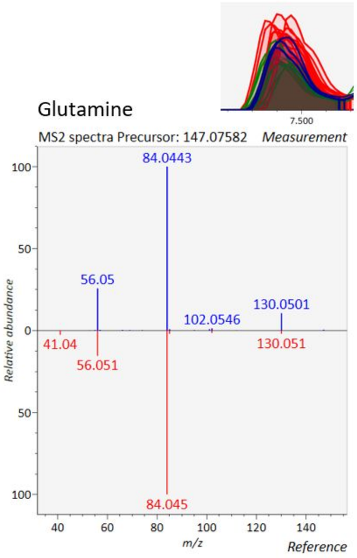

INOSINE

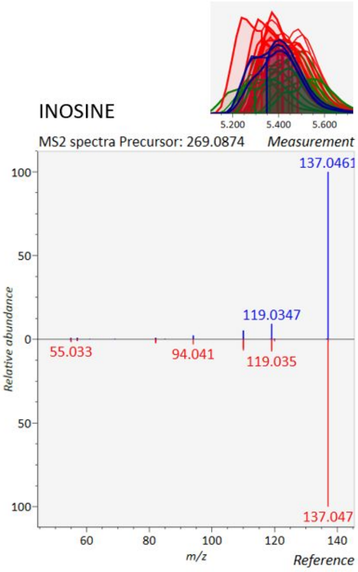

Spermidine

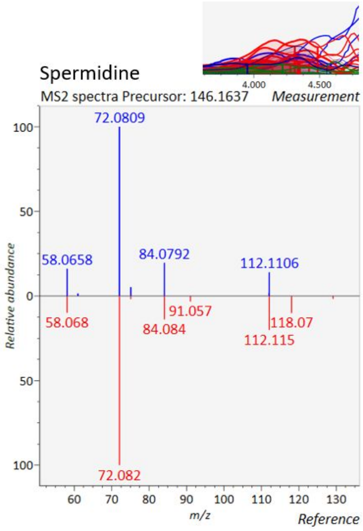

Glutathione

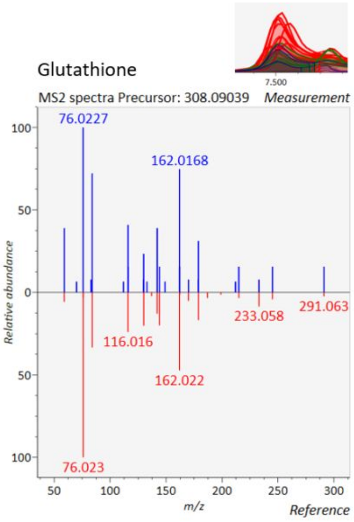

Spermidine

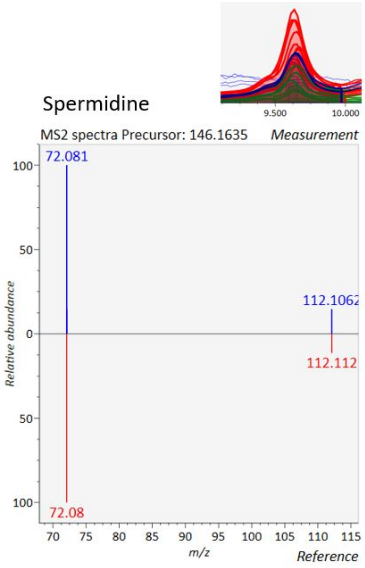

(2R)-6-methylpiperidine-2-carboxylic acid

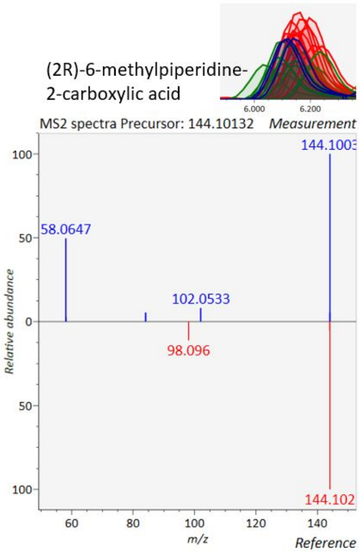

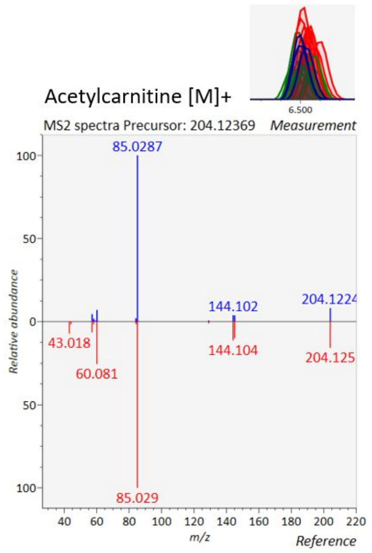
Acetylcarnitine [M]+
6.500
MS2 spectra Precursor: 204.12369
Measurement
85.0287
144.102
204.1224
43.018
60.081
144.104
204.125
85.029
Relative abundance
m/z
Reference

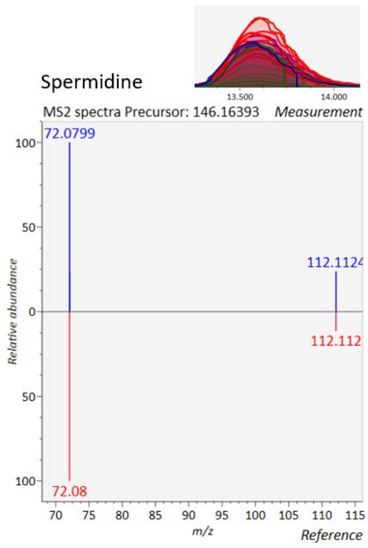
Spermidine
13.500
14.000
MS2 spectra Precursor: 146.16393
Measurement
72.0799
112.1124
112.112
72.08
Relative abundance
m/z
Reference

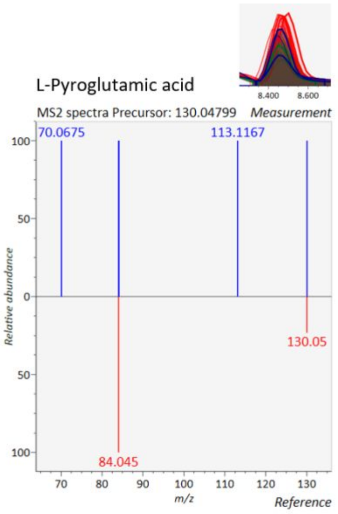
L-Pyroglutamic acid
8.400
8.600
MS2 spectra Precursor: 130.04799
Measurement
70.0675
113.1167
130.05
84.045
Relative abundance
m/z
Reference

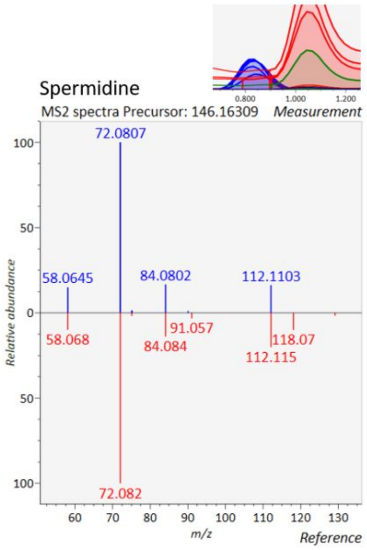
Spermidine
0.800
1.000
1.200
MS2 spectra Precursor: 146.16309
Measurement
72.0807
58.0645
84.0802
112.1103
58.068
91.057
84.084
118.07
112.115
72.082
Relative abundance
m/z
Reference

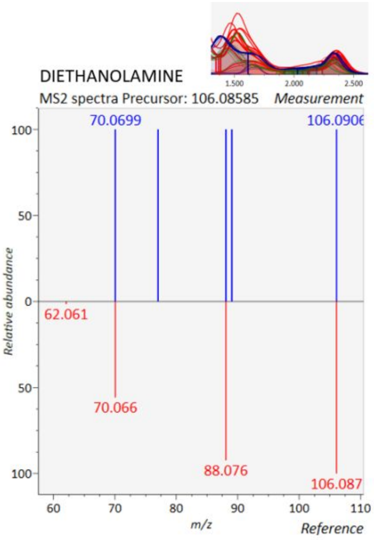
DIETHANOLAMINE
1.500
2.000
2.500
MS2 spectra Precursor: 106.08585
Measurement
70.0699
106.0906
62.061
70.066
88.076
106.087
Relative abundance
m/z
Reference

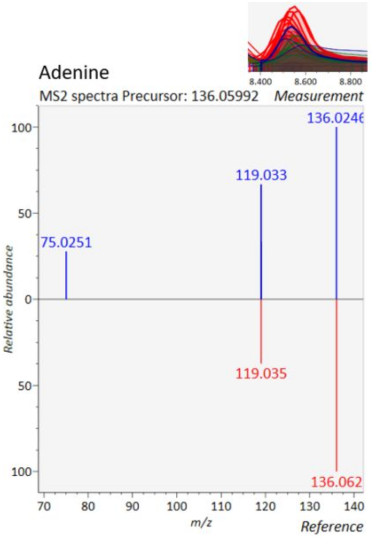
Adenine
8.400
8.600
8.800
MS2 spectra Precursor: 136.05992
Measurement
136.0246
119.033
75.0251
119.035
136.062
Relative abundance
m/z
Reference

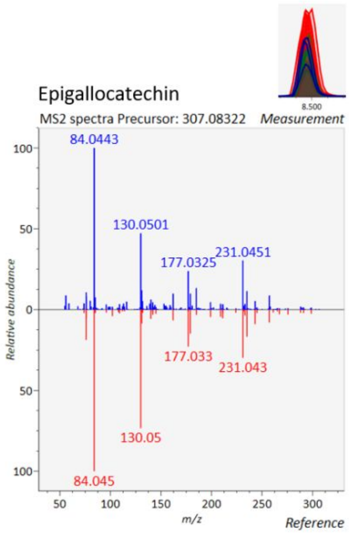
Epigallocatechin
8.500
MS2 spectra Precursor: 307.08322
Measurement
84.0443
130.0501
177.0325
231.0451
177.033
231.043
130.05
84.045
Relative abundance
m/z
Reference

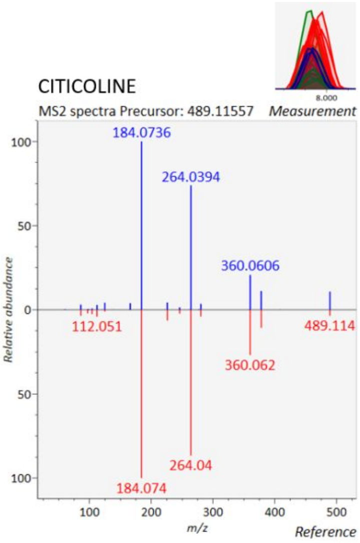
CITICOLINE
8.000
MS2 spectra Precursor: 489.11557
Measurement
184.0736
264.0394
360.0606
112.051
489.114
360.062
264.04
184.074
Relative abundance
m/z
Reference

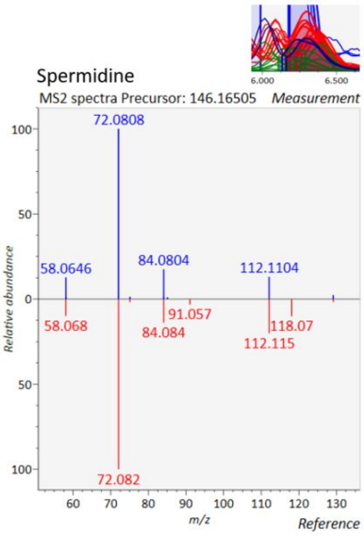
Spermidine
6.000
6.500
MS2 spectra Precursor: 146.16505
Measurement
72.0808
58.0646
84.0804
112.1104
58.068
91.057
84.084
118.07
112.115
72.082
Relative abundance
m/z
Reference

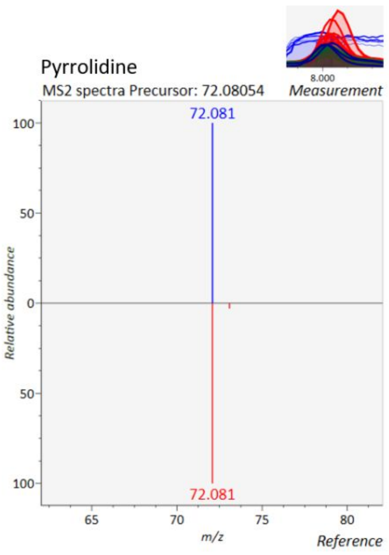
Pyrrolidine
MS2 spectra Precursor: 72.08054
Measurement
Reference
72.081
72.081
Relative abundance
m/z

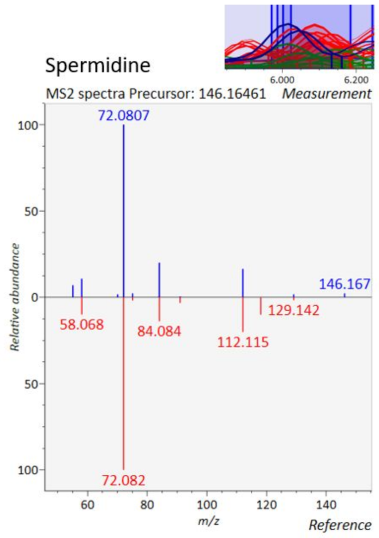
Spermidine
MS2 spectra Precursor: 146.16461
Measurement
Reference
72.0807
146.167
58.068
84.084
112.115
129.142
72.082
Relative abundance
m/z

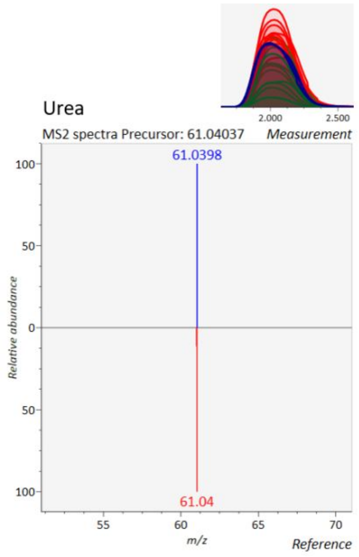
Urea
MS2 spectra Precursor: 61.04037
Measurement
Reference
61.0398
61.04
Relative abundance
m/z

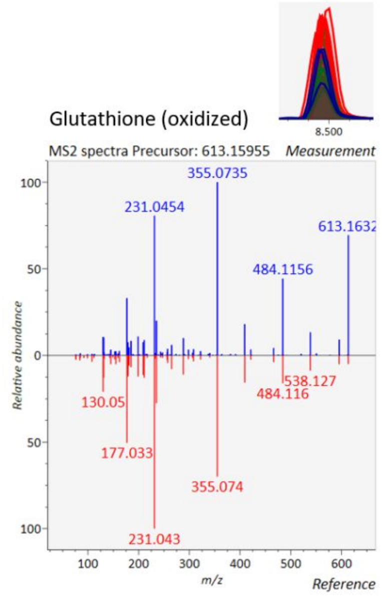
Glutathione (oxidized)
MS2 spectra Precursor: 613.15955
Measurement
Reference
355.0735
231.0454
613.1632
484.1156
130.05
538.127
484.116
177.033
355.074
231.043
Relative abundance
m/z

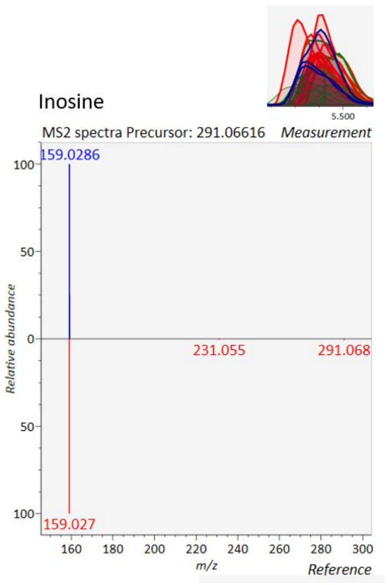
Inosine
MS2 spectra Precursor: 291.06616
Measurement
Reference
159.0286
231.055
291.068
159.027
Relative abundance
m/z

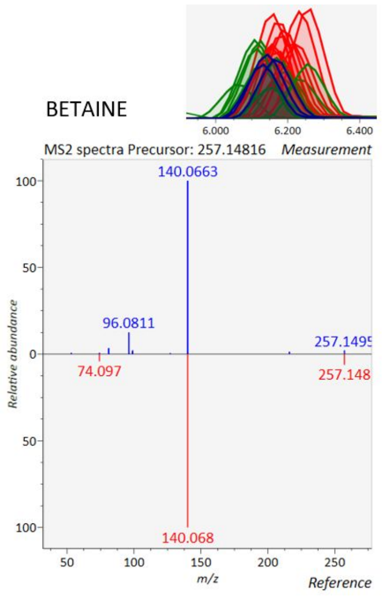
BETAINE
MS2 spectra Precursor: 257.14816
Measurement
Reference
140.0663
96.0811
257.1495
74.097
257.148
140.068
Relative abundance
m/z

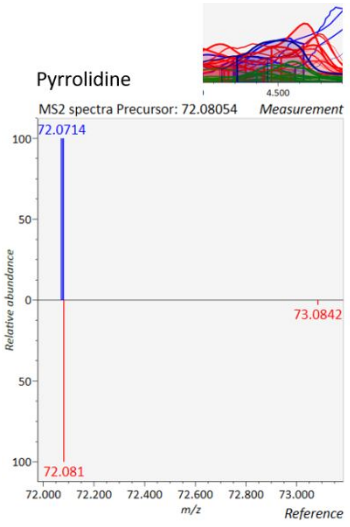
Pyrrolidine
MS2 spectra Precursor: 72.08054
Measurement
Reference
72.0714
73.0842
72.081
Relative abundance
m/z

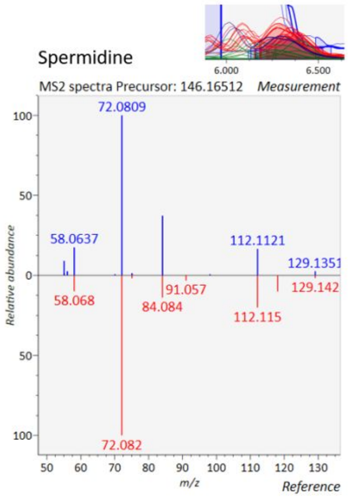
Spermidine
MS2 spectra Precursor: 146.16512
Measurement
Reference
72.0809
58.0637
112.1121
129.1351
58.068
91.057
84.084
129.142
112.115
72.082
Relative abundance
m/z

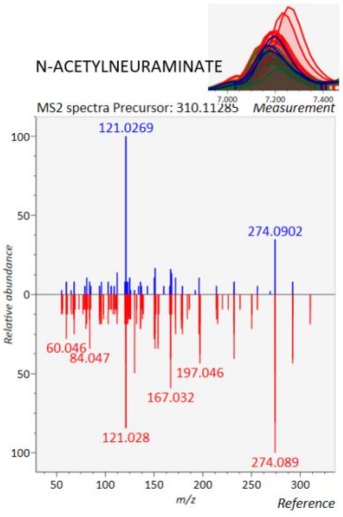
N-ACETYLNEURAMINATE
MS2 spectra Precursor: 310.11285
Measurement
Reference
121.0269
274.0902
60.046
84.047
197.046
167.032
121.028
274.089
Relative abundance
m/z

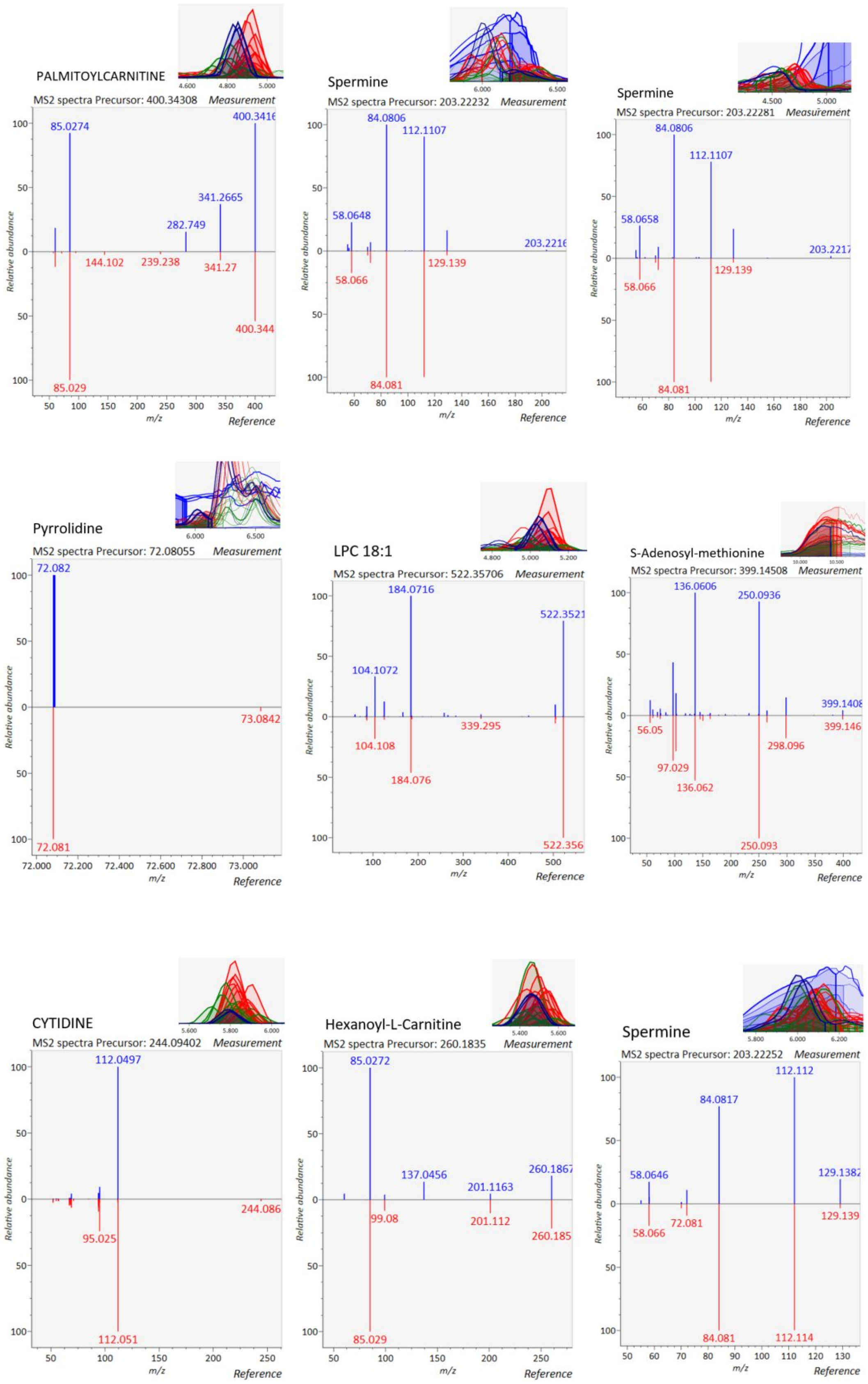
PALMITOYLCARNITINE
MS2 spectra Precursor: 400.34308 Measurement
85.0274
400.3416
341.2665
282.749
144.102
239.238
341.27
400.344
85.029
Relative abundance
m/z
Reference
Spermine
MS2 spectra Precursor: 203.22232 Measurement
84.0806
112.1107
58.0648
203.2216
129.139
58.066
84.081
Spermine
MS2 spectra Precursor: 203.22281 Measurement
84.0806
112.1107
58.0658
203.2217
129.139
58.066
84.081
Pyrrolidine
MS2 spectra Precursor: 72.08055 Measurement
72.082
73.0842
72.081
LPC 18:1
MS2 spectra Precursor: 522.35706 Measurement
184.0716
522.3521
104.1072
104.108
339.295
184.076
522.356
S-Adenosyl-methionine
MS2 spectra Precursor: 399.14508 Measurement
136.0606
250.0936
399.1408
56.05
399.146
298.096
97.029
136.062
250.093
CYTIDINE
MS2 spectra Precursor: 244.09402 Measurement
112.0497
244.086
95.025
112.051
Hexanoyl-L-Carnitine
MS2 spectra Precursor: 260.1835 Measurement
85.0272
137.0456
201.1163
260.1867
99.08
201.112
260.185
85.029
Spermine
MS2 spectra Precursor: 203.22252 Measurement
112.112
84.0817
58.0646
129.1382
58.066
72.081
129.139
84.081
112.114

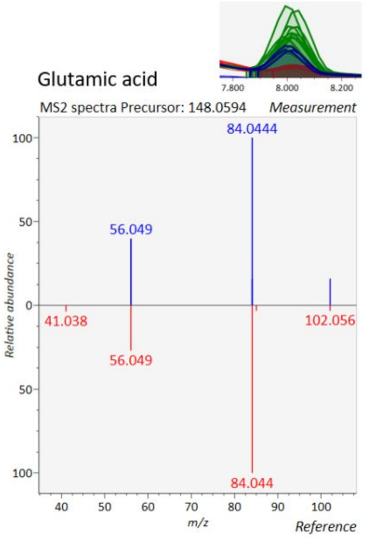
Glutamic acid
MS2 spectra Precursor: 148.0594
Measurement
84.0444
56.049
41.038
56.049
102.056
84.044
Relative abundance
m/z
Reference

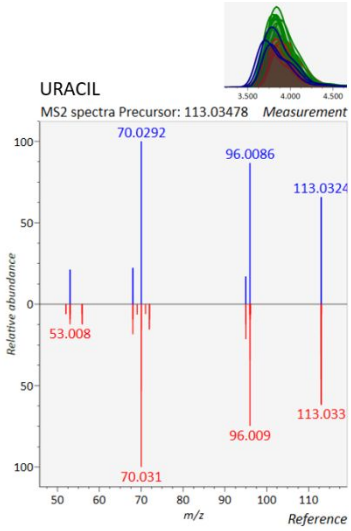
URACIL
MS2 spectra Precursor: 113.03478
Measurement
70.0292
96.0086
113.0324
53.008
113.033
96.009
70.031
Relative abundance
m/z
Reference

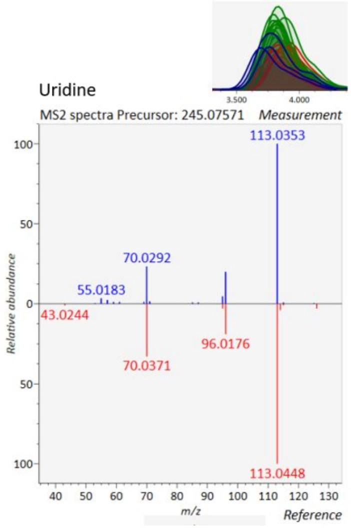
Uridine
MS2 spectra Precursor: 245.07571
Measurement
113.0353
70.0292
55.0183
43.0244
96.0176
70.0371
113.0448
Relative abundance
m/z
Reference

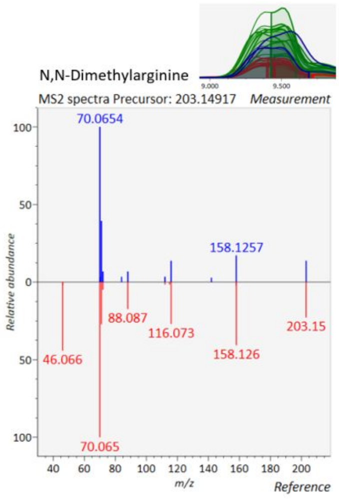
N,N-Dimethylarginine
MS2 spectra Precursor: 203.14917
Measurement
70.0654
158.1257
88.087
116.073
203.15
46.066
158.126
70.065
Relative abundance
m/z
Reference

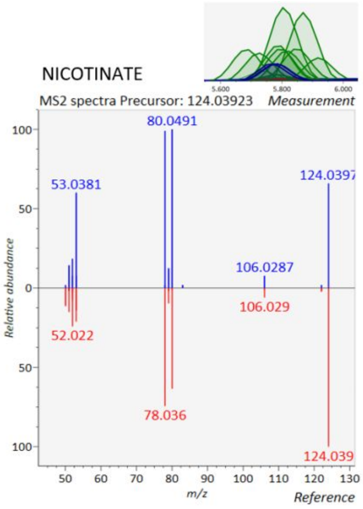
NICOTINATE
MS2 spectra Precursor: 124.03923
Measurement
80.0491
53.0381
124.0397
106.0287
106.029
52.022
78.036
124.039
Relative abundance
m/z
Reference

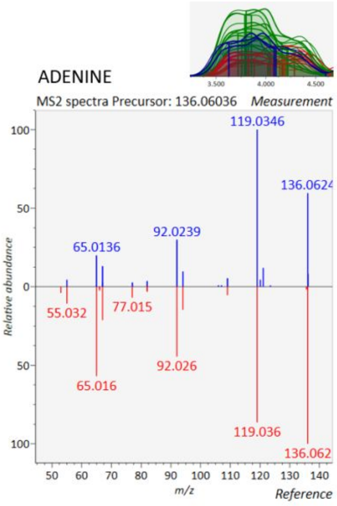
ADENINE
MS2 spectra Precursor: 136.06036
Measurement
119.0346
136.0624
92.0239
65.0136
55.032
77.015
92.026
65.016
119.036
136.062
Relative abundance
m/z
Reference

1-METHYLADENOSINE

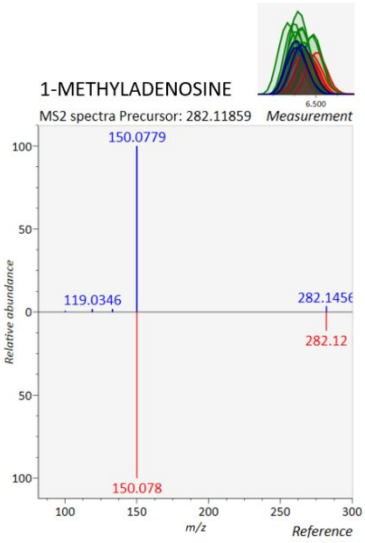

N,N,N-TRIMETHYLLYSINE

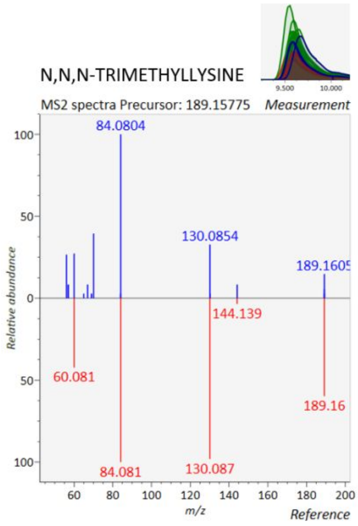

Ornithine

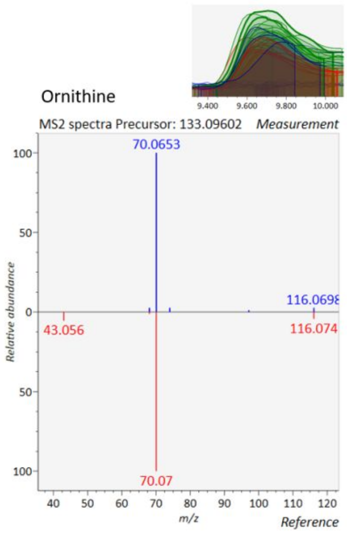

Tubuloside A

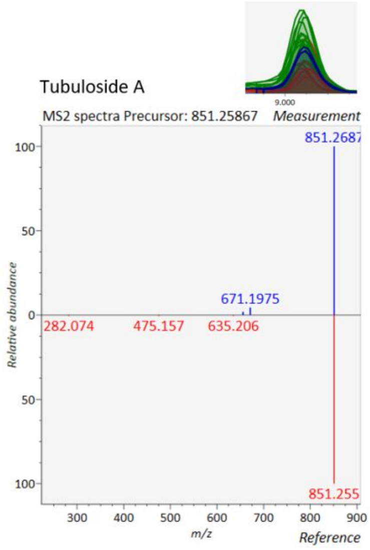

Thiazolidine-4-carboxylic acid

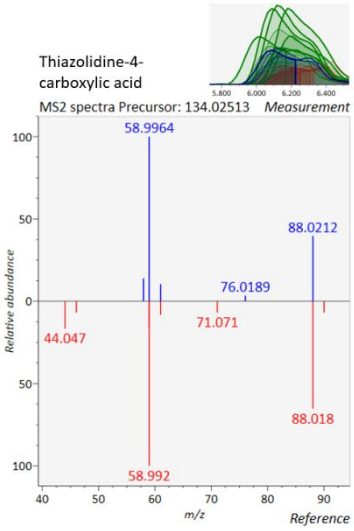

Ethylbutanoate

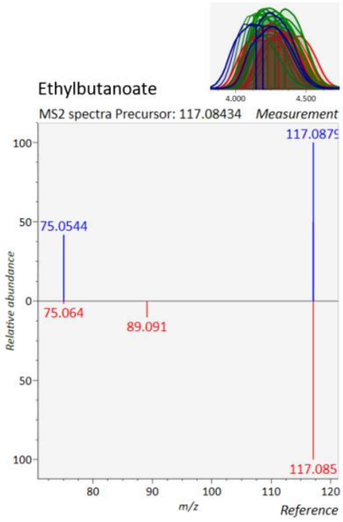

gamma-Glutamylglutamine

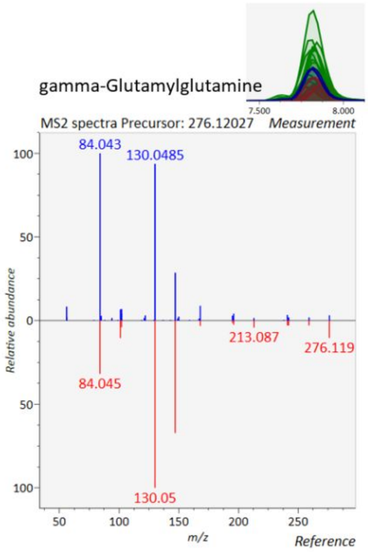

PYRIDOXAL

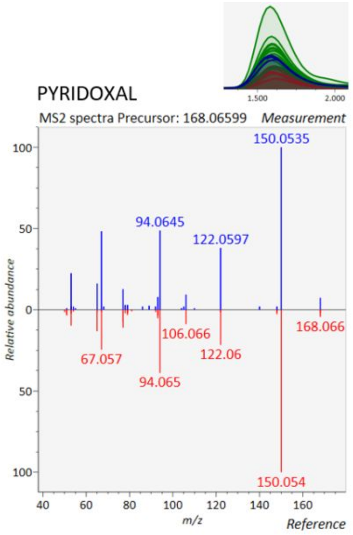

Melezitose

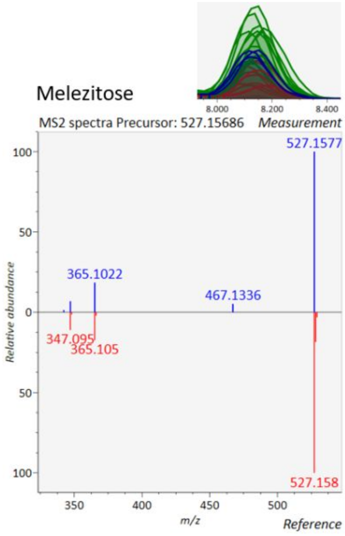

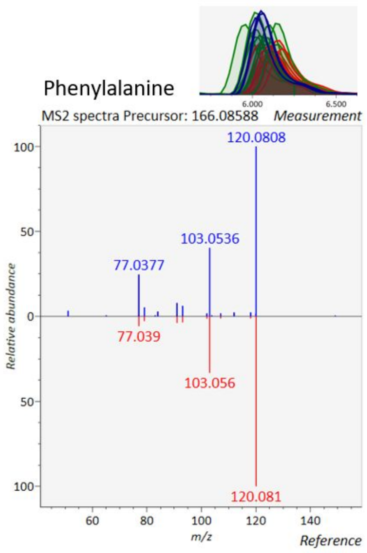
Phenylalanine
MS2 spectra Precursor: 166.08588 Measurement
120.0808
103.0536
77.0377
77.039
103.056
120.081
Relative abundance
m/z
Reference

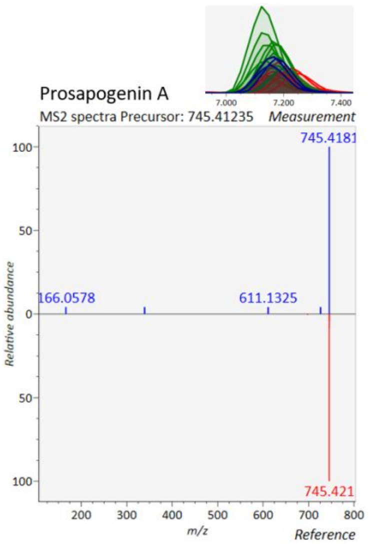
Prosapogenin A
MS2 spectra Precursor: 745.41235 Measurement
745.4181
166.0578
611.1325
745.421
Relative abundance
m/z
Reference

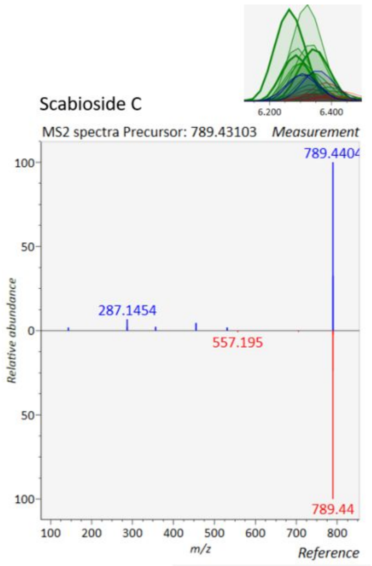
Scabioside C
MS2 spectra Precursor: 789.43103 Measurement
789.4404
287.1454
557.195
789.44
Relative abundance
m/z
Reference

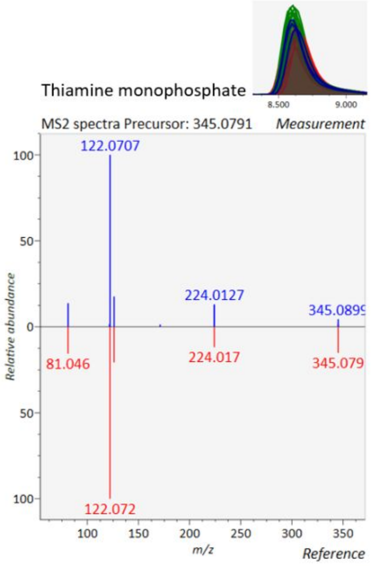
Thiamine monophosphate
MS2 spectra Precursor: 345.0791 Measurement
122.0707
224.0127
345.0899
81.046
224.017
345.079
122.072
Relative abundance
m/z
Reference

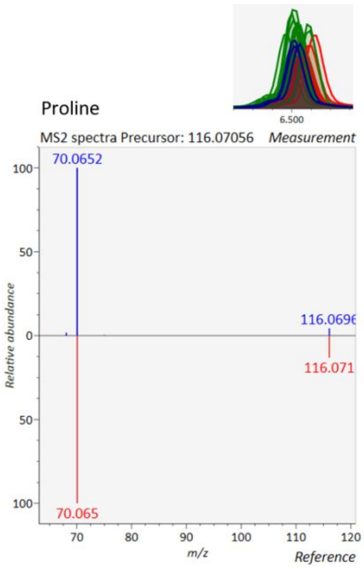
Proline
MS2 spectra Precursor: 116.07056 Measurement
70.0652
116.0696
116.071
70.065
Relative abundance
m/z
Reference

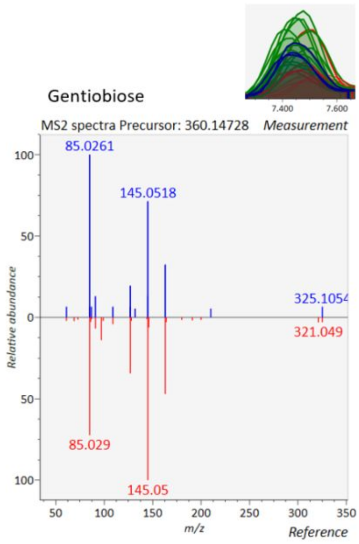
Gentiobiose
MS2 spectra Precursor: 360.14728 Measurement
85.0261
145.0518
325.1054
321.049
85.029
145.05
Relative abundance
m/z
Reference

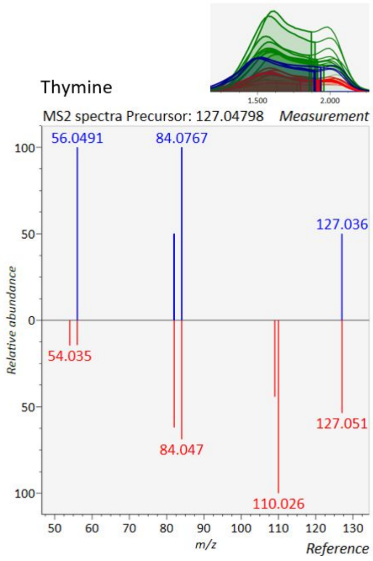
Thymine
MS2 spectra Precursor: 127.04798 Measurement
56.0491
84.0767
127.036
54.035
127.051
84.047
110.026
Relative abundance
m/z
Reference

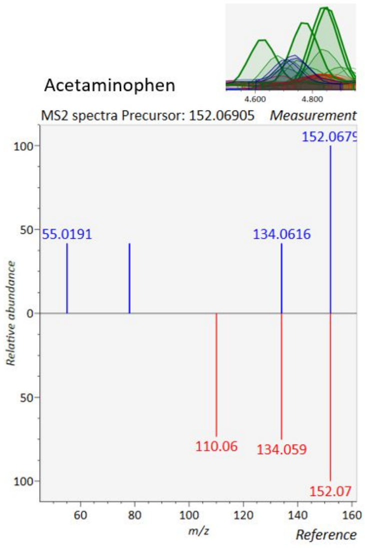
Acetaminophen
MS2 spectra Precursor: 152.06905 Measurement
152.0679
55.0191
134.0616
110.06
134.059
152.07
Relative abundance
m/z
Reference

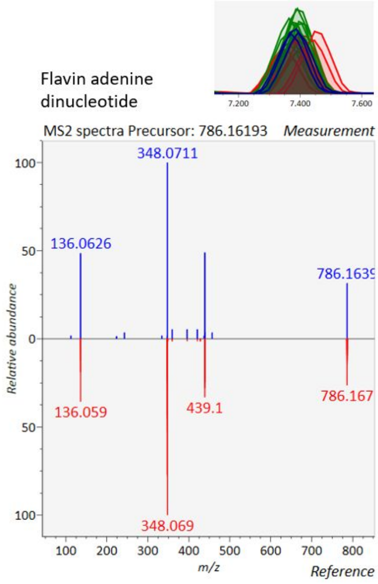
Flavin adenine dinucleotide
MS2 spectra Precursor: 786.16193 Measurement
348.0711
136.0626
786.1639
136.059
439.1
786.167
348.069
Relative abundance
m/z
Reference

Glutamine

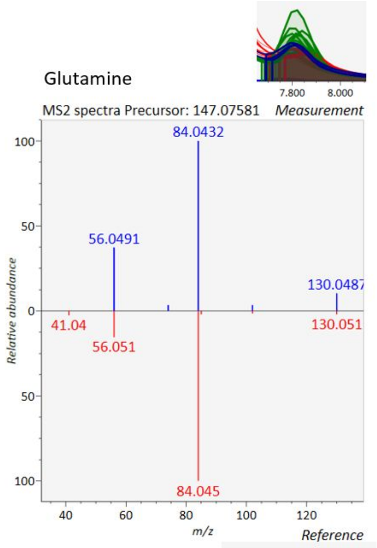

SUCROSE

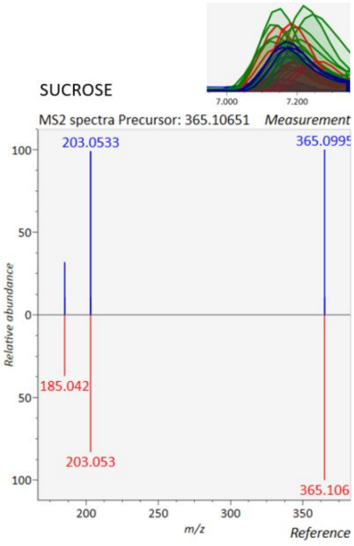

Choline

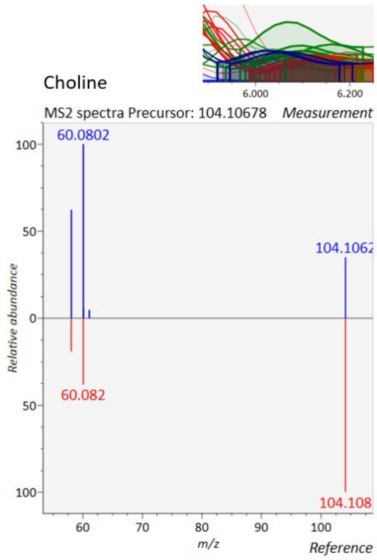

3-Formylindole

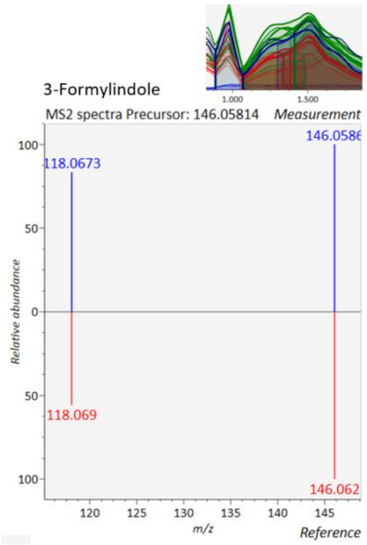

D-Mannose-6-phosphate

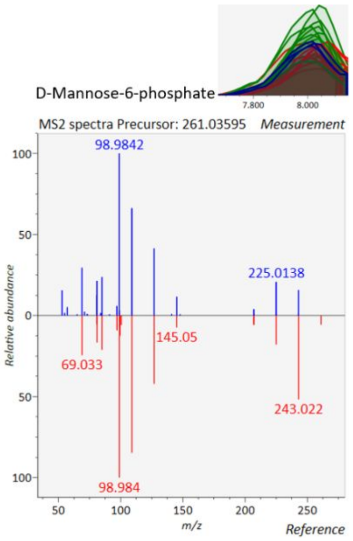

Alanine betaine

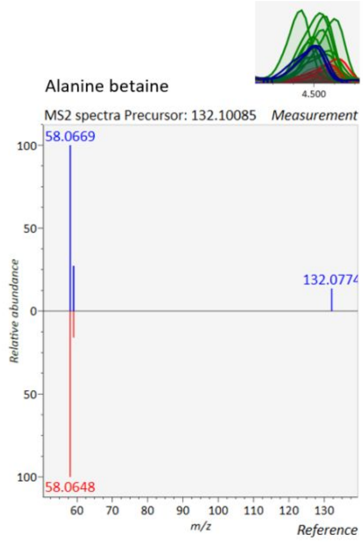

Norharman

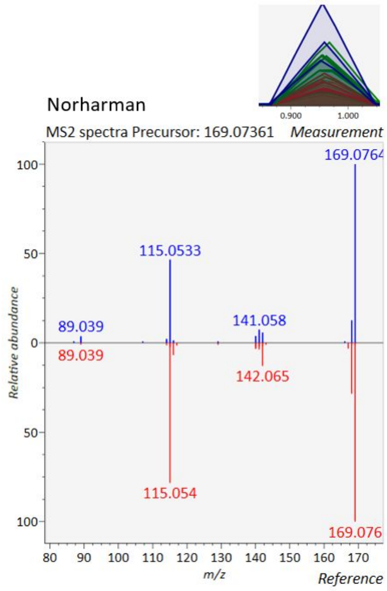

CITRULLINE

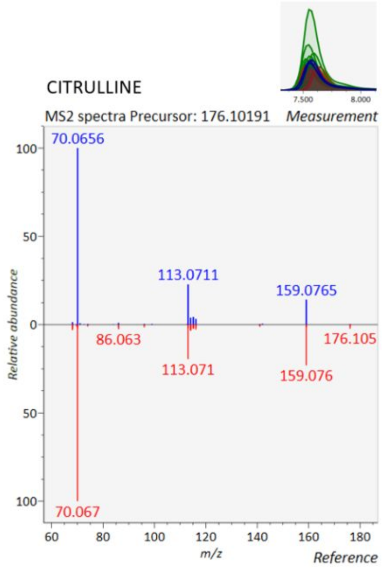

2-hydroxyquinoline

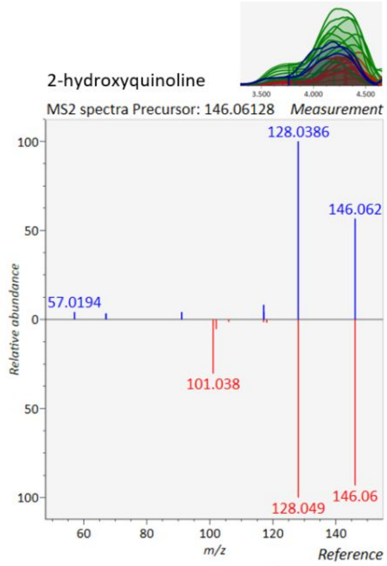

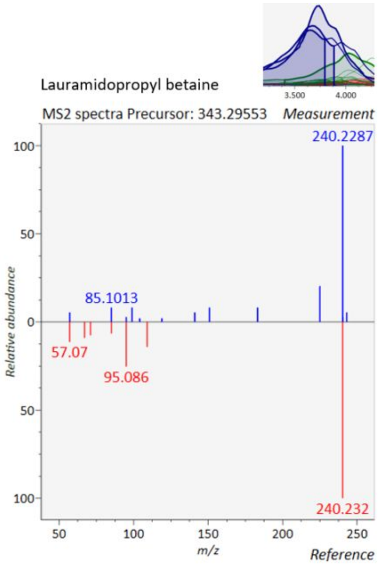
Lauramidopropyl betaine
MS2 spectra Precursor: 343.29553
Measurement
240.2287
85.1013
57.07
95.086
240.232
Relative abundance
m/z
Reference

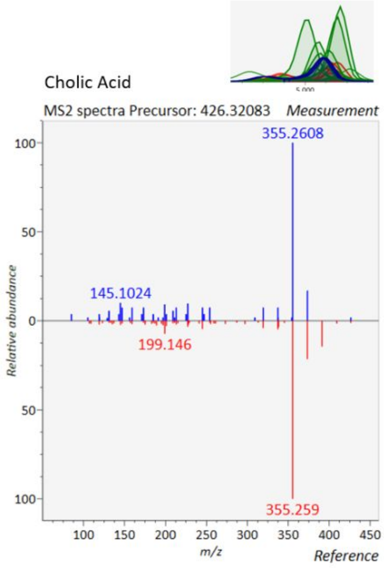
Cholic Acid
MS2 spectra Precursor: 426.32083
Measurement
355.2608
145.1024
199.146
355.259
Relative abundance
m/z
Reference

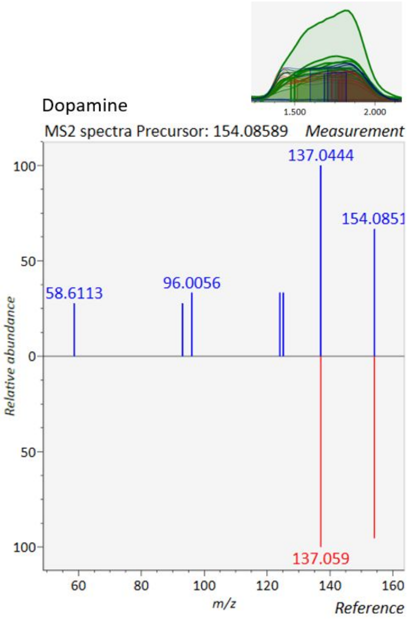
Dopamine
MS2 spectra Precursor: 154.08589
Measurement
137.0444
154.0851
58.6113
96.0056
137.059
Relative abundance
m/z
Reference

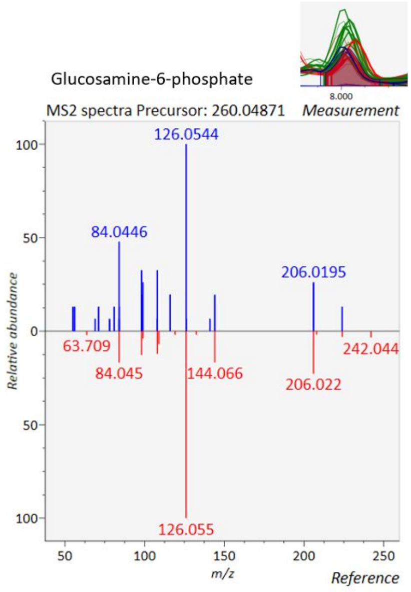
Glucosamine-6-phosphate
MS2 spectra Precursor: 260.04871
Measurement
126.0544
84.0446
206.0195
63.709
84.045
144.066
206.022
242.044
126.055
Relative abundance
m/z
Reference

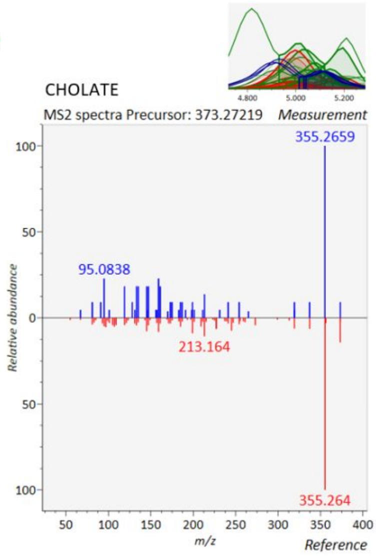
CHOLATE
MS2 spectra Precursor: 373.27219
Measurement
355.2659
95.0838
213.164
355.264
Relative abundance
m/z
Reference

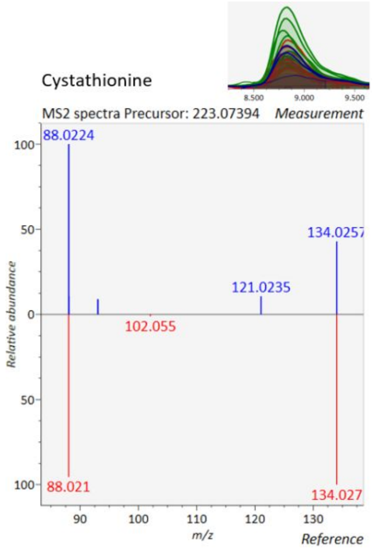
Cystathionine
MS2 spectra Precursor: 223.07394
Measurement
88.0224
134.0257
121.0235
102.055
88.021
134.027
Relative abundance
m/z
Reference

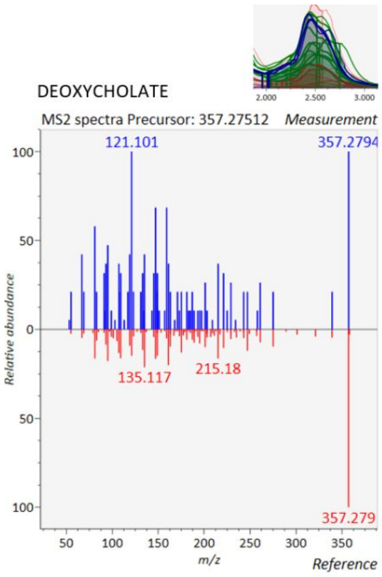
DEOXYCHOLATE
MS2 spectra Precursor: 357.27512
Measurement
121.101
357.2794
135.117
215.18
357.279
Relative abundance
m/z
Reference

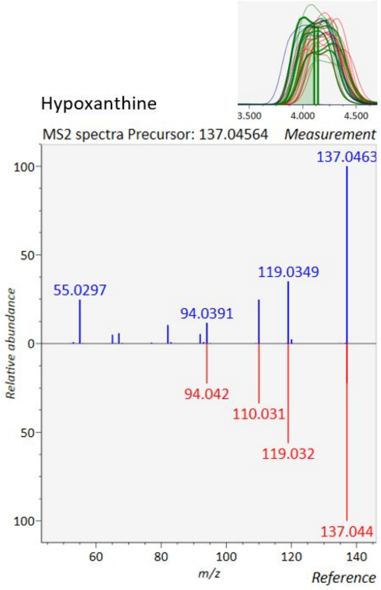
Hypoxanthine
MS2 spectra Precursor: 137.04564
Measurement
137.0463
55.0297
119.0349
94.0391
94.042
110.031
119.032
137.044
Relative abundance
m/z
Reference

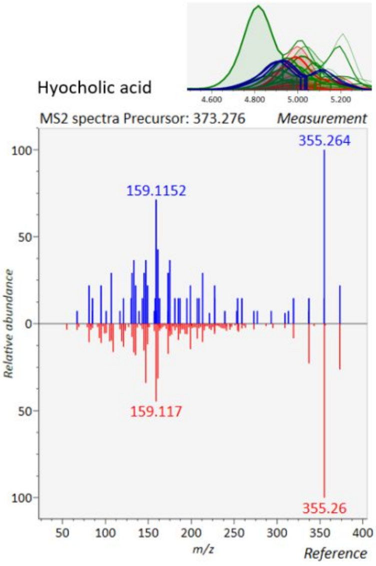
Hyocholic acid
MS2 spectra Precursor: 373.276
Measurement
355.264
159.1152
159.117
355.26
Relative abundance
m/z
Reference

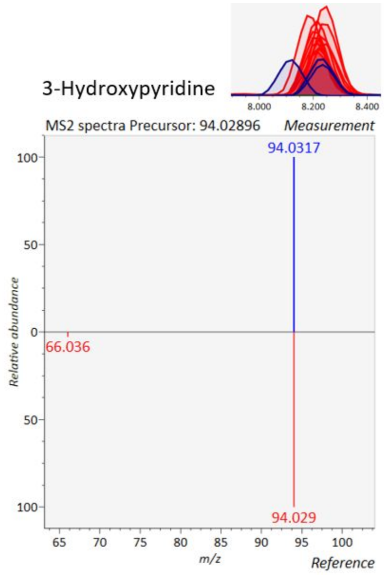
3-Hydroxypyridine
MS2 spectra Precursor: 94.02896
Measurement
94.0317
66.036
94.029
Relative abundance
m/z
Reference

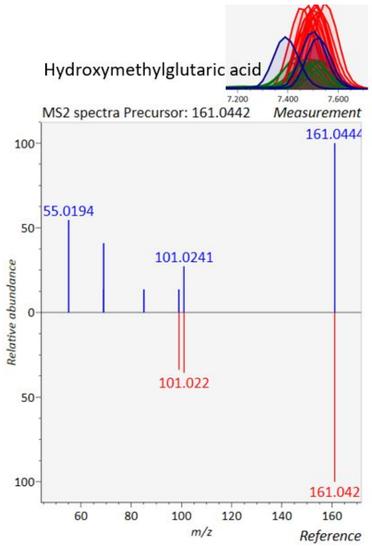
Hydroxymethylglutaric acid
MS2 spectra Precursor: 161.0442
Measurement
161.0444
55.0194
101.0241
101.022
161.042
Relative abundance
m/z
Reference

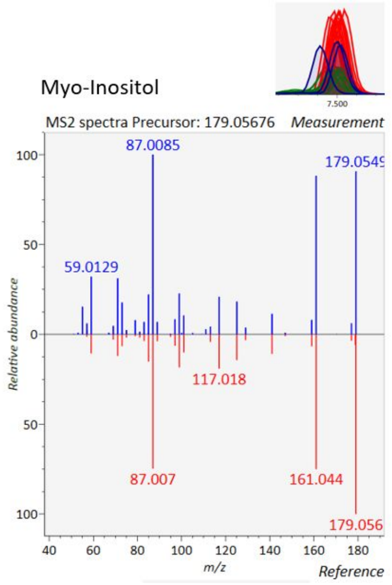
Myo-Inositol
MS2 spectra Precursor: 179.05676
Measurement
87.0085
179.0549
59.0129
117.018
87.007
161.044
179.056
Relative abundance
m/z
Reference

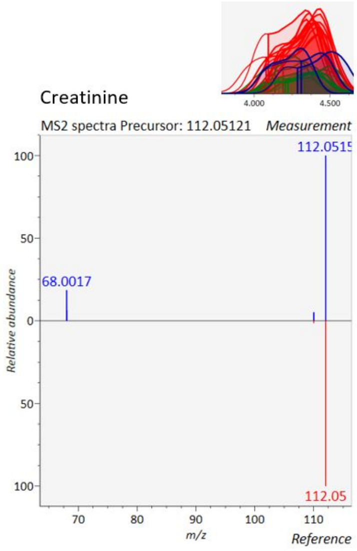
Creatinine
MS2 spectra Precursor: 112.05121
Measurement
112.0515
68.0017
112.05
Relative abundance
m/z
Reference

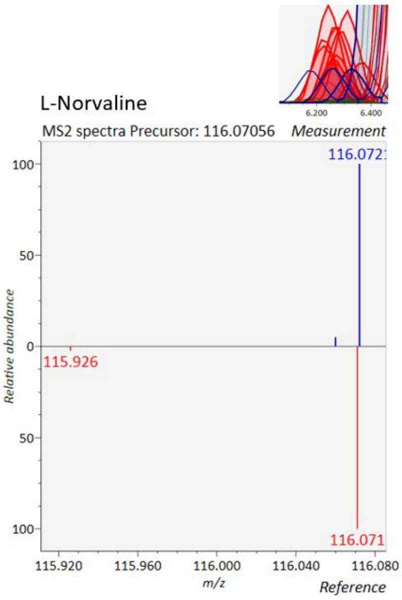
L-Norvaline
MS2 spectra Precursor: 116.07056
Measurement
116.0721
115.926
116.071
Relative abundance
m/z
Reference

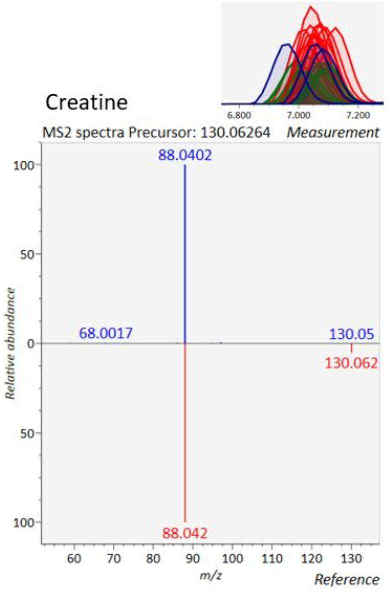
Creatine
MS2 spectra Precursor: 130.06264
Measurement
88.0402
68.0017
130.05
130.062
88.042
Relative abundance
m/z
Reference

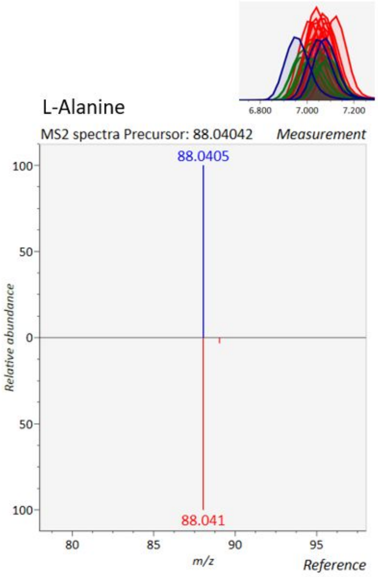
L-Alanine
MS2 spectra Precursor: 88.04042
Measurement
88.0405
88.041
Relative abundance
m/z
Reference

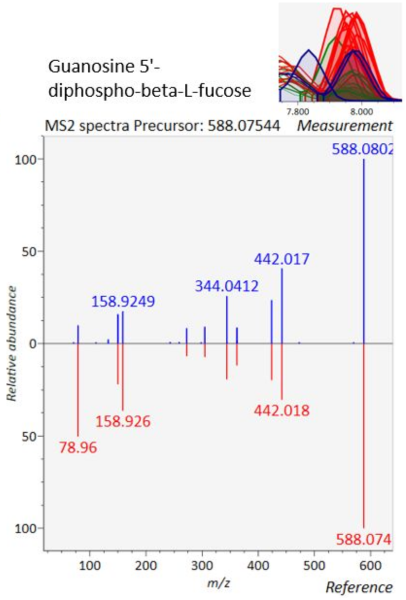
Guanosine 5'-diphospho-beta-L-fucose
MS2 spectra Precursor: 588.07544
Measurement
588.0802
442.017
344.0412
158.9249
158.926
78.96
442.018
588.074
Relative abundance
m/z
Reference

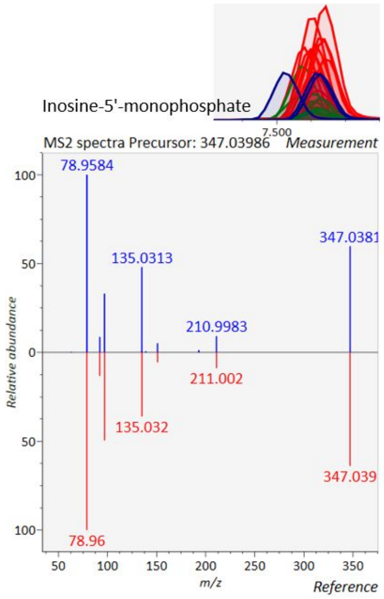
Inosine-5'-monophosphate
MS2 spectra Precursor: 347.03986
Measurement
78.9584
347.0381
135.0313
210.9983
211.002
135.032
347.039
78.96
Relative abundance
m/z
Reference

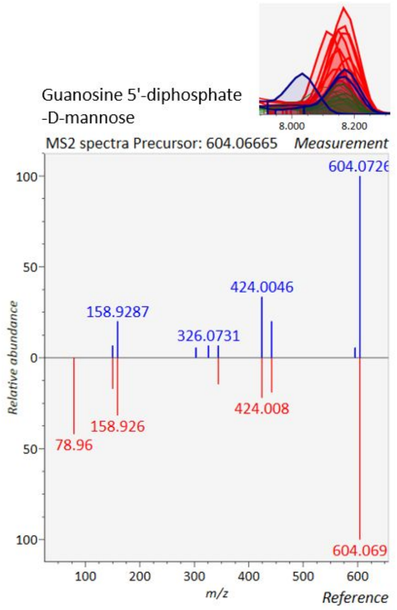
Guanosine 5'-diphosphate
-D-mannose
8.000
8.200
MS2 spectra Precursor: 604.06665
Measurement
604.0726
158.9287
326.0731
424.0046
78.96
158.926
424.008
604.069
Relative abundance
m/z
Reference

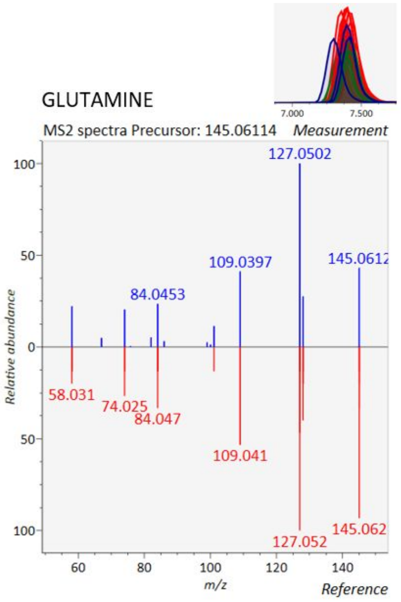
GLUTAMINE
7.000
7.500
MS2 spectra Precursor: 145.06114
Measurement
127.0502
109.0397
145.0612
84.0453
58.031
74.025
84.047
109.041
127.052
145.062
Relative abundance
m/z
Reference

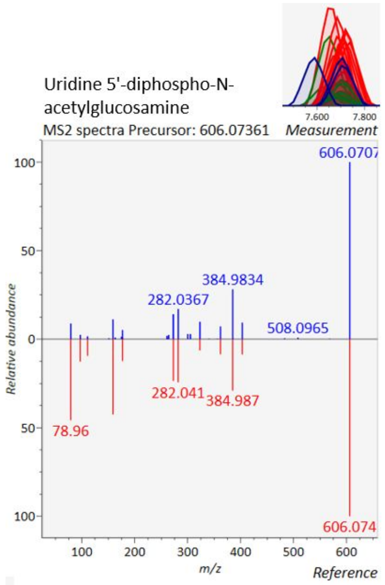
Uridine 5'-diphospho-N-
acetylglucosamine
7.600
7.800
MS2 spectra Precursor: 606.07361
Measurement
606.0707
384.9834
282.0367
508.0965
78.96
282.041
384.987
606.074
Relative abundance
m/z
Reference

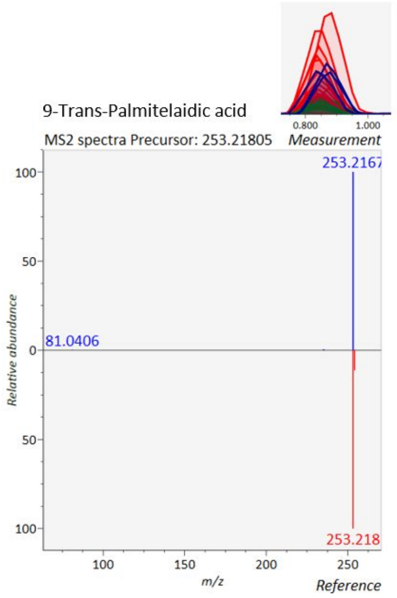
9-Trans-Palmitelaidic acid
0.800
1.000
MS2 spectra Precursor: 253.21805
Measurement
253.2167
81.0406
253.218
Relative abundance
m/z
Reference

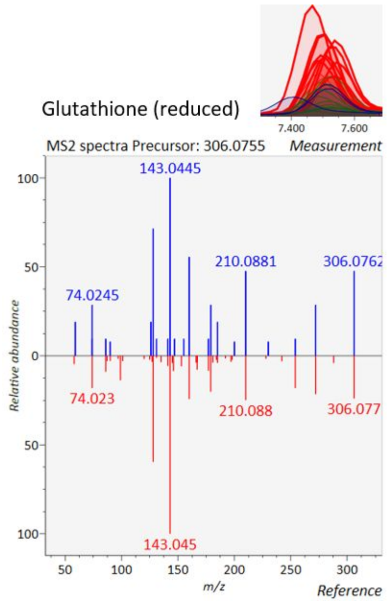
Glutathione (reduced)
7.400
7.600
MS2 spectra Precursor: 306.0755
Measurement
143.0445
210.0881
306.0762
74.0245
74.023
210.088
306.077
143.045
Relative abundance
m/z
Reference

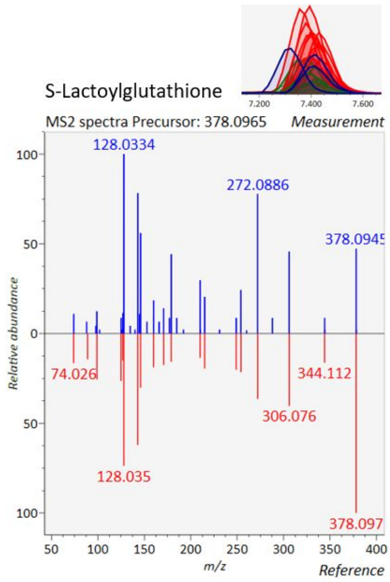
S-Lactoylglutathione
7.200
7.400
7.600
MS2 spectra Precursor: 378.0965
Measurement
128.0334
272.0886
378.0945
74.026
344.112
306.076
128.035
378.097
Relative abundance
m/z
Reference

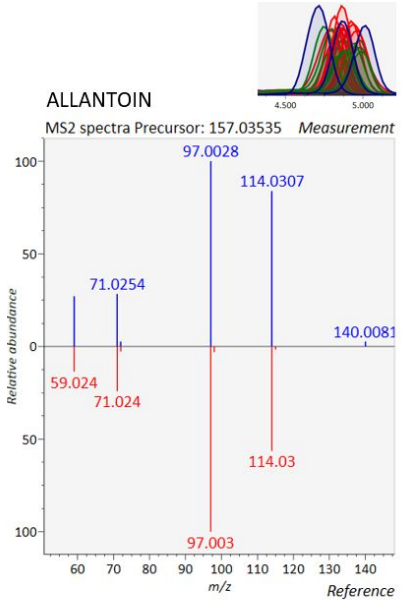
ALLANTOIN
4.500
5.000
MS2 spectra Precursor: 157.03535
Measurement
97.0028
114.0307
71.0254
140.0081
59.024
71.024
114.03
97.003
Relative abundance
m/z
Reference

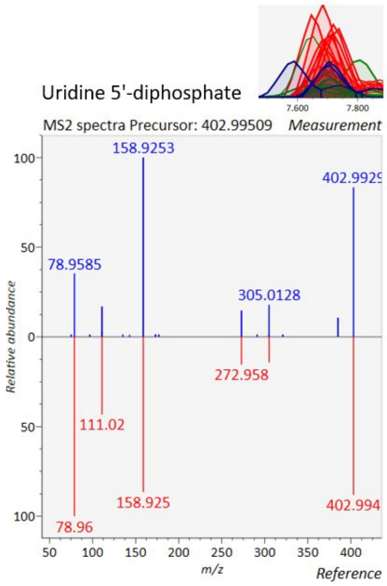
Uridine 5'-diphosphate
7.600
7.800
MS2 spectra Precursor: 402.99509
Measurement
158.9253
402.9929
78.9585
305.0128
272.958
111.02
158.925
402.994
78.96
Relative abundance
m/z
Reference

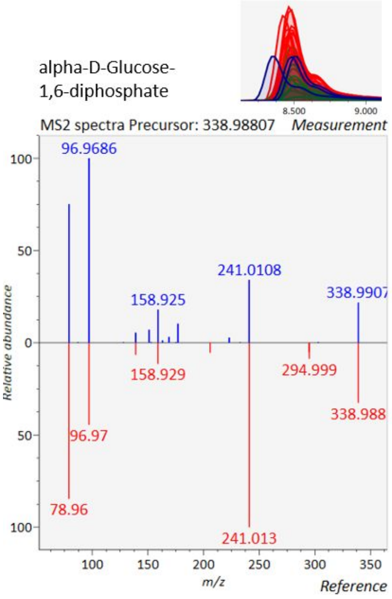
alpha-D-Glucose-
1,6-diphosphate
8.500
9.000
MS2 spectra Precursor: 338.98807
Measurement
96.9686
241.0108
338.9907
158.925
158.929
294.999
338.988
96.97
78.96
241.013
Relative abundance
m/z
Reference

D-Fructose-6-phosphate

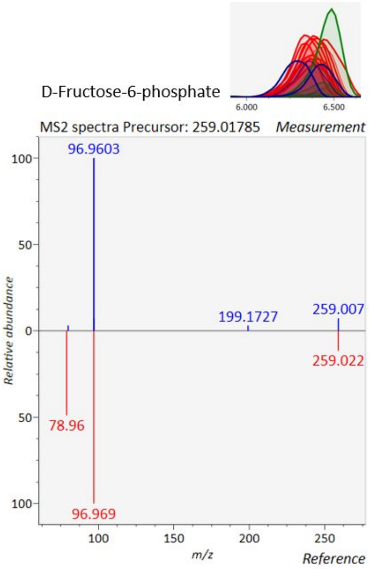

Inosine

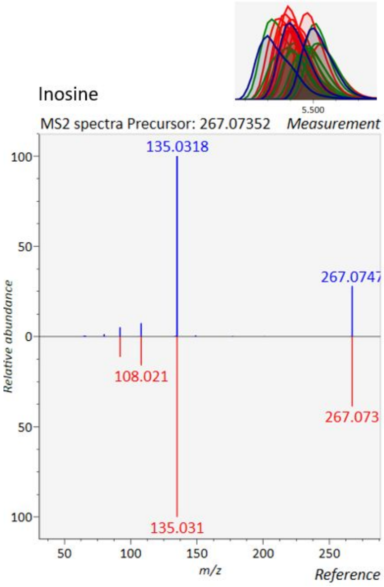

Guanosine 5'-monophosphate

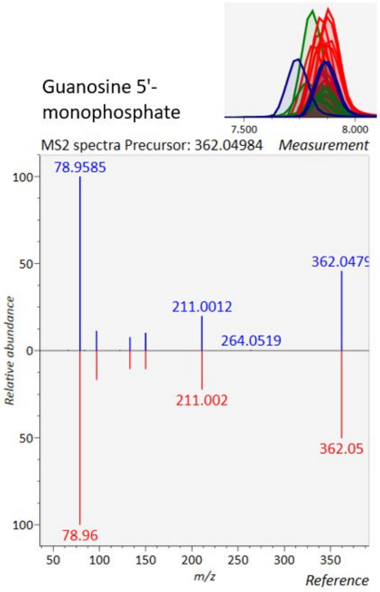

CDP-ETHANOLAMINE

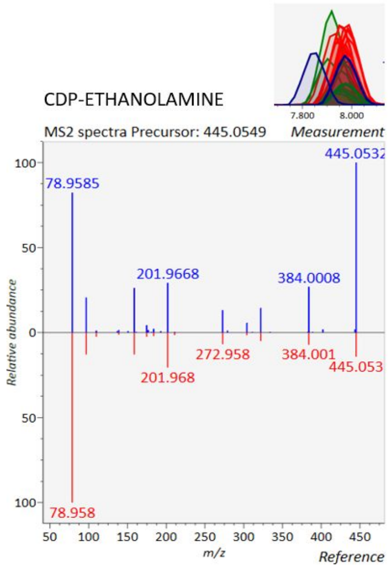

ADENOSINE 3',5'-DIPHOSPHATE

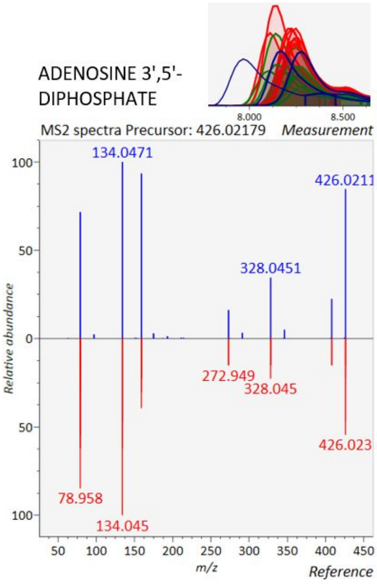

L-Anserine

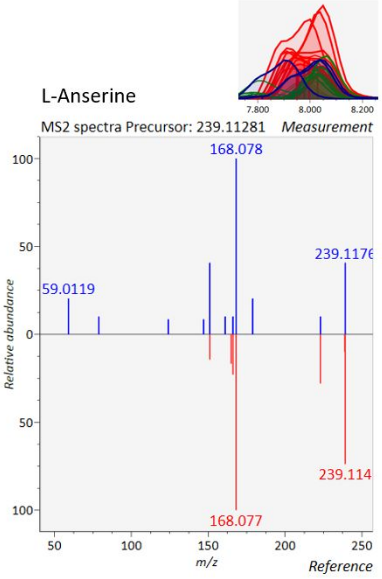

2-AMINOETHYL DIHYDROGEN PHOSPHATE

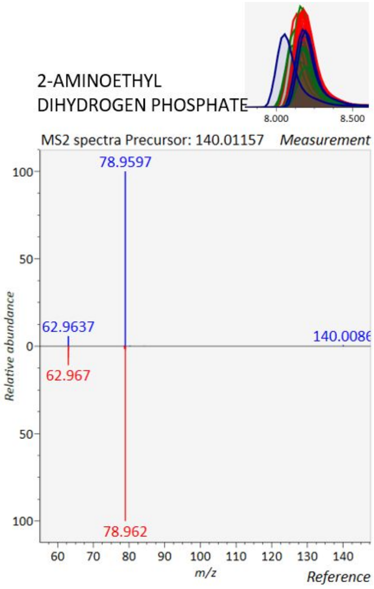

LPE 18:1

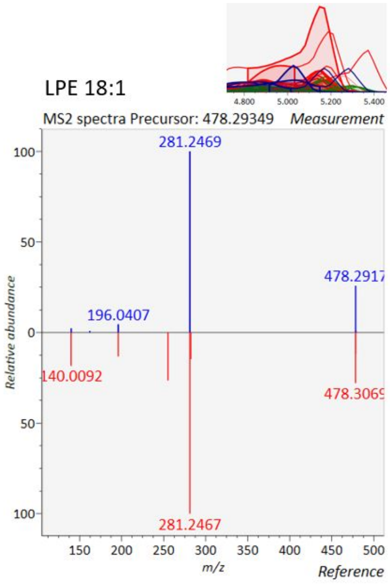

Uridine 5'-diphosphoglucuronic acid

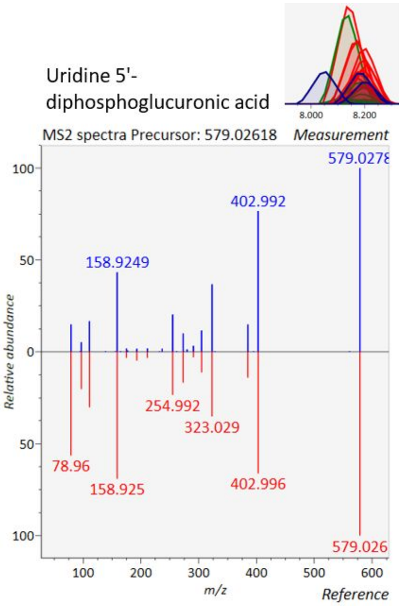

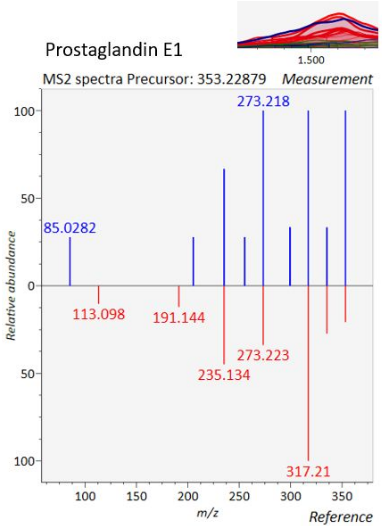
Prostaglandin E1
MS2 spectra Precursor: 353.22879
Measurement
Reference
Relative abundance
m/z
85.0282
273.218
113.098
191.144
235.134
273.223
317.21

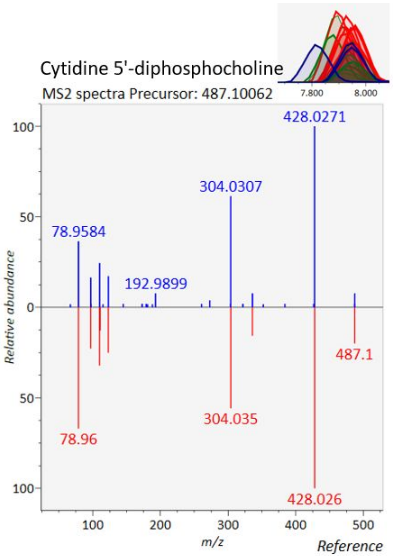
Cytidine 5'-diphosphocholine
MS2 spectra Precursor: 487.10062
Measurement
Reference
Relative abundance
m/z
428.0271
304.0307
78.9584
192.9899
78.96
304.035
428.026
487.1

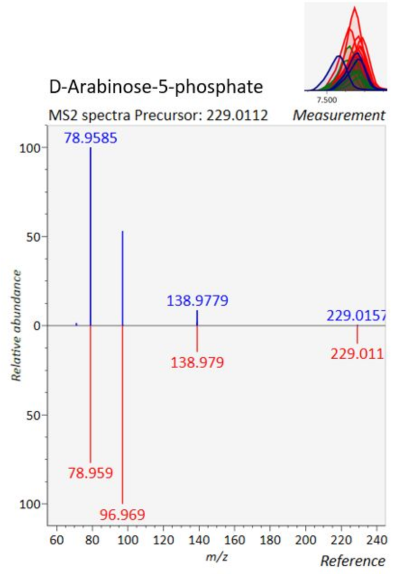
D-Arabinose-5-phosphate
MS2 spectra Precursor: 229.0112
Measurement
Reference
Relative abundance
m/z
78.9585
138.9779
229.0157
138.979
229.011
78.959
96.969

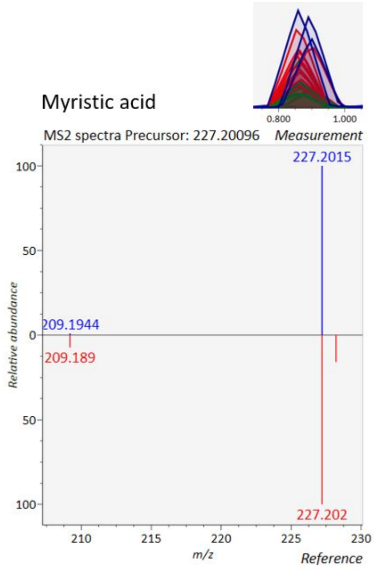
Myristic acid
MS2 spectra Precursor: 227.20096
Measurement
Reference
Relative abundance
m/z
227.2015
209.1944
209.189
227.202

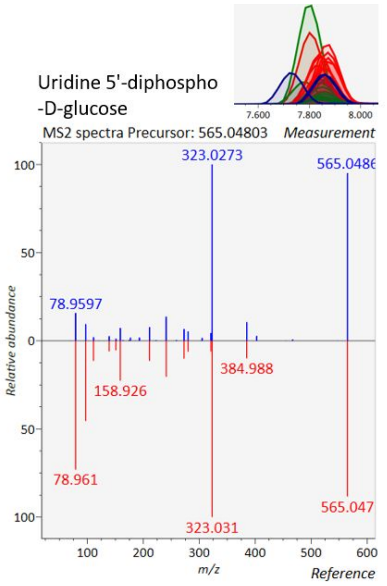
Uridine 5'-diphospho
-D-glucose
MS2 spectra Precursor: 565.04803
Measurement
Reference
Relative abundance
m/z
323.0273
78.9597
158.926
384.988
78.961
323.031
565.047

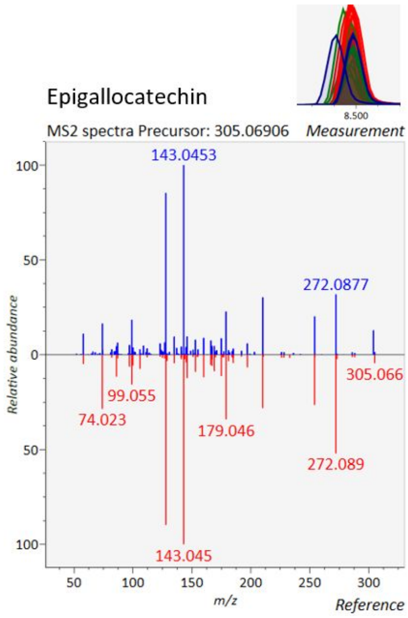
Epigallocatechin
MS2 spectra Precursor: 305.06906
Measurement
Reference
Relative abundance
m/z
143.0453
272.0877
305.066
99.055
74.023
179.046
272.089
143.045

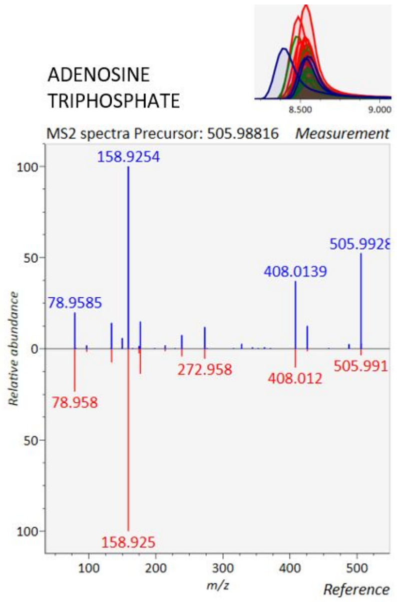
ADENOSINE
TRIPHOSPHATE
MS2 spectra Precursor: 505.98816
Measurement
Reference
Relative abundance
m/z
158.9254
408.0139
78.9585
78.958
272.958
408.012
505.991
158.925

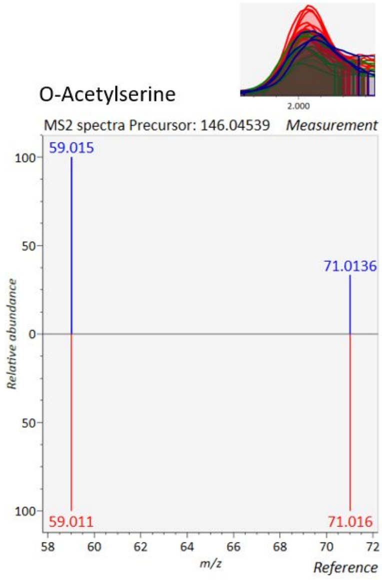
O-Acetylserine
MS2 spectra Precursor: 146.04539
Measurement
Reference
Relative abundance
m/z
59.015
71.0136
59.011
71.016

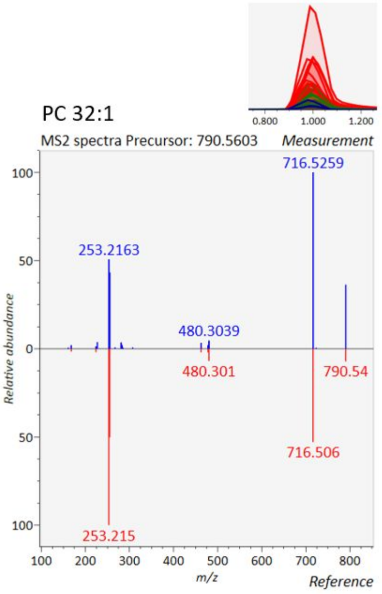
PC 32:1
MS2 spectra Precursor: 790.5603
Measurement
Reference
Relative abundance
m/z
716.5259
253.2163
480.3039
480.301
790.54
716.506
253.215

Glutathione (oxidized form)

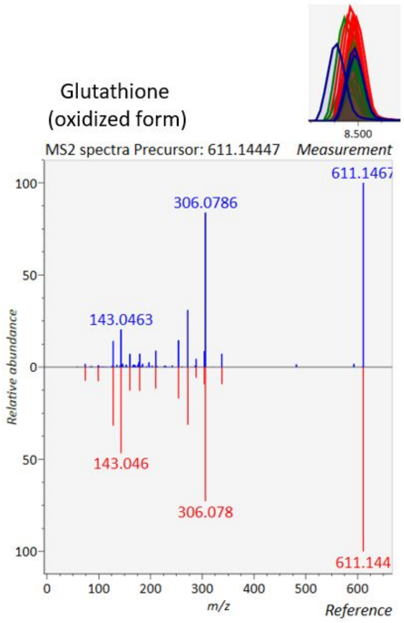

LPE 18:1

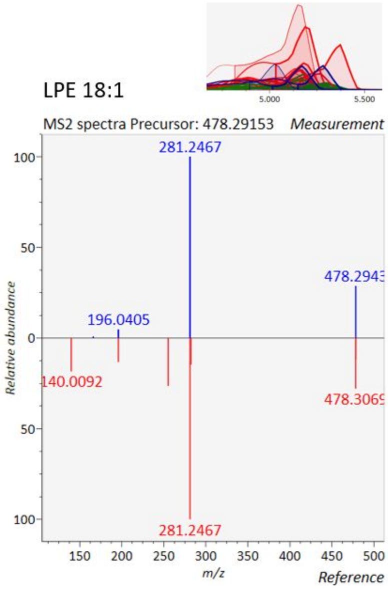

Guanine

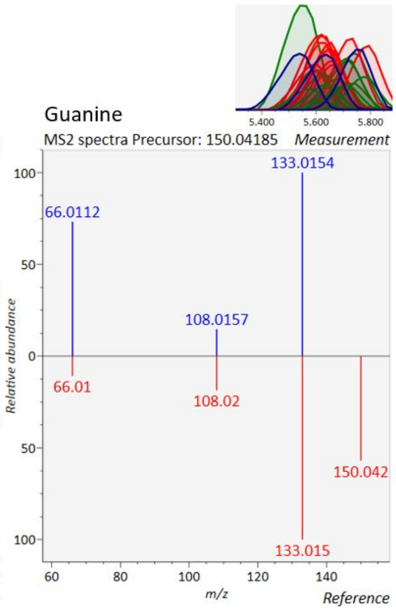

S-Adenosyl-L-homocysteine

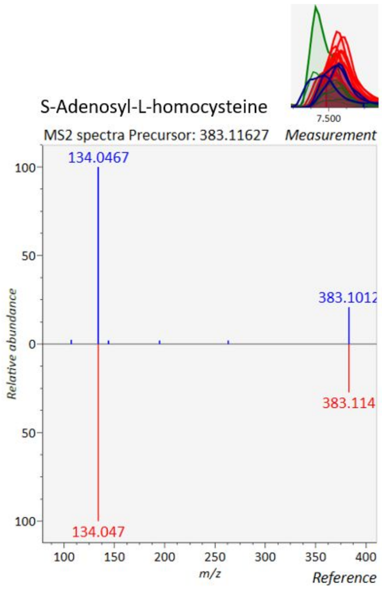

Adenosine-3-monophosphate

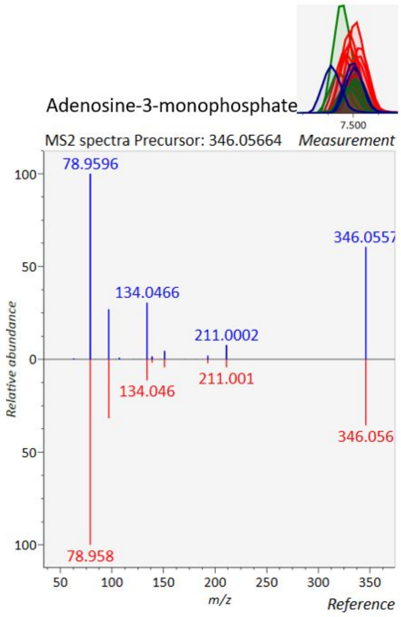

Uridine 5'-diphosphate

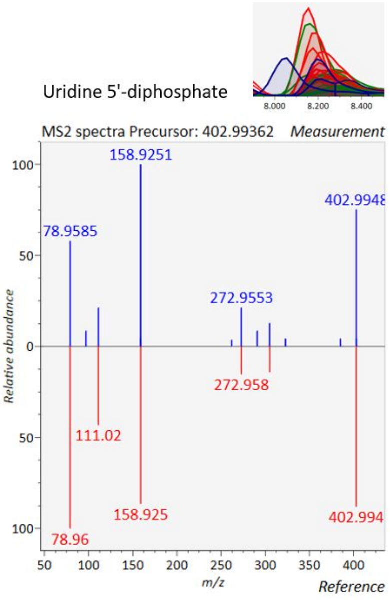

2-Ketogluconic acid

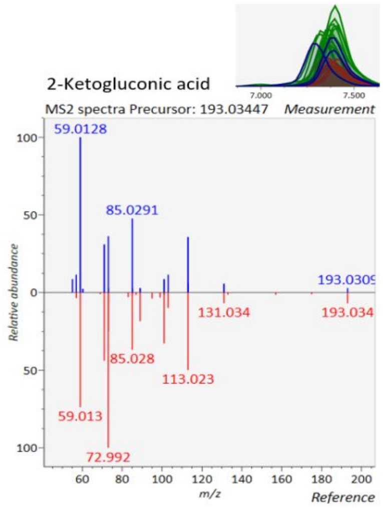

PFAP-diPAP; C22HF46O4P

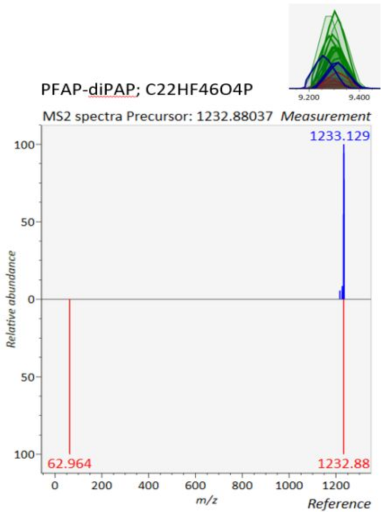

URIDINE

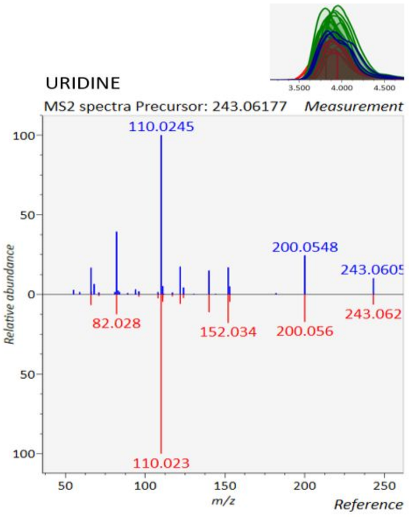

L-Glutamic acid

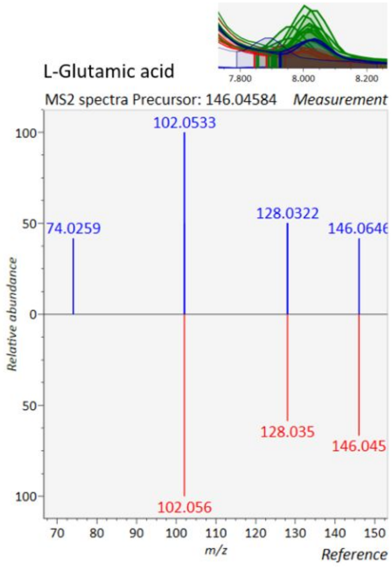

Nicotinic acid

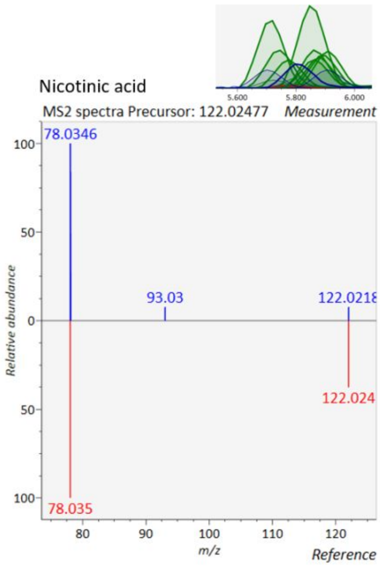

Pyridoxal

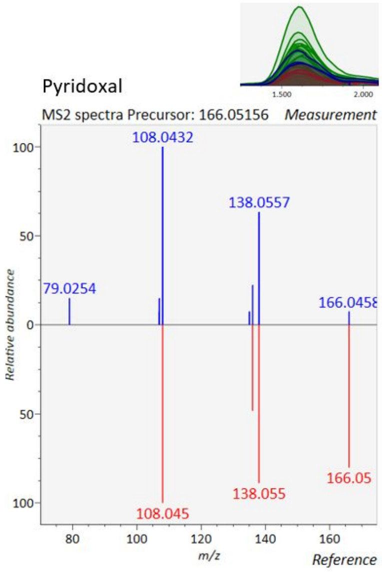

URIDINE-5-MONOPHOSPHATE

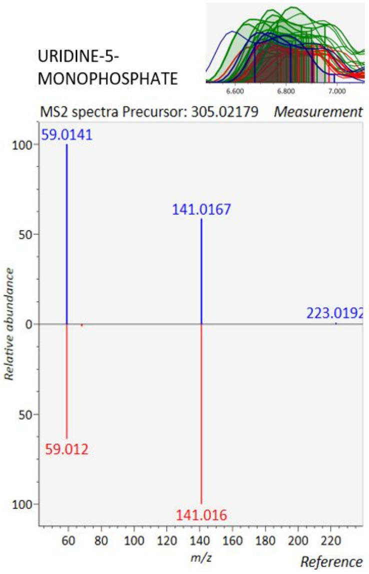

2,8-Quinolinediol

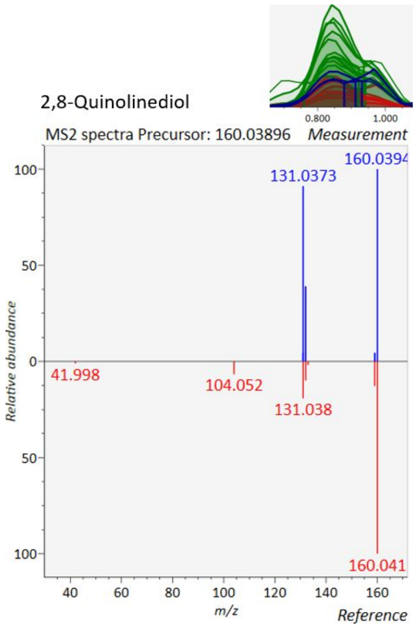

4-Hydroxyquinoline

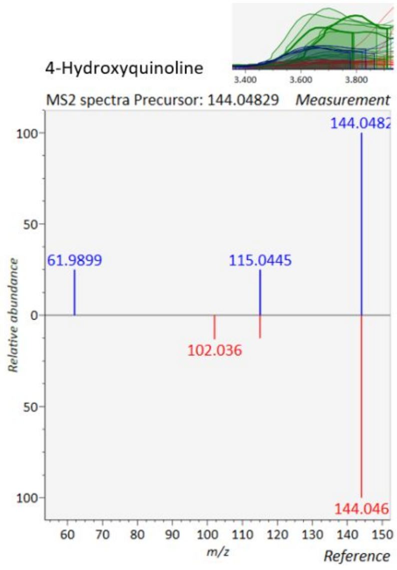

L-Pyroglutamic acid

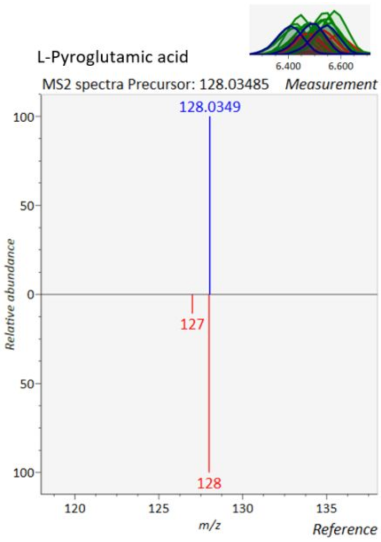

D-(+)-Raffinose

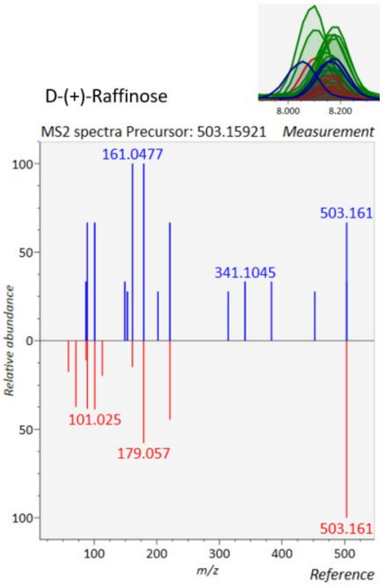

STACHYOSE HYDRATE

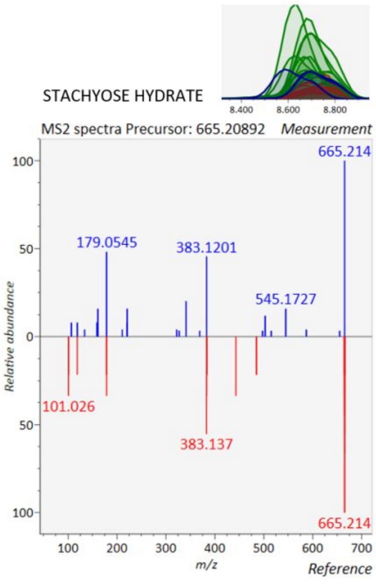

D-Glucose

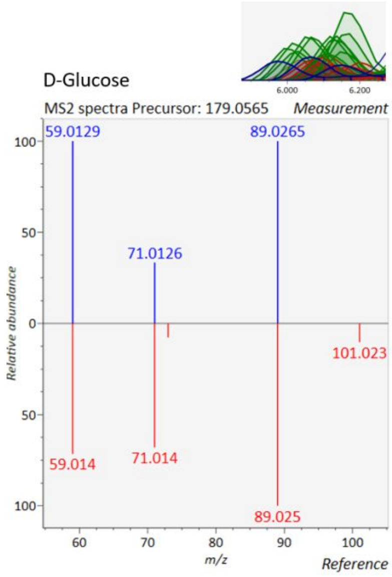

Glu-Gln

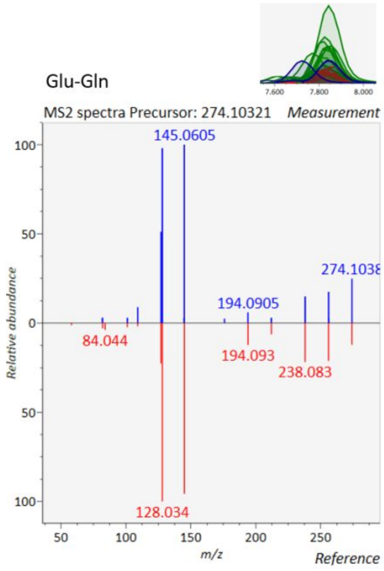

UDP-xylose

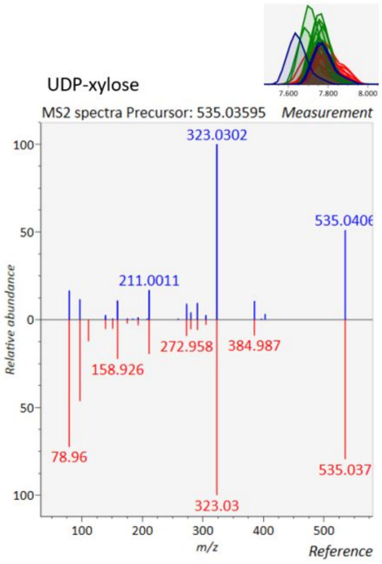

Guanosine cyclic monophosphate

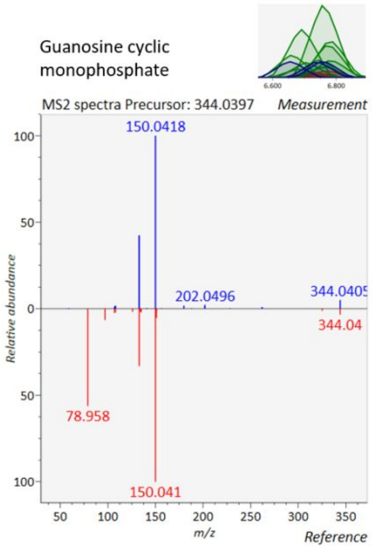

Guanosine cyclic monophosphate

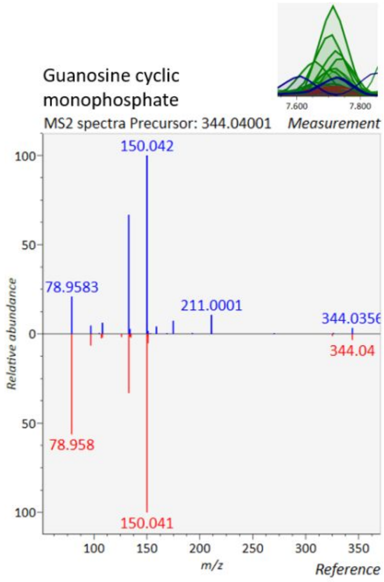

D-Glucosamine-6-phosphate

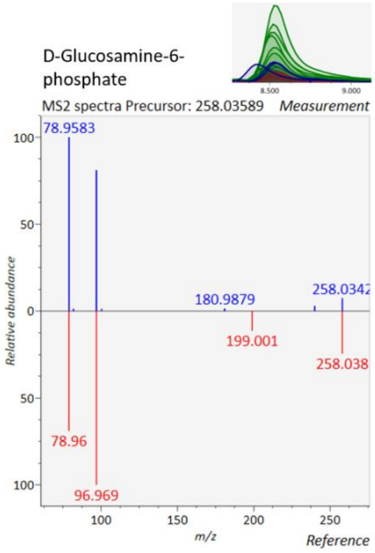

2-Hydroxyisobutyric acid

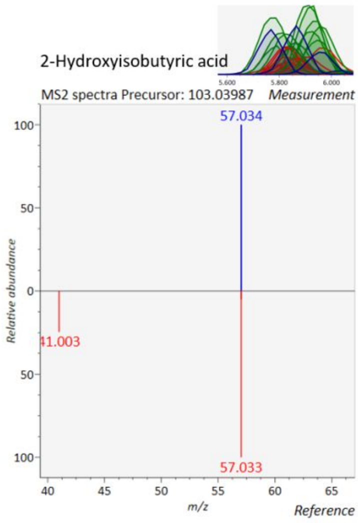

N-Acetylglutamic acid

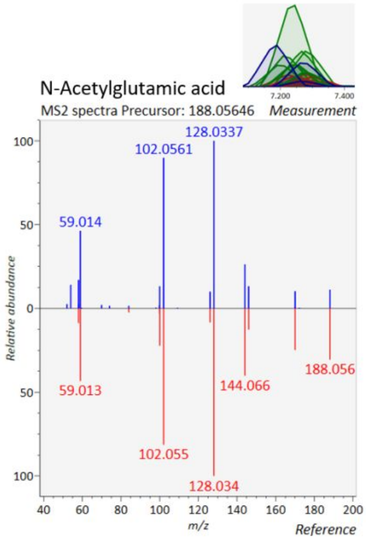

Sedoheptulose 7 -phosphate(S7P)

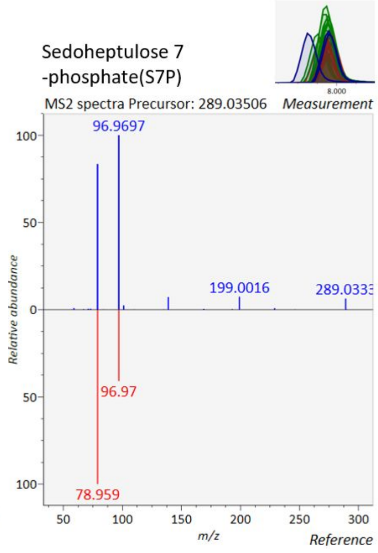

D-Glucosamine-6-phosphate

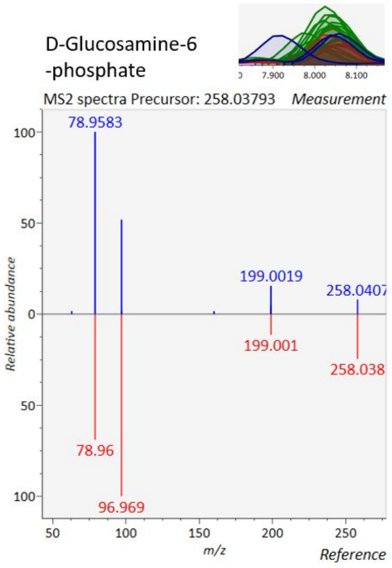

D-Mannose-6-phosphate

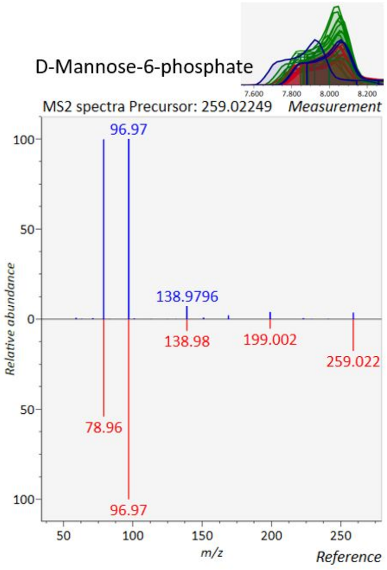

Cystathionine

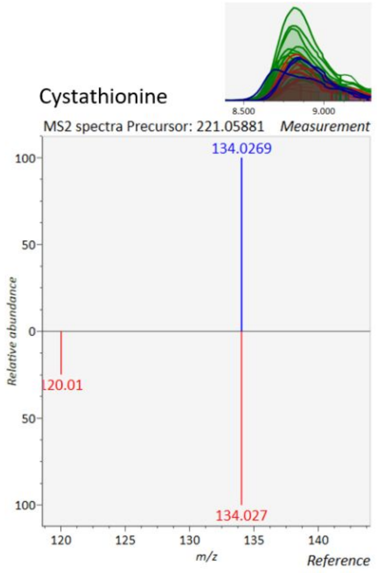

Adenine

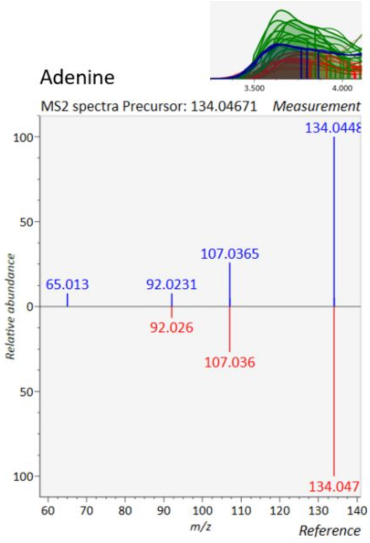

DEOXYCHOLIC ACID

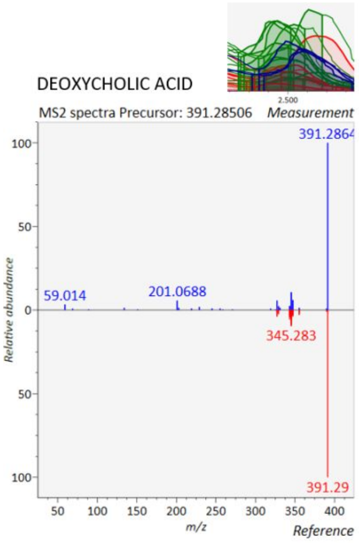

4-Hydroxyquinoline

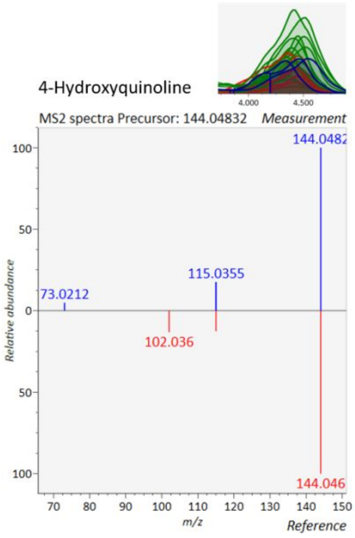

3-GluA Soyasapogenol B

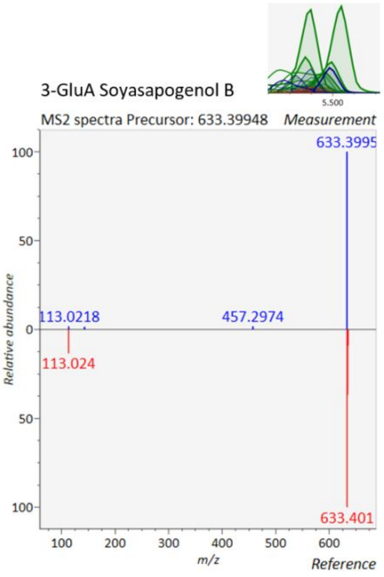

ACETOIN

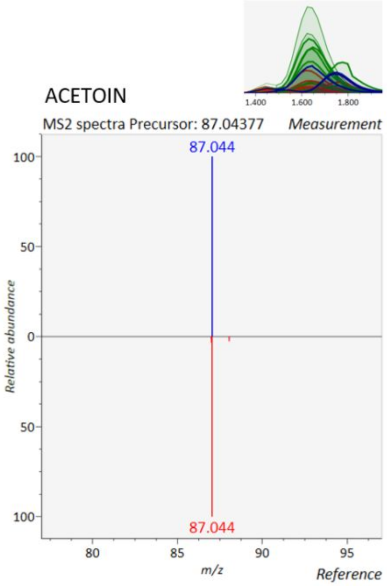

DEOXYCHOLIC ACID

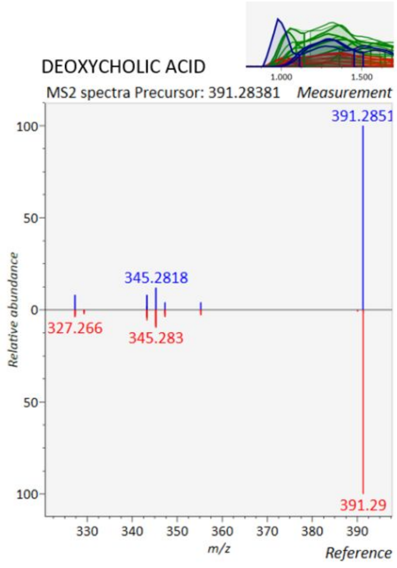

Succinic acid

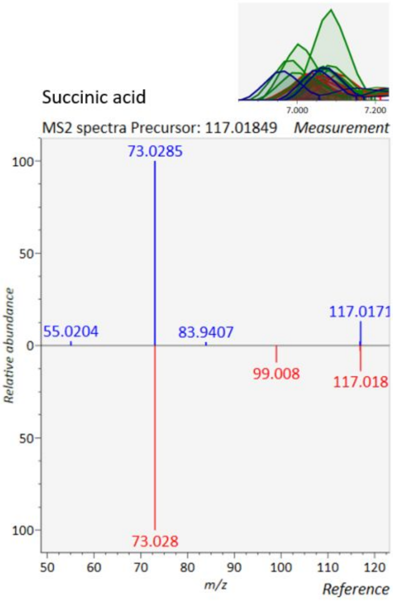

Appendix 2 MS/MS annotation for lipids by using MS DIAL.

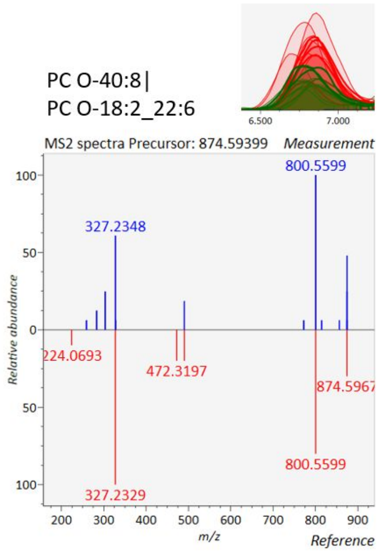

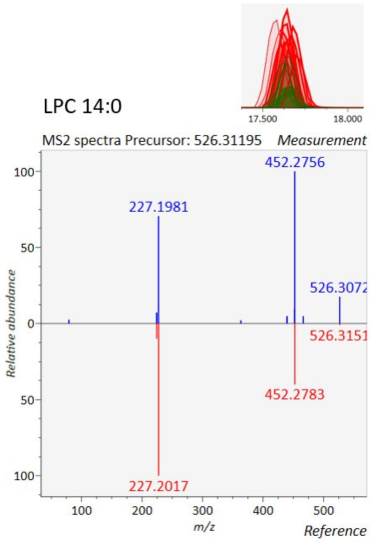

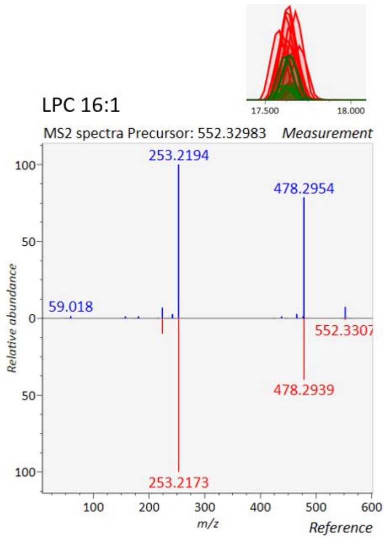

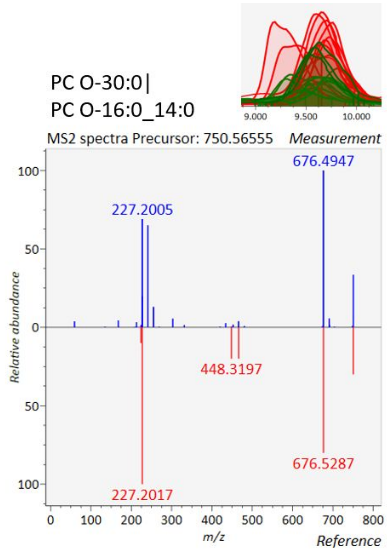

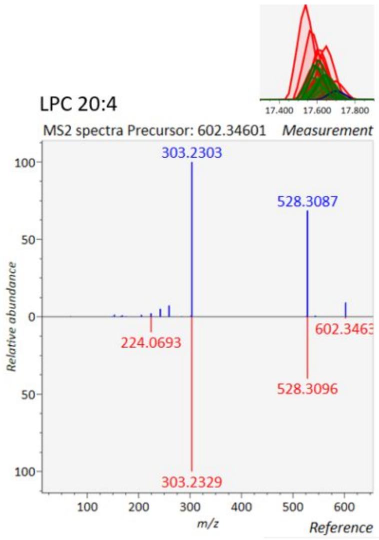

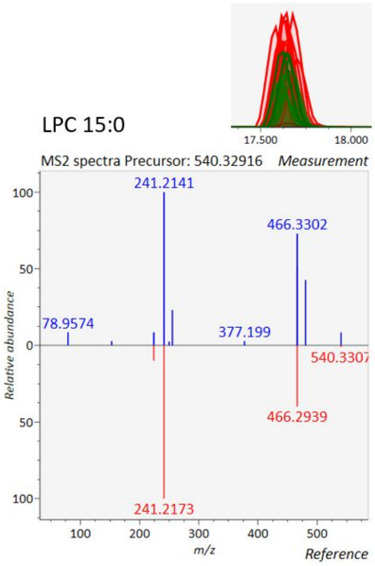

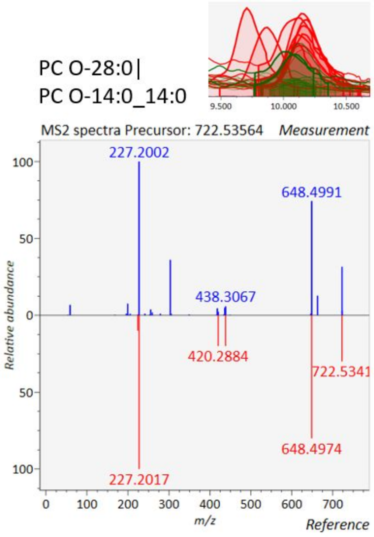

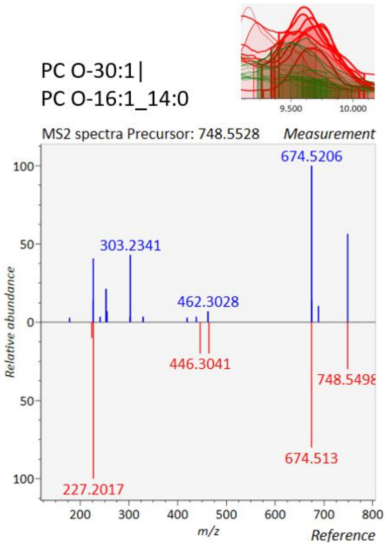

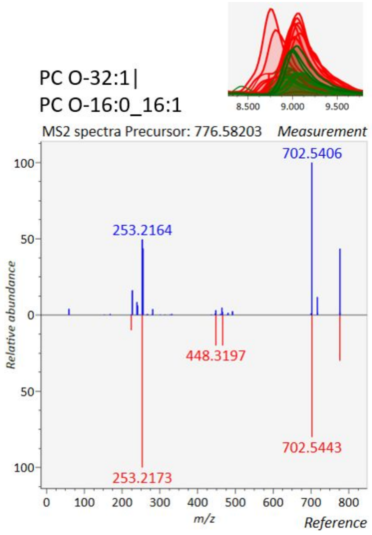

LPC 18:1

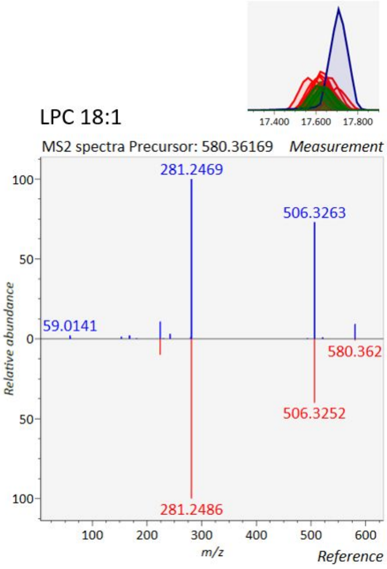

LPC 16:1

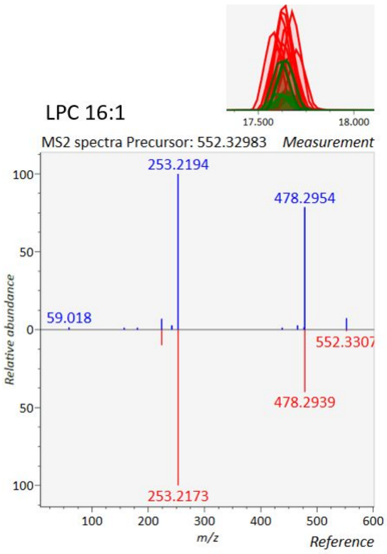

PC O-36:5|
PC O-16:1_20:4

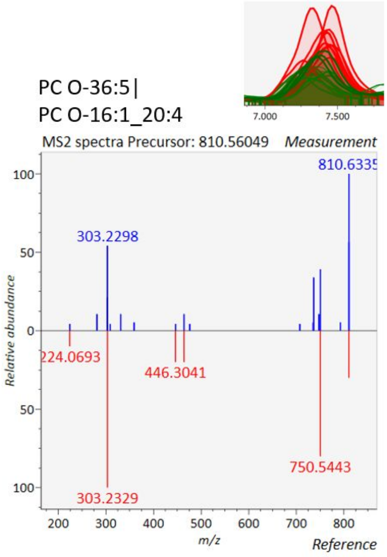

PE 32:1|
PE 16:0_16:1

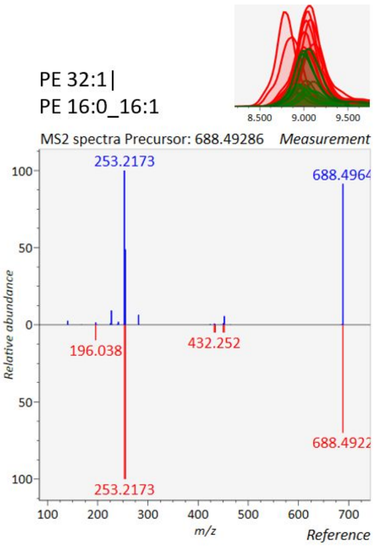

PE O-31:2|
PE O-15:1_16:1

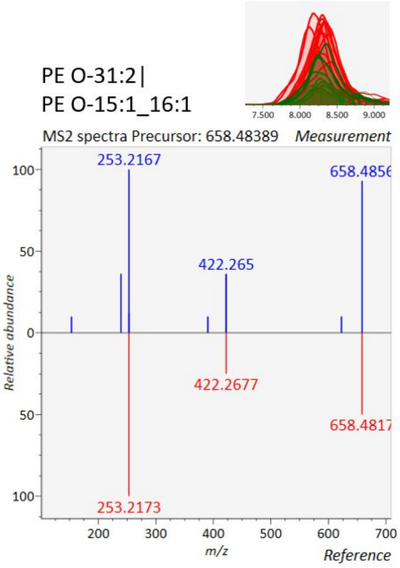

PE 36:5|
PE 16:1_20:4

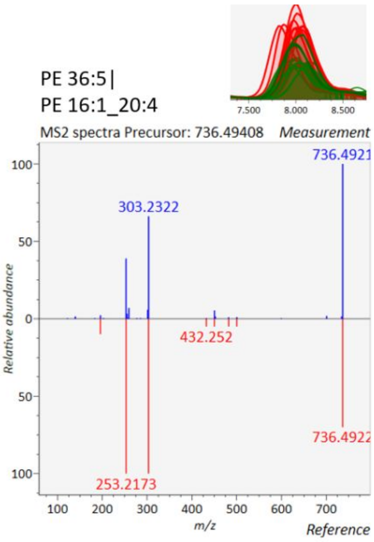

PE 34:1|
PE 16:0_18:1

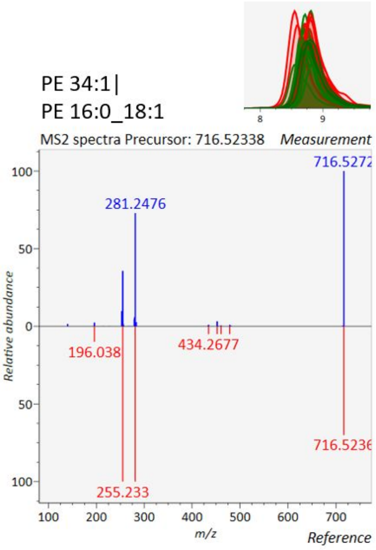

PE O-36:4|
PE O-16:1_20:3

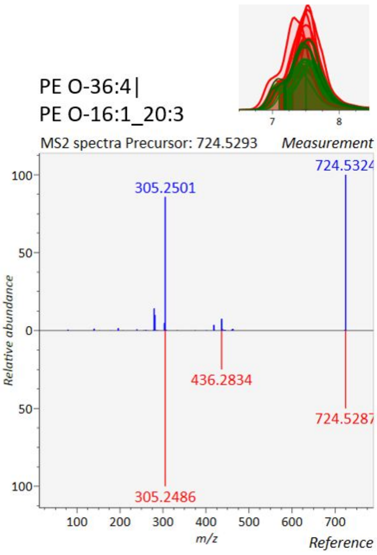

PE 32:0|
PE 16:0_16:0

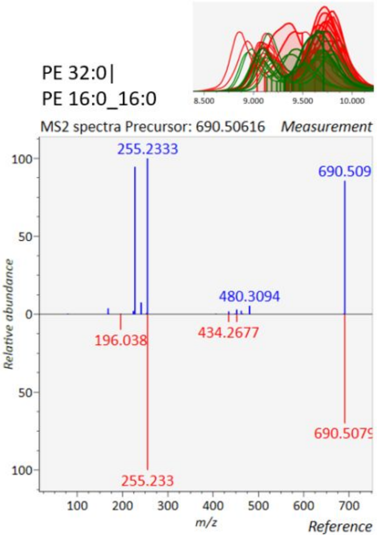

PE O-35:4|
PE O-15:1_20:3

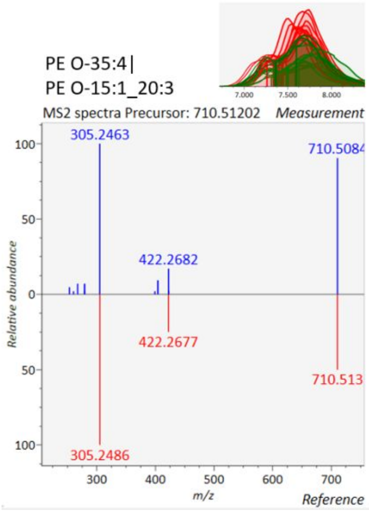

PE O-34:4|
PE O-16:1_18:3

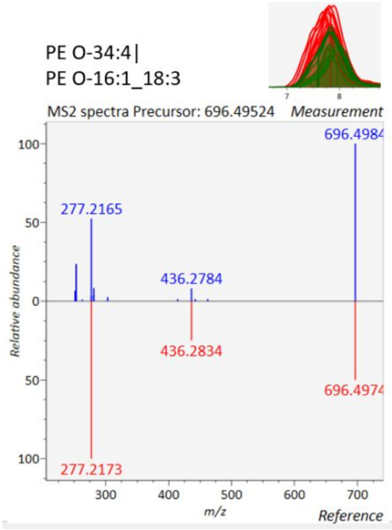

LPE 22:5

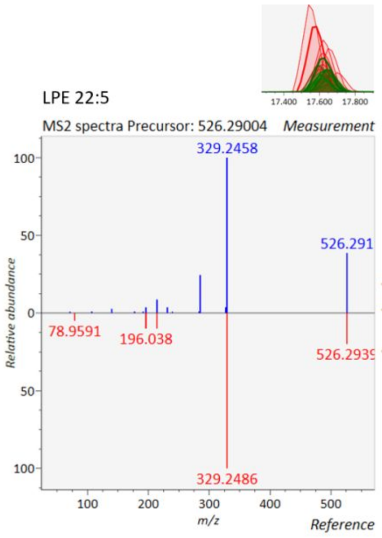

LPE 20:2

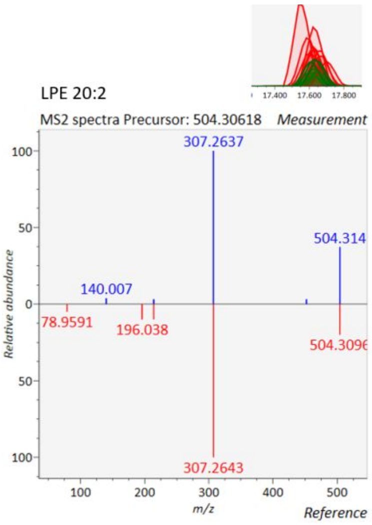

PE O-30:0|
PE O-16:0_14:0

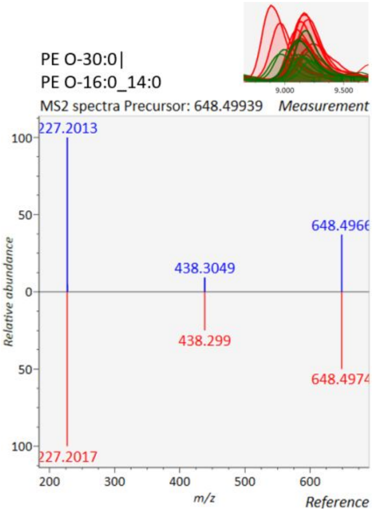

LPE 18:1

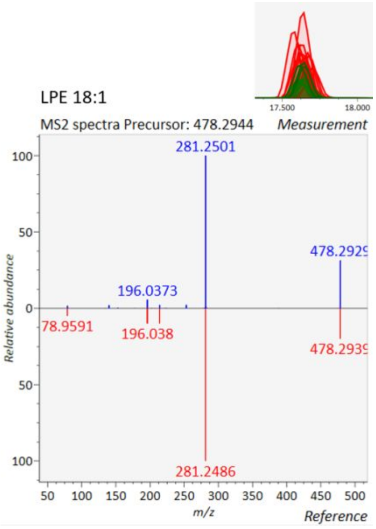

LPE 22:6

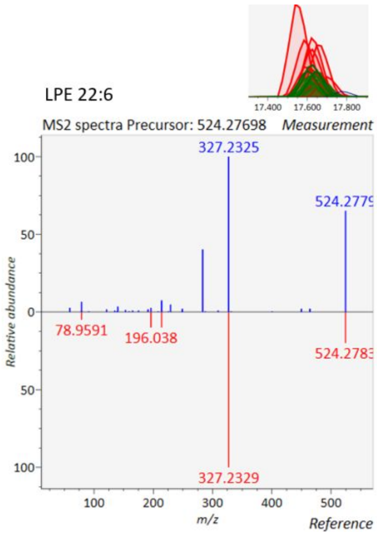

PE 32:2|
PE 16:1_16:1

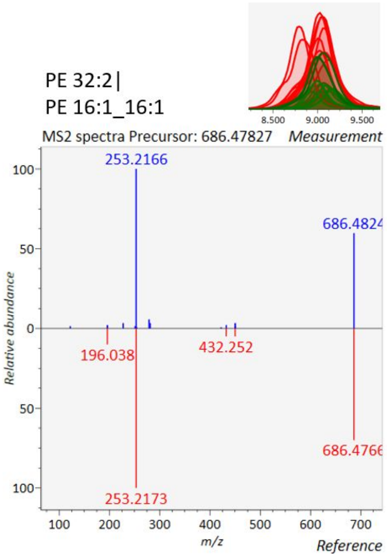

LPE 20:1

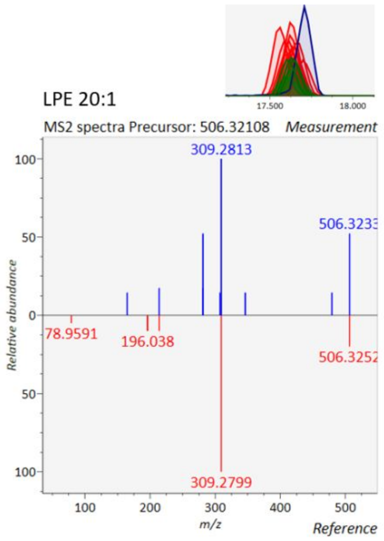

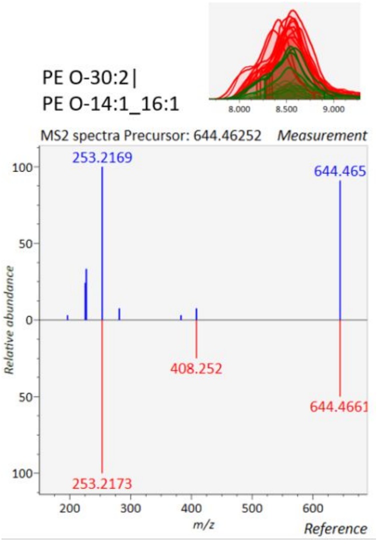
PE O-30:2|
PE O-14:1_16:1
MS2 spectra Precursor: 644.46252
Measurement
253.2169
644.465
408.252
644.4661
253.2173
Relative abundance
m/z
Reference

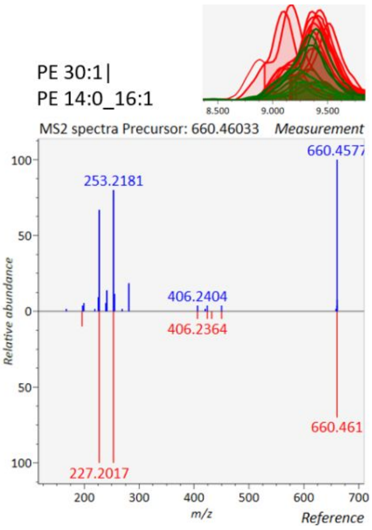
PE 30:1|
PE 14:0_16:1
MS2 spectra Precursor: 660.46033
Measurement
660.4577
253.2181
406.2404
406.2364
660.461
227.2017
Relative abundance
m/z
Reference

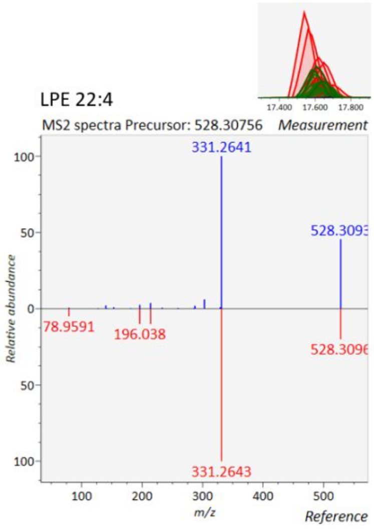
LPE 22:4
MS2 spectra Precursor: 528.30756
Measurement
331.2641
528.3093
78.9591
196.038
528.3096
331.2643
Relative abundance
m/z
Reference

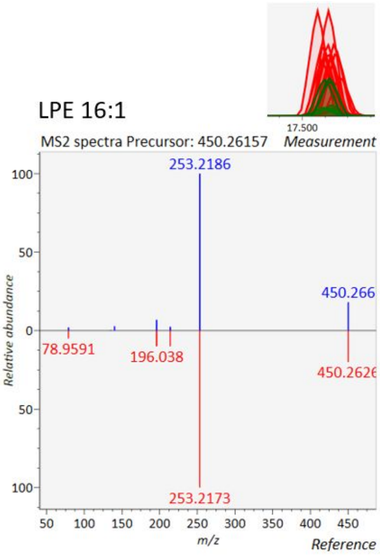
LPE 16:1
MS2 spectra Precursor: 450.26157
Measurement
253.2186
450.266
78.9591
196.038
450.2626
253.2173
Relative abundance
m/z
Reference

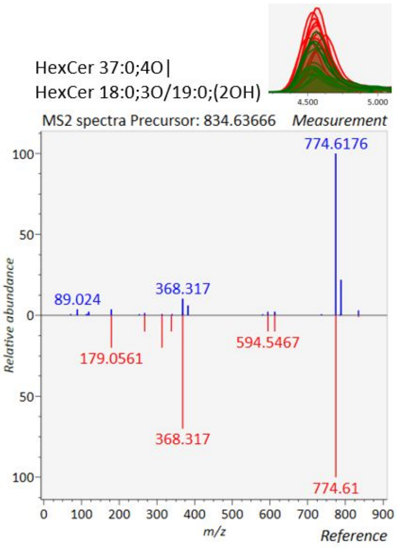
HexCer 37:0;4O|
HexCer 18:0;3O/19:0;(2OH)
MS2 spectra Precursor: 834.63666
Measurement
774.6176
89.024
368.317
179.0561
594.5467
368.317
774.61
Relative abundance
m/z
Reference

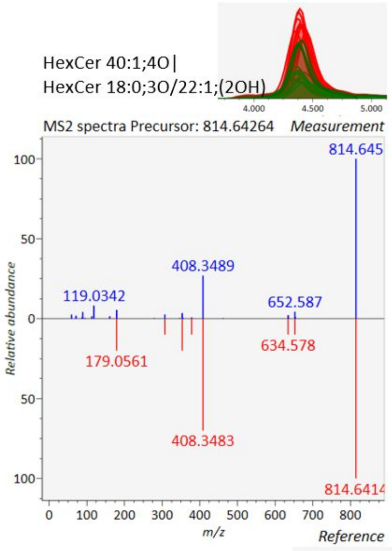
HexCer 40:1;4O|
HexCer 18:0;3O/22:1;(2OH)
MS2 spectra Precursor: 814.64264
Measurement
814.645
408.3489
119.0342
652.587
179.0561
634.578
408.3483
814.6414
Relative abundance
m/z
Reference

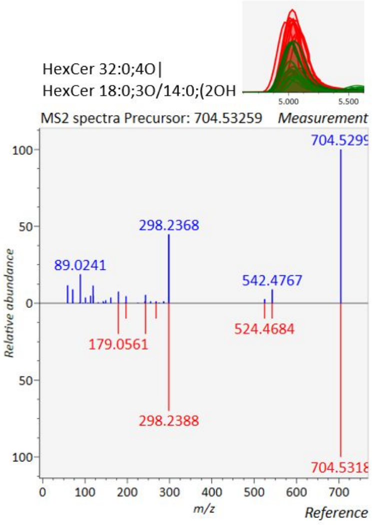
HexCer 32:0;4O|
HexCer 18:0;3O/14:0;(2OH
MS2 spectra Precursor: 704.53259
Measurement
704.5299
298.2368
89.0241
542.4767
179.0561
524.4684
298.2388
704.5318
Relative abundance
m/z
Reference

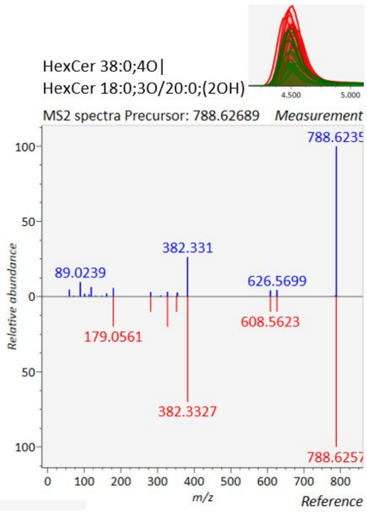
HexCer 38:0;4O|
HexCer 18:0;3O/20:0;(2OH)
MS2 spectra Precursor: 788.62689
Measurement
788.6235
382.331
89.0239
626.5699
179.0561
608.5623
382.3327
788.6257
Relative abundance
m/z
Reference

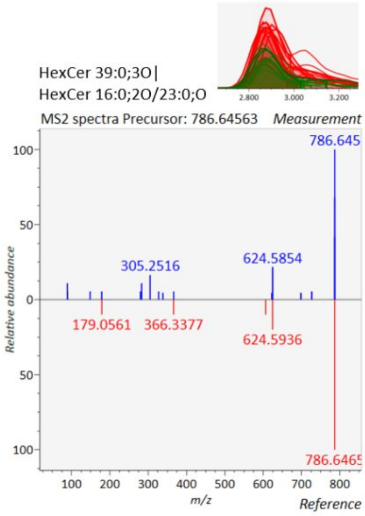
HexCer 39:0;3O|
HexCer 16:0;2O/23:0;O
MS2 spectra Precursor: 786.64563
Measurement
786.645
305.2516
624.5854
179.0561
366.3377
624.5936
786.6465
Relative abundance
m/z
Reference

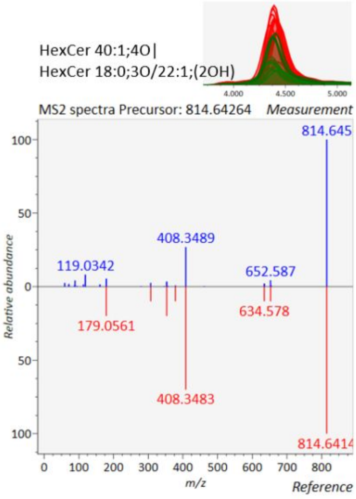
HexCer 40:1;4O|
HexCer 18:0;3O/22:1;(2OH)
MS2 spectra Precursor: 814.64264
Measurement
814.645
408.3489
119.0342
652.587
179.0561
634.578
408.3483
814.6414
Relative abundance
m/z
Reference

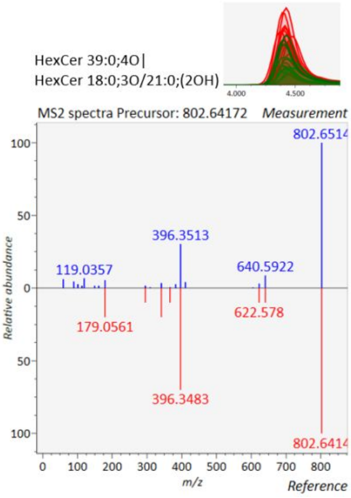
HexCer 39:0;4O|
HexCer 18:0;3O/21:0;(2OH)
MS2 spectra Precursor: 802.64172
Measurement
802.6514
396.3513
119.0357
640.5922
179.0561
622.578
396.3483
802.6414
Relative abundance
m/z
Reference

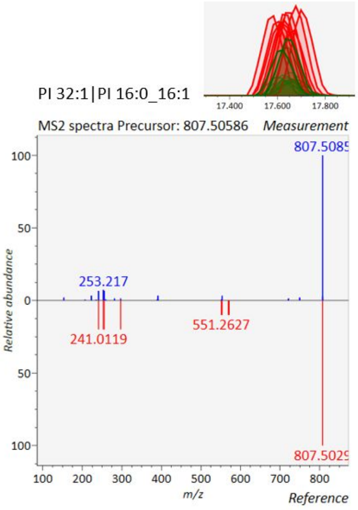
PI 32:1|PI 16:0_16:1
MS2 spectra Precursor: 807.50586
Measurement
807.5085
253.217
241.0119
551.2627
807.5029
Relative abundance
m/z
Reference

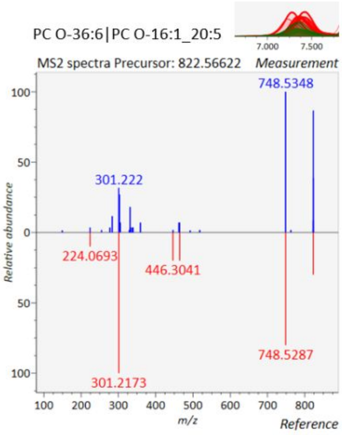
PC O-36:6|PC O-16:1_20:5
MS2 spectra Precursor: 822.56622
Measurement
748.5348
301.222
224.0693
446.3041
748.5287
301.2173
Relative abundance
m/z
Reference

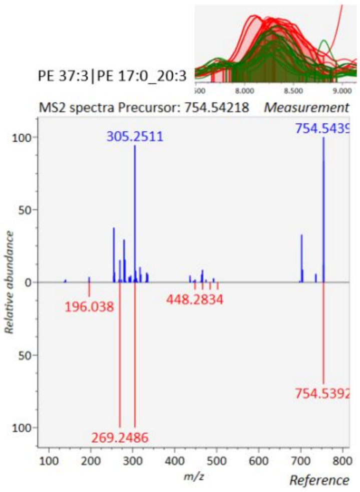
PE 37:3|PE 17:0_20:3
MS2 spectra Precursor: 754.54218
Measurement
305.2511
754.5439
196.038
448.2834
754.5392
269.2486
Relative abundance
m/z
Reference

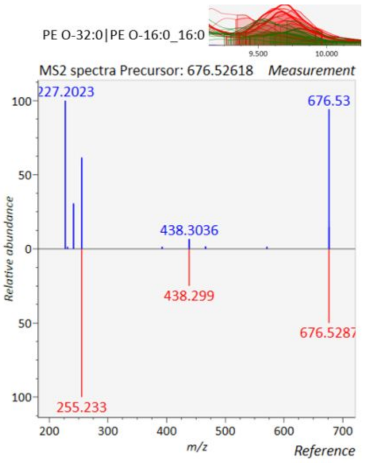
PE O-32:0|PE O-16:0_16:0
MS2 spectra Precursor: 676.52618
Measurement
227.2023
676.53
438.3036
438.299
676.5287
255.233
Relative abundance
m/z
Reference

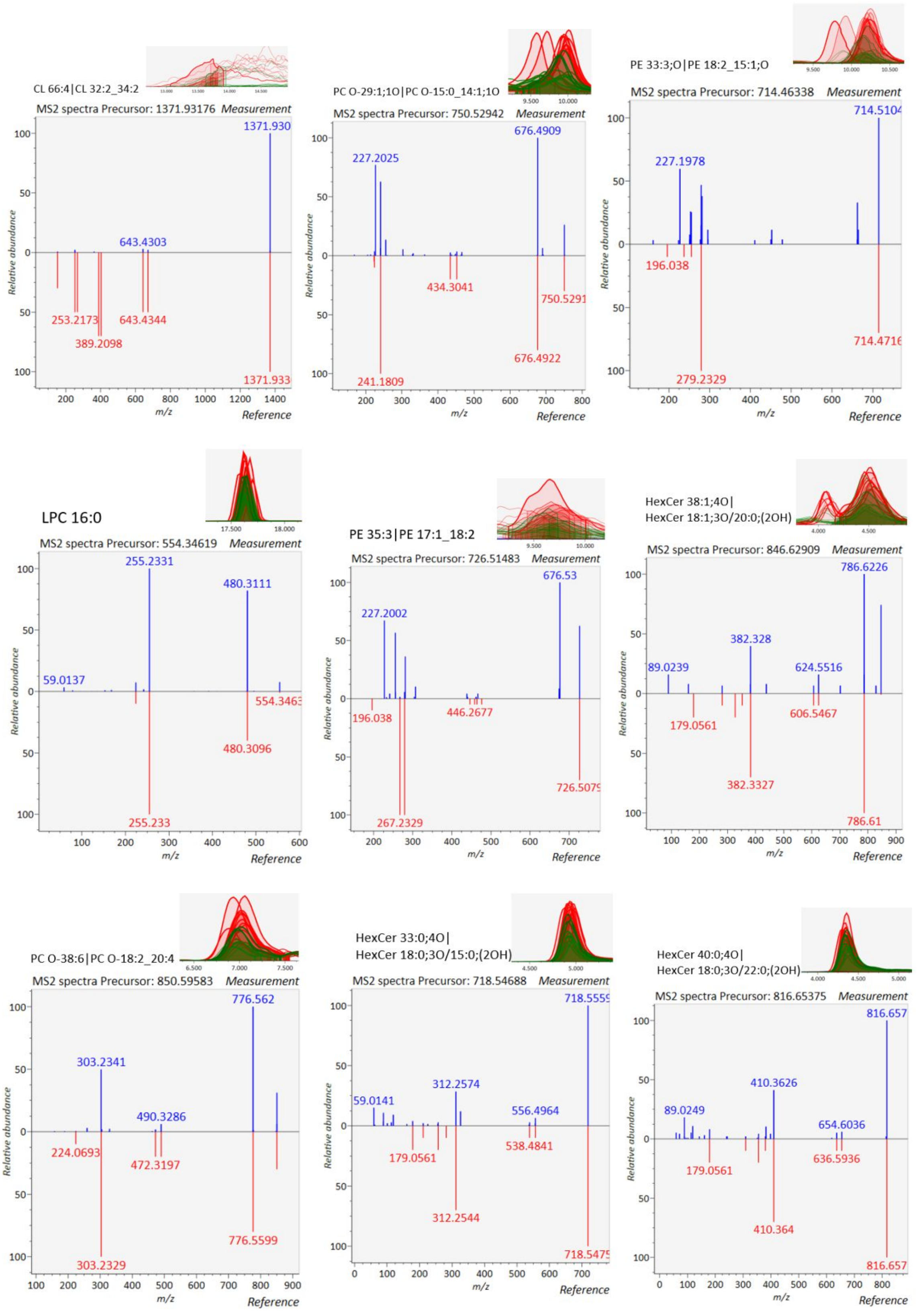
CL 66:4|CL 32:2_34:2
MS2 spectra Precursor: 1371.93176 Measurement
1371.930
643.4303
253.2173
389.2098
643.4344
1371.933
Relative abundance
m/z
Reference
PC O-29:1;1O|PC O-15:0_14:1;1O
MS2 spectra Precursor: 750.52942 Measurement
227.2025
676.4909
434.3041
750.5291
676.4922
241.1809
PE 33:3;O|PE 18:2_15:1;O
MS2 spectra Precursor: 714.46338 Measurement
714.5104
227.1978
196.038
714.4716
279.2329
LPC 16:0
MS2 spectra Precursor: 554.34619 Measurement
255.2331
480.3111
59.0137
554.3463
480.3096
255.233
PE 35:3|PE 17:1_18:2
MS2 spectra Precursor: 726.51483 Measurement
676.53
227.2002
196.038
446.2677
726.5079
267.2329
HexCer 38:1;4O|
HexCer 18:1;3O/20:0;(2OH)
MS2 spectra Precursor: 846.62909 Measurement
786.6226
382.328
89.0239
624.5516
179.0561
606.5467
382.3327
786.61
PC O-38:6|PC O-18:2_20:4
MS2 spectra Precursor: 850.59583 Measurement
776.562
303.2341
490.3286
224.0693
472.3197
776.5599
303.2329
HexCer 33:0;4O|
HexCer 18:0;3O/15:0;(2OH)
MS2 spectra Precursor: 718.54688 Measurement
718.5559
312.2574
59.0141
556.4964
179.0561
538.4841
312.2544
718.5475
HexCer 40:0;4O|
HexCer 18:0;3O/22:0;(2OH)
MS2 spectra Precursor: 816.65375 Measurement
816.657
410.3626
89.0249
654.6036
179.0561
636.5936
410.364
816.657

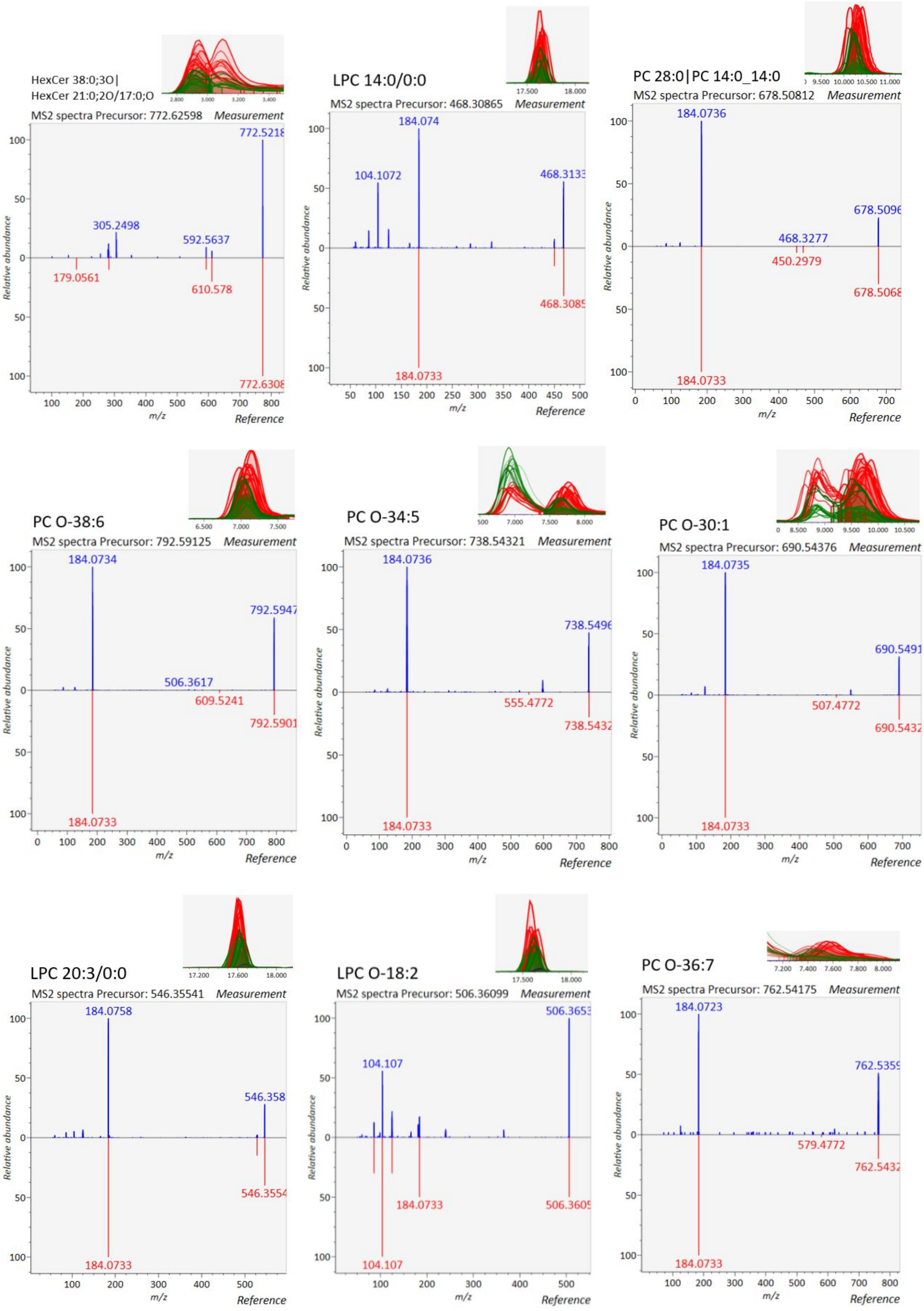
HexCer 38:0;3O|
HexCer 21:0;2O/17:0;O
MS2 spectra Precursor: 772.62598 Measurement
772.5218
305.2498
592.5637
179.0561
610.578
772.6308
Relative abundance
m/z
Reference
LPC 14:0/0:0
MS2 spectra Precursor: 468.30865 Measurement
184.074
104.1072
468.3133
468.3085
184.0733
PC 28:0|PC 14:0_14:0
MS2 spectra Precursor: 678.50812 Measurement
184.0736
678.5096
468.3277
450.2979
678.5068
184.0733
PC O-38:6
MS2 spectra Precursor: 792.59125 Measurement
184.0734
792.5947
506.3617
609.5241
792.5901
184.0733
PC O-34:5
MS2 spectra Precursor: 738.54321 Measurement
184.0736
738.5496
555.4772
738.5432
184.0733
PC O-30:1
MS2 spectra Precursor: 690.54376 Measurement
184.0735
690.5491
507.4772
690.5432
184.0733
LPC 20:3/0:0
MS2 spectra Precursor: 546.35541 Measurement
184.0758
546.358
546.3554
184.0733
LPC O-18:2
MS2 spectra Precursor: 506.36099 Measurement
506.3653
104.107
184.0733
506.3605
104.107
PC O-36:7
MS2 spectra Precursor: 762.54175 Measurement
184.0723
762.5359
579.4772
762.5432
184.0733

PC O-30:0

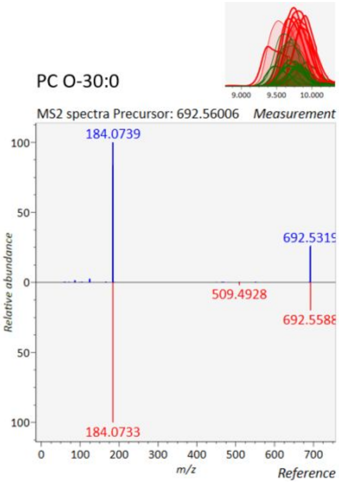

LPC 28:0

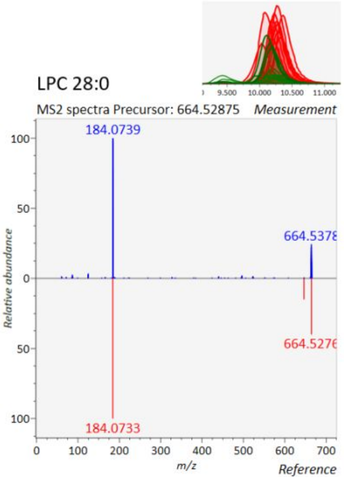

PC 32:3|PC 16:0_16:3

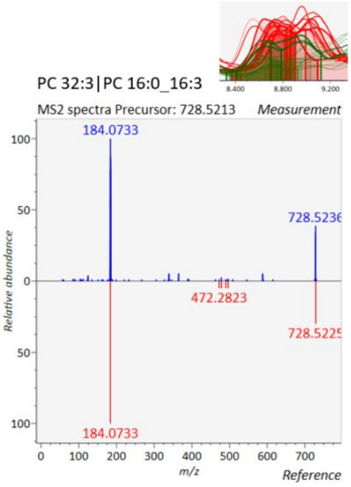

LPC 16:1/0:0

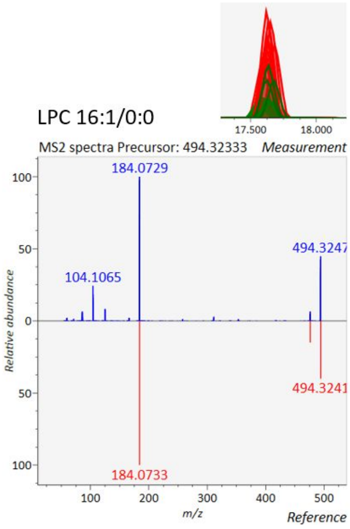

PC 30:1|PC 14:0_16:1

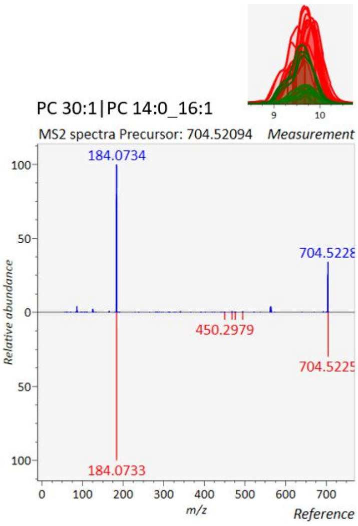

PC 29:0|PC 14:0_15:0

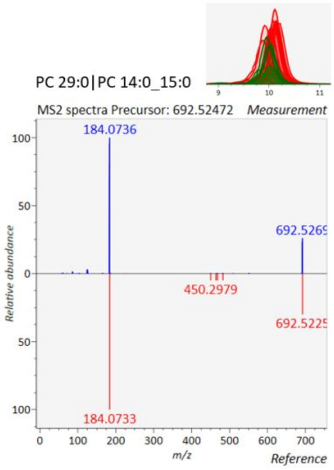

PC O-36:6

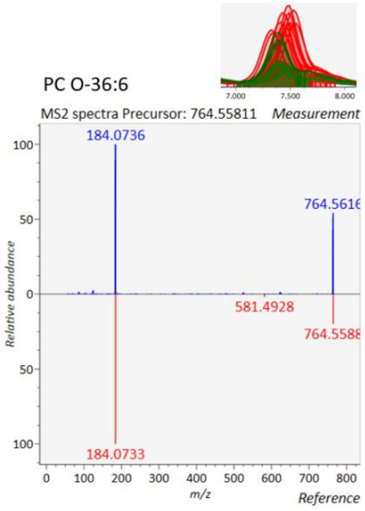

LPC 20:1/0:0

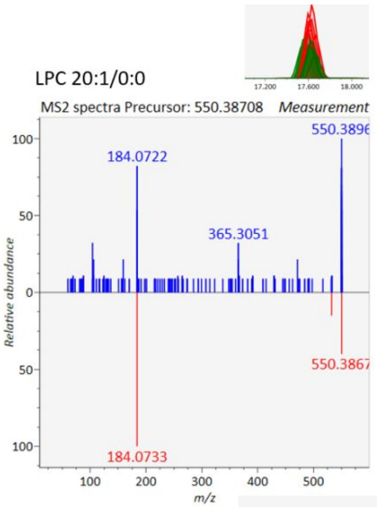

LPE O-20:4

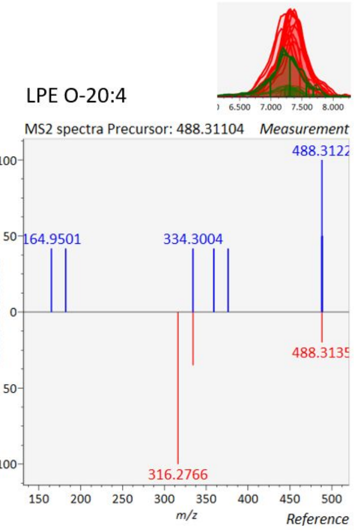

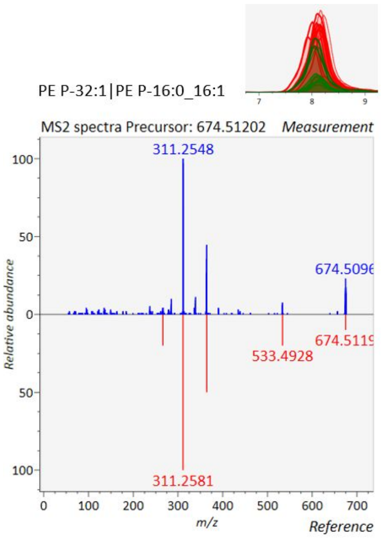
PE P-32:1|PE P-16:0_16:1
MS2 spectra Precursor: 674.51202
Measurement
311.2548
674.5096
674.5119
533.4928
311.2581
Relative abundance
m/z
Reference

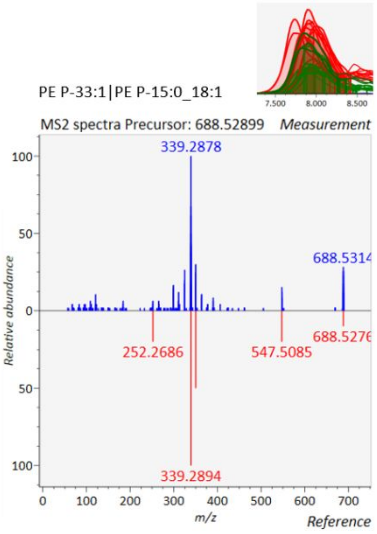
PE P-33:1|PE P-15:0_18:1
MS2 spectra Precursor: 688.52899
Measurement
339.2878
688.5314
688.5276
252.2686
547.5085
339.2894
Relative abundance
m/z
Reference

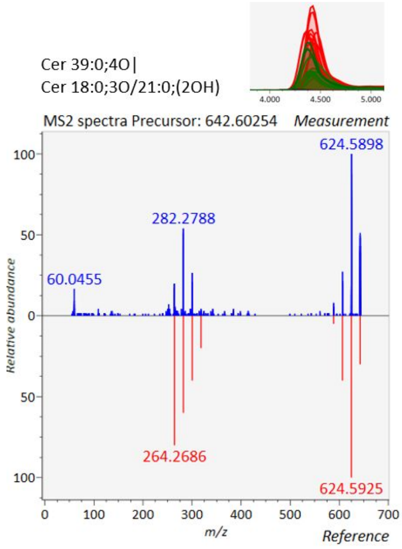
Cer 39:0;4O|
Cer 18:0;3O/21:0;(2OH)
MS2 spectra Precursor: 642.60254
Measurement
624.5898
282.2788
60.0455
264.2686
624.5925
Relative abundance
m/z
Reference

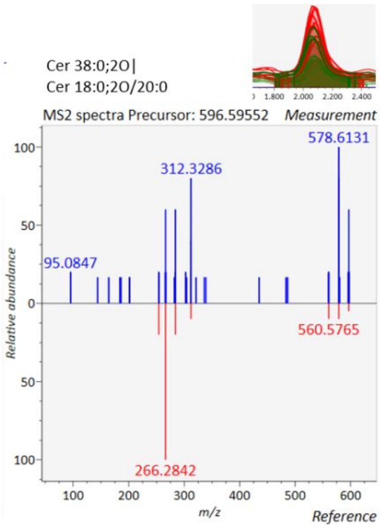
Cer 38:0;2O|
Cer 18:0;2O/20:0
MS2 spectra Precursor: 596.59552
Measurement
578.6131
312.3286
95.0847
560.5765
266.2842
Relative abundance
m/z
Reference

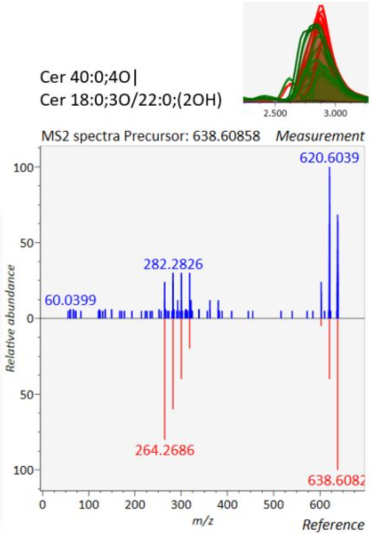
Cer 40:0;4O|
Cer 18:0;3O/22:0;(2OH)
MS2 spectra Precursor: 638.60858
Measurement
620.6039
282.2826
60.0399
264.2686
638.6082
Relative abundance
m/z
Reference

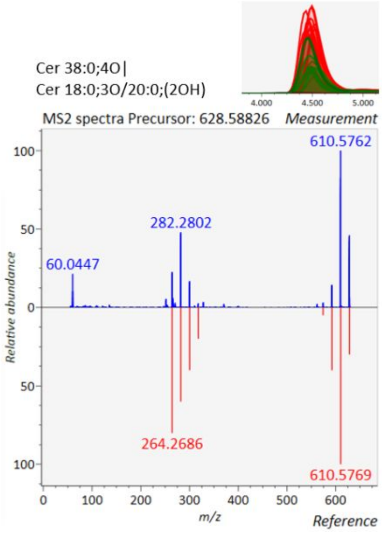
Cer 38:0;4O|
Cer 18:0;3O/20:0;(2OH)
MS2 spectra Precursor: 628.58826
Measurement
610.5762
282.2802
60.0447
264.2686
610.5769
Relative abundance
m/z
Reference

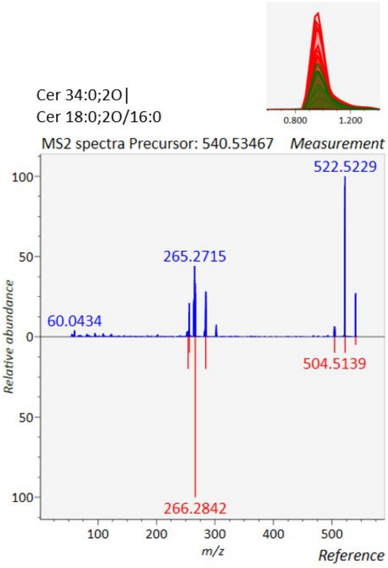
Cer 34:0;2O|
Cer 18:0;2O/16:0
MS2 spectra Precursor: 540.53467
Measurement
522.5229
265.2715
60.0434
504.5139
266.2842
Relative abundance
m/z
Reference

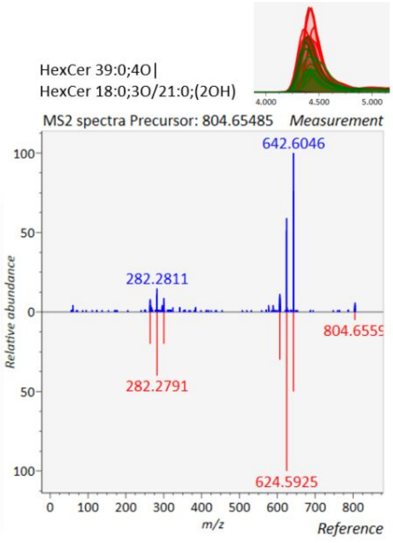
HexCer 39:0;4O|
HexCer 18:0;3O/21:0;(2OH)
MS2 spectra Precursor: 804.65485
Measurement
642.6046
282.2811
804.6559
282.2791
624.5925
Relative abundance
m/z
Reference

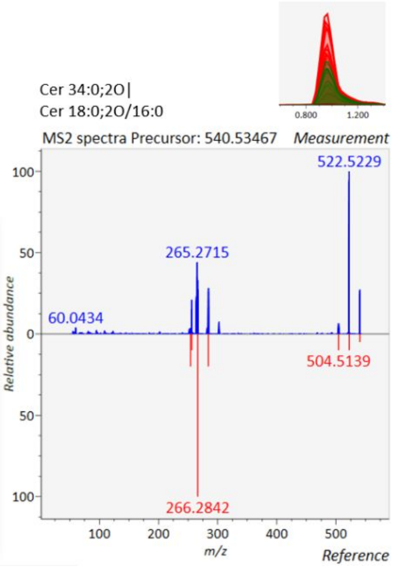
Cer 34:0;2O|
Cer 18:0;2O/16:0
MS2 spectra Precursor: 540.53467
Measurement
522.5229
265.2715
60.0434
504.5139
266.2842
Relative abundance
m/z
Reference

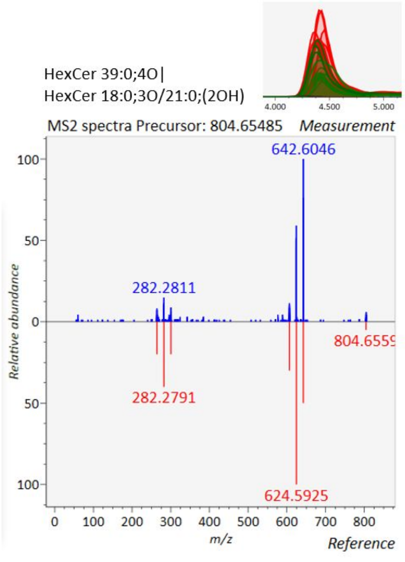
HexCer 39:0;4O|
HexCer 18:0;3O/21:0;(2OH)
4.000
4.500
5.000
MS2 spectra Precursor: 804.65485
Measurement
642.6046
282.2811
804.6559
282.2791
624.5925
Relative abundance
m/z
Reference

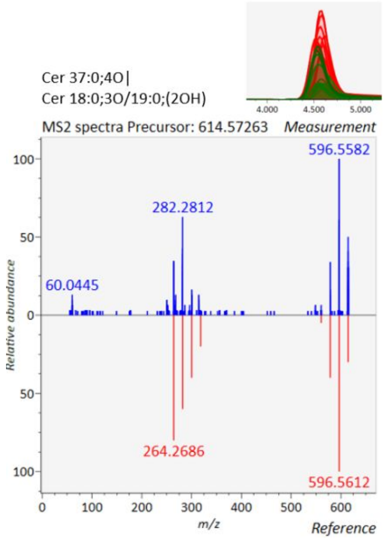
Cer 37:0;4O|
Cer 18:0;3O/19:0;(2OH)
4.000
4.500
5.000
MS2 spectra Precursor: 614.57263
Measurement
596.5582
282.2812
60.0445
264.2686
596.5612
Relative abundance
m/z
Reference

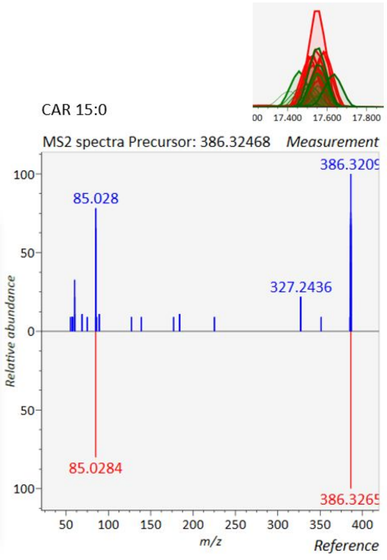
CAR 15:0
17.400
17.600
17.800
MS2 spectra Precursor: 386.32468
Measurement
386.3209
85.028
327.2436
85.0284
386.3265
Relative abundance
m/z
Reference

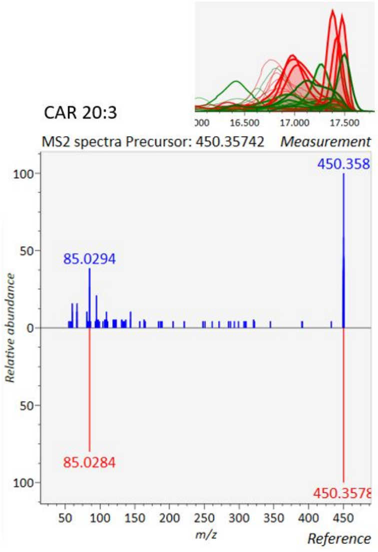
CAR 20:3
16.500
17.000
17.500
MS2 spectra Precursor: 450.35742
Measurement
450.358
85.0294
85.0284
450.3578
Relative abundance
m/z
Reference

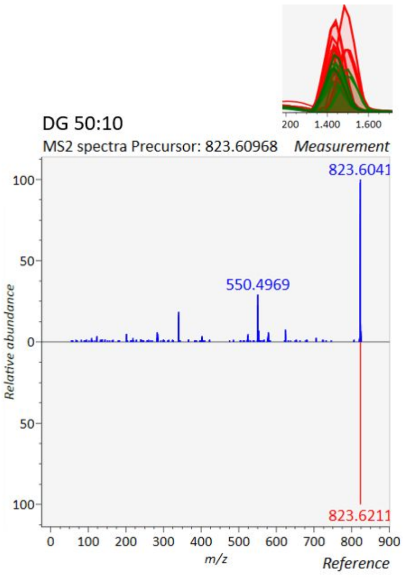
DG 50:10
1.400
1.600
MS2 spectra Precursor: 823.60968
Measurement
823.6041
550.4969
823.6211
Relative abundance
m/z
Reference

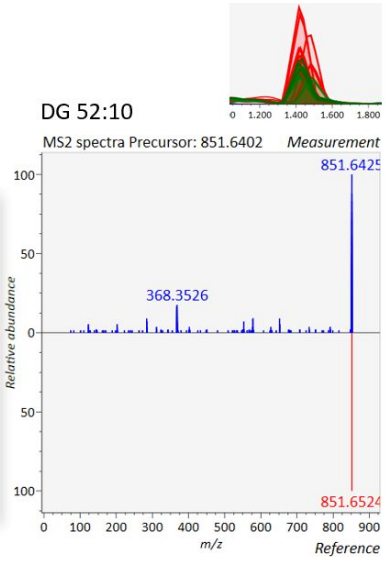
DG 52:10
1.200
1.400
1.600
1.800
MS2 spectra Precursor: 851.6402
Measurement
851.6425
368.3526
851.6524
Relative abundance
m/z
Reference

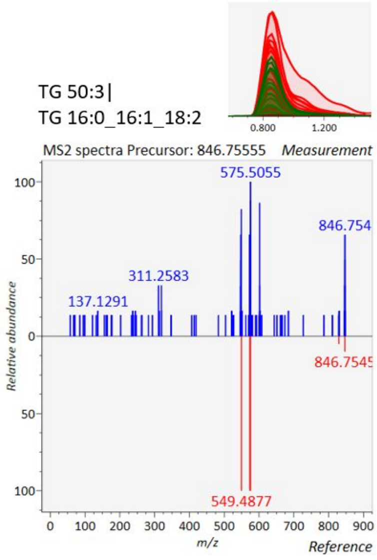
TG 50:3|
TG 16:0_16:1_18:2
0.800
1.200
MS2 spectra Precursor: 846.75555
Measurement
575.5055
846.754
311.2583
137.1291
846.7545
549.4877
Relative abundance
m/z
Reference

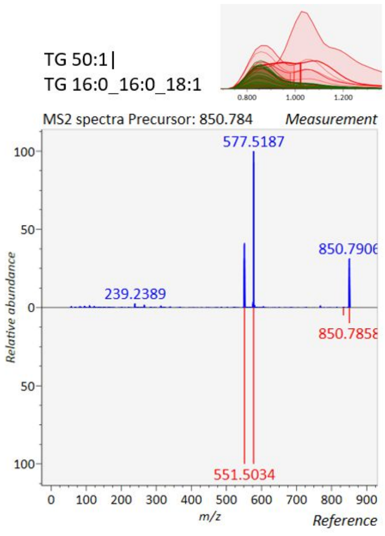
TG 50:1|
TG 16:0_16:0_18:1
0.800
1.000
1.200
MS2 spectra Precursor: 850.784
Measurement
577.5187
850.7906
239.2389
850.7858
551.5034
Relative abundance
m/z
Reference

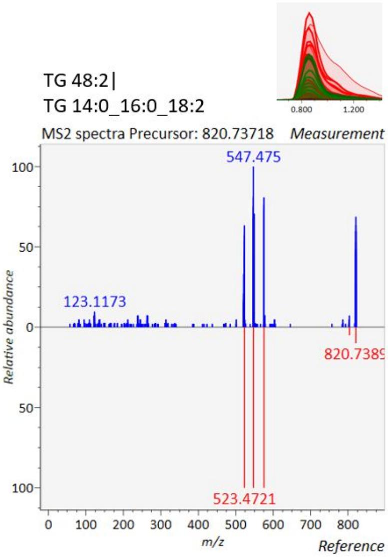
TG 48:2|
TG 14:0_16:0_18:2
0.800
1.200
MS2 spectra Precursor: 820.73718
Measurement
547.475
123.1173
820.7389
523.4721
Relative abundance
m/z
Reference

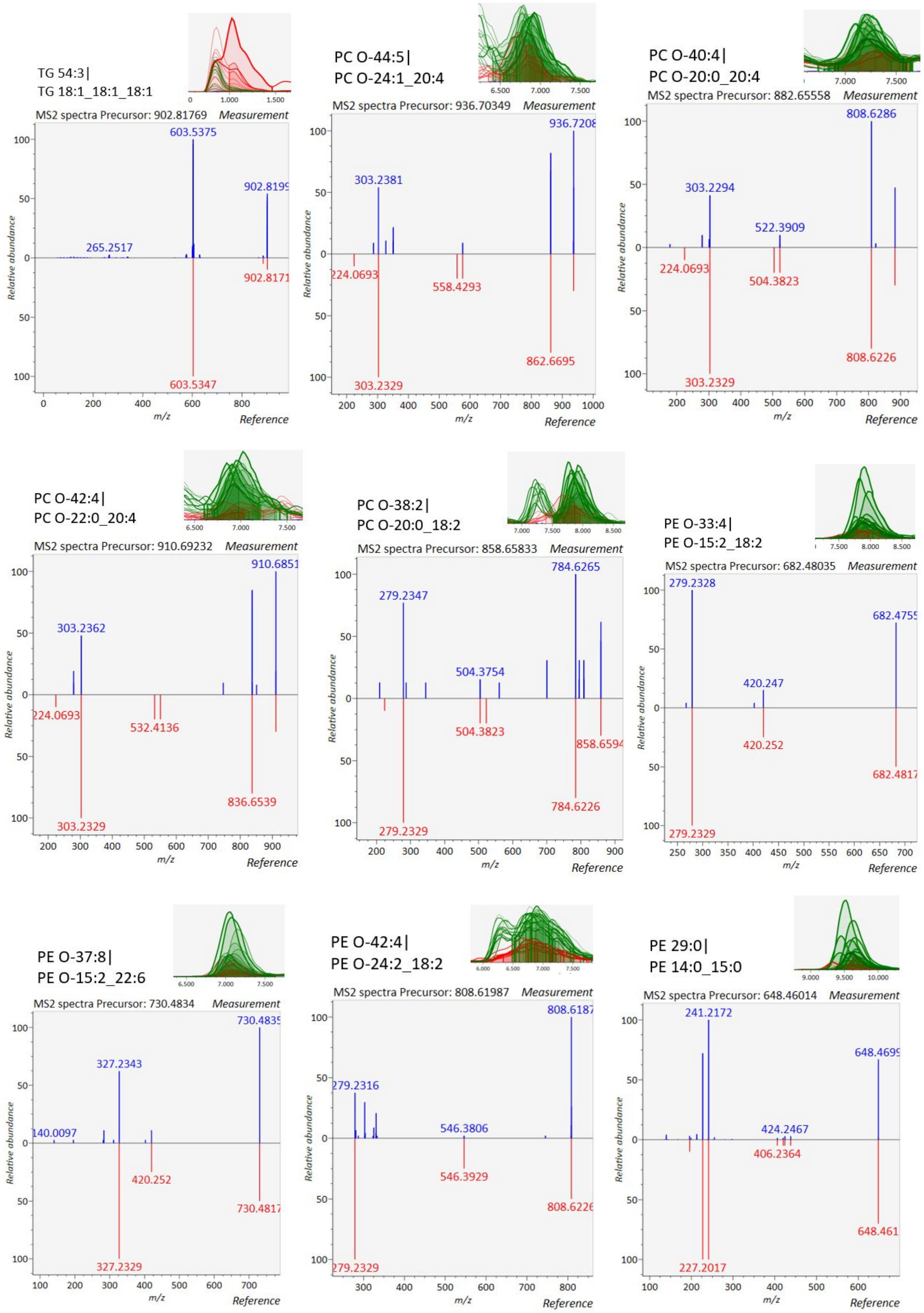
TG 54:3|
TG 18:1_18:1_18:1
MS2 spectra Precursor: 902.81769 Measurement
603.5375
902.8199
265.2517
902.8171
603.5347
Relative abundance
m/z
Reference
PC O-44:5|
PC O-24:1_20:4
MS2 spectra Precursor: 936.70349 Measurement
936.7208
303.2381
224.0693
558.4293
862.6695
303.2329
PC O-40:4|
PC O-20:0_20:4
MS2 spectra Precursor: 882.65558 Measurement
808.6286
303.2294
522.3909
224.0693
504.3823
808.6226
303.2329
PC O-42:4|
PC O-22:0_20:4
MS2 spectra Precursor: 910.69232 Measurement
910.6851
303.2362
224.0693
532.4136
836.6539
303.2329
PC O-38:2|
PC O-20:0_18:2
MS2 spectra Precursor: 858.65833 Measurement
784.6265
279.2347
504.3754
504.3823
858.6594
784.6226
279.2329
PE O-33:4|
PE O-15:2_18:2
MS2 spectra Precursor: 682.48035 Measurement
279.2328
682.4755
420.247
420.252
682.4817
279.2329
PE O-37:8|
PE O-15:2_22:6
MS2 spectra Precursor: 730.4834 Measurement
730.4835
327.2343
140.0097
420.252
730.4817
327.2329
PE O-42:4|
PE O-24:2_18:2
MS2 spectra Precursor: 808.61987 Measurement
808.6187
279.2316
546.3806
546.3929
808.6226
279.2329
PE 29:0|
PE 14:0_15:0
MS2 spectra Precursor: 648.46014 Measurement
241.2172
648.4699
424.2467
406.2364
648.461
227.2017

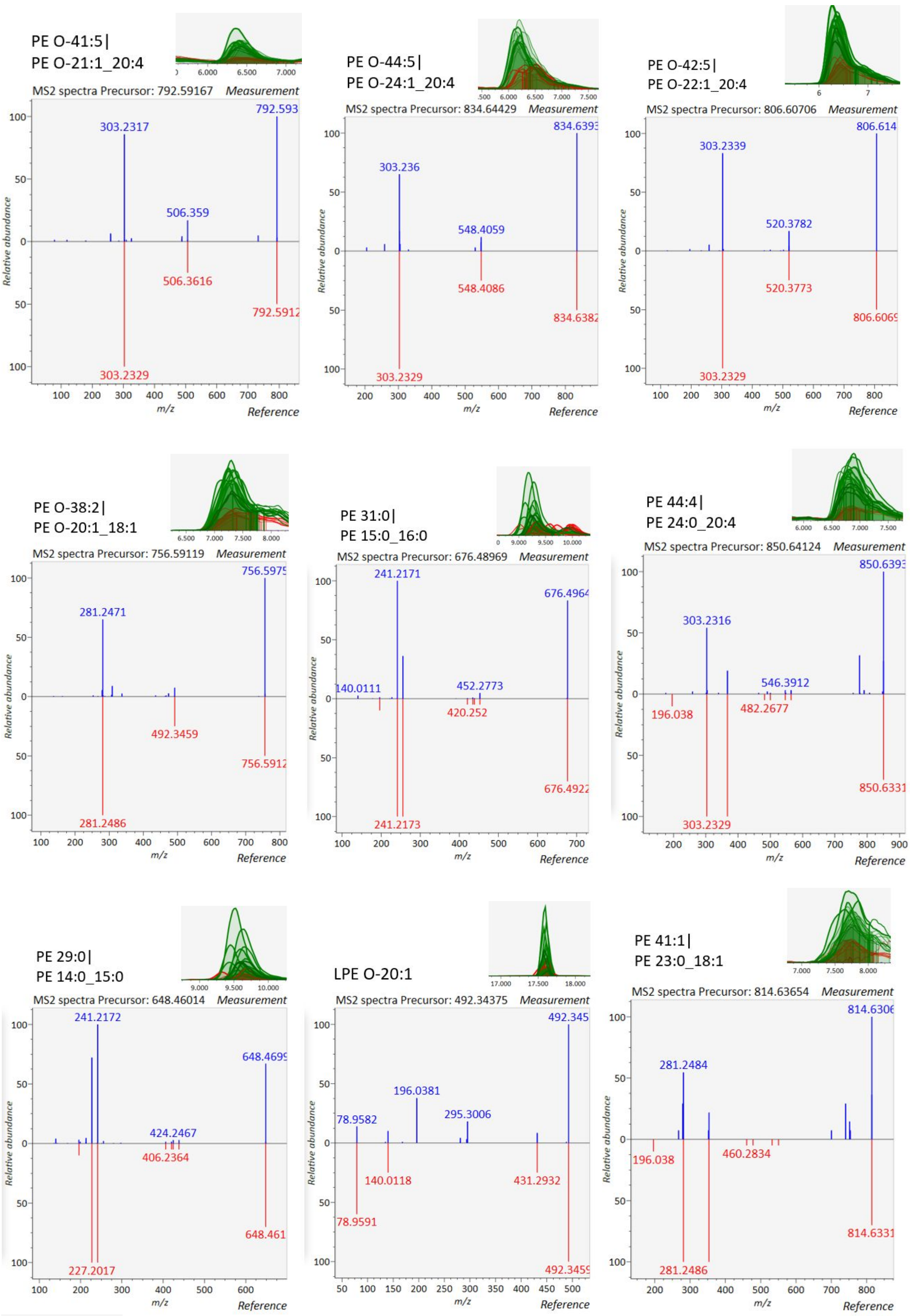
PE O-41:5|
PE O-21:1_20:4
MS2 spectra Precursor: 792.59167 Measurement
303.2317
506.359
792.593
506.3616
792.5912
303.2329
PE O-44:5|
PE O-24:1_20:4
MS2 spectra Precursor: 834.64429 Measurement
303.236
548.4059
834.6393
548.4086
834.6382
303.2329
PE O-42:5|
PE O-22:1_20:4
MS2 spectra Precursor: 806.60706 Measurement
303.2339
520.3782
806.614
520.3773
806.6069
303.2329
PE O-38:2|
PE O-20:1_18:1
MS2 spectra Precursor: 756.59119 Measurement
281.2471
756.5975
492.3459
756.5912
281.2486
PE 31:0|
PE 15:0_16:0
MS2 spectra Precursor: 676.48969 Measurement
241.2171
676.4964
140.0111
452.2773
420.252
676.4922
241.2173
PE 44:4|
PE 24:0_20:4
MS2 spectra Precursor: 850.64124 Measurement
850.6393
303.2316
546.3912
196.038
482.2677
850.6331
303.2329
PE 29:0|
PE 14:0_15:0
MS2 spectra Precursor: 648.46014 Measurement
241.2172
648.4699
424.2467
406.2364
648.461
227.2017
LPE O-20:1
MS2 spectra Precursor: 492.34375 Measurement
492.345
196.0381
295.3006
78.9582
140.0118
431.2932
78.9591
492.3459
PE 41:1|
PE 23:0_18:1
MS2 spectra Precursor: 814.63654 Measurement
814.6306
281.2484
196.038
460.2834
814.6331
281.2486
Relative abundance
m/z
Reference

LPE O-22:1

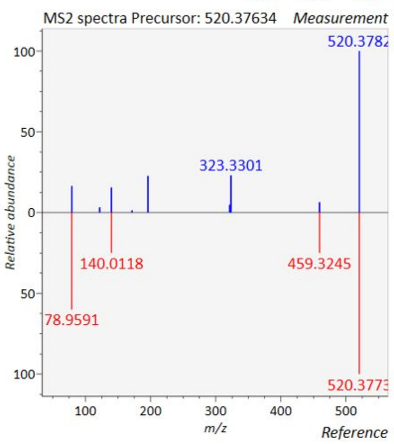

HexCer 43:0;3O|
HexCer 17:0;2O/26:0;O

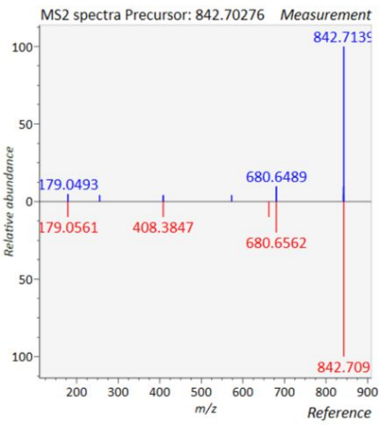

Cer 36:1;2O|
Cer 18:1;2O/18:0

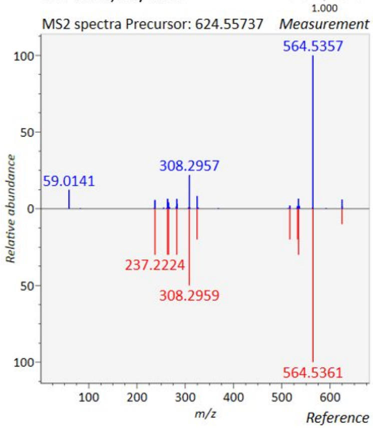

HexCer 44:0;3O|
HexCer 17:0;2O/27:0;O

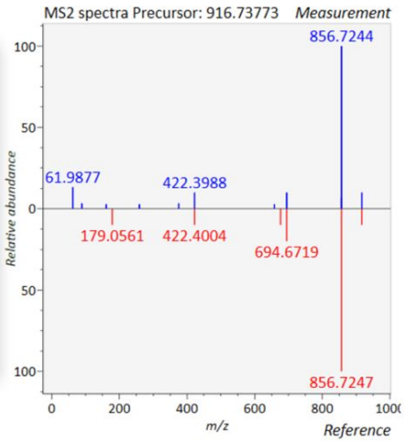

HexCer 44:1;4O|
HexCer 20:0;3O/24:1;(2OH)

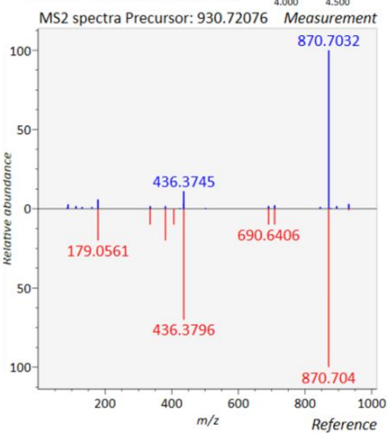

HexCer 44:1;3O|
HexCer 17:0;2O/27:1;O

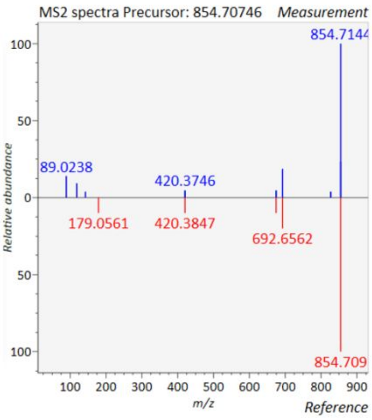

HexCer 44:0;4O|
HexCer 20:0;3O/24:0;(2OH)

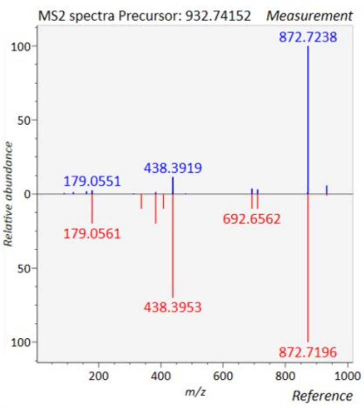

PG O-32:1|
PG O-16:1_16:0

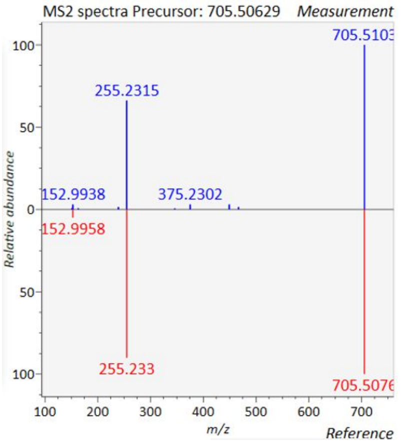

PG 30:0|
PG 14:0_16:0

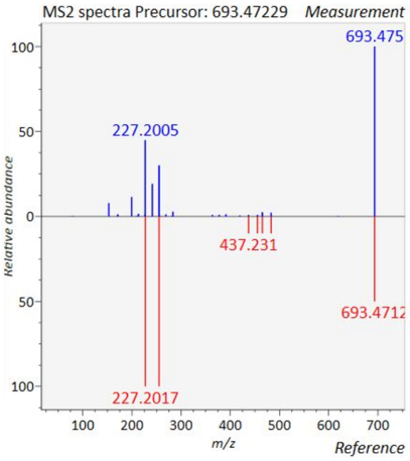

PG O-36:3|
PG O-18:2_18:1

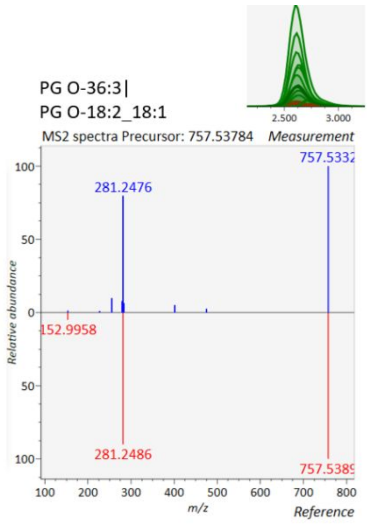

PG O-29:1|
PG O-15:1_14:0

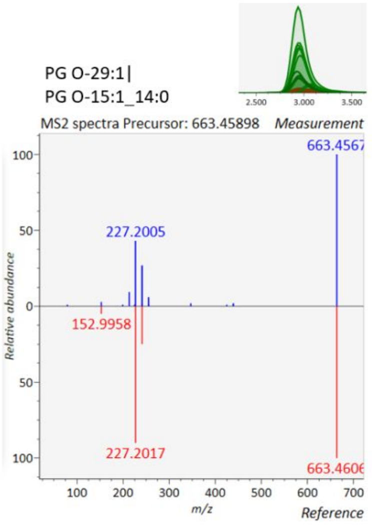

PG O-31:1|
PG O-15:1_16:0

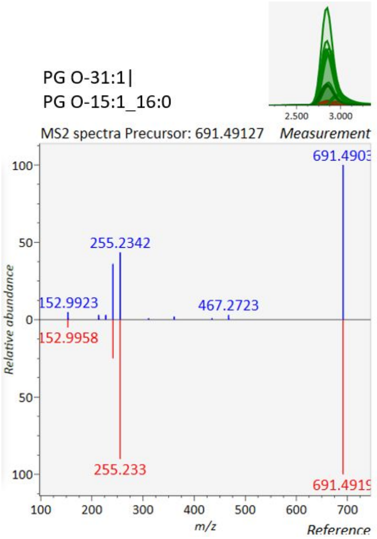

PG 31:0|
PG 15:0_16:0

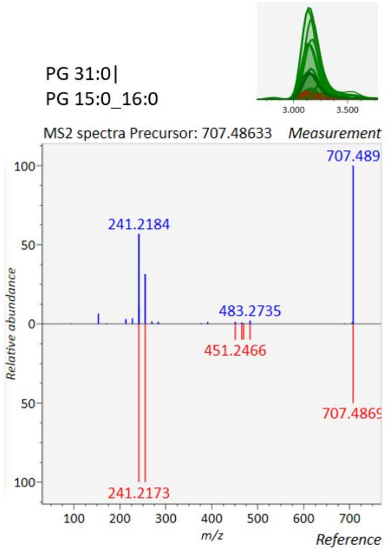

PG O-33:2|
PG O-18:2_15:0

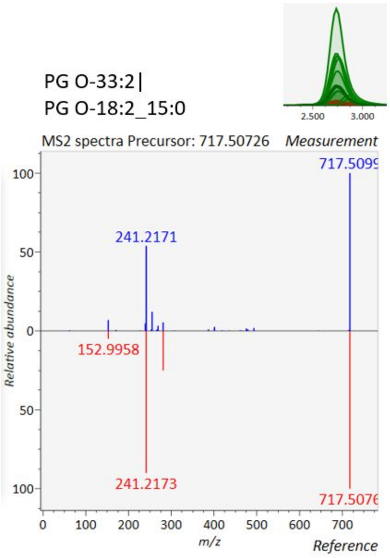

PG 36:2|
PG 18:1_18:1

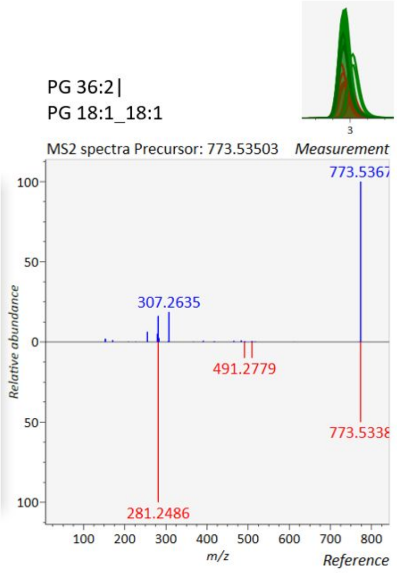

PI 34:2|
PI 16:0_18:2

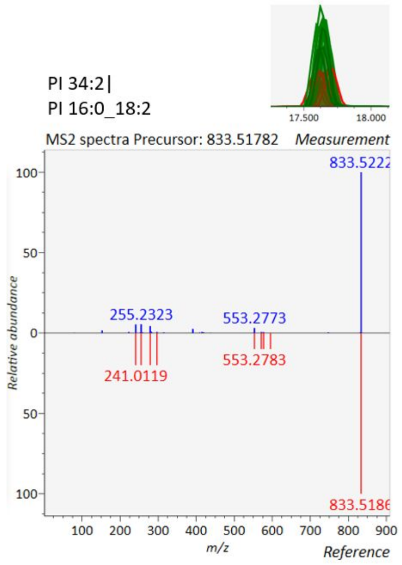

PE P-38:3|
PE P-18:0_20:3

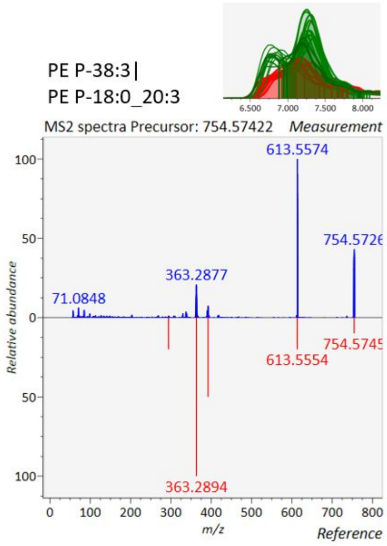

PE P-42:4|
PE P-22:0_20:4

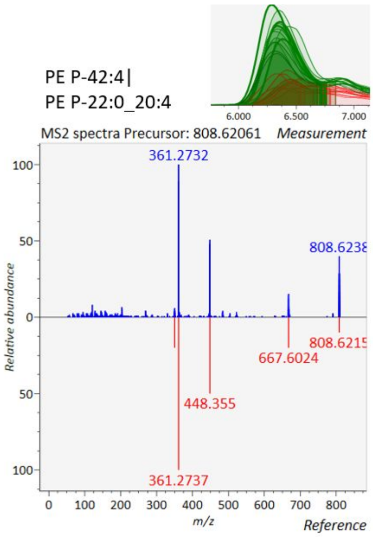

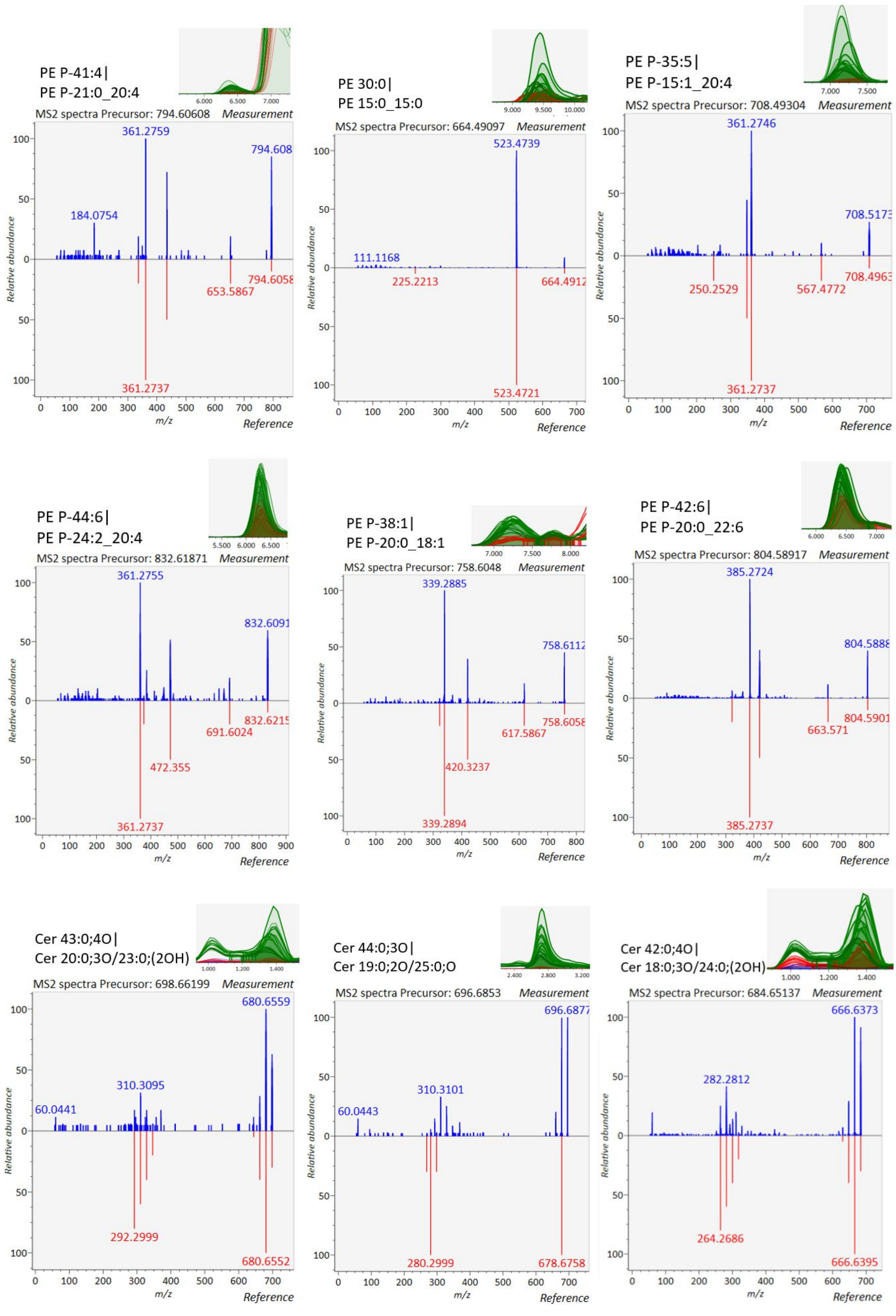
PE P-41:4|
PE P-21:0_20:4
MS2 spectra Precursor: 794.60608
PE 30:0|
PE 15:0_15:0
MS2 spectra Precursor: 664.49097
PE P-35:5|
PE P-15:1_20:4
MS2 spectra Precursor: 708.49304
PE P-44:6|
PE P-24:2_20:4
MS2 spectra Precursor: 832.61871
PE P-38:1|
PE P-20:0_18:1
MS2 spectra Precursor: 758.6048
PE P-42:6|
PE P-20:0_22:6
MS2 spectra Precursor: 804.58917
Cer 43:0;4O|
Cer 20:0;3O/23:0;(2OH)
MS2 spectra Precursor: 698.66199
Cer 44:0;3O|
Cer 19:0;2O/25:0;O
MS2 spectra Precursor: 696.6853
Cer 42:0;4O|
Cer 18:0;3O/24:0;(2OH)
MS2 spectra Precursor: 684.65137
Measurement
Reference
Relative abundance
m/z

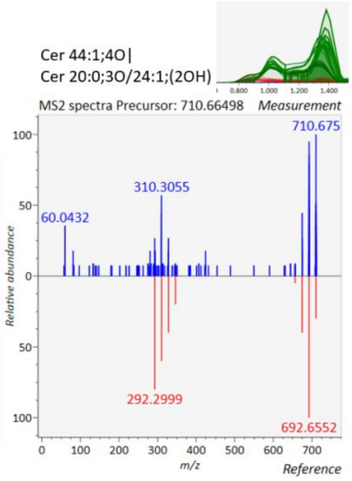
Cer 44:1;4O|
Cer 20:0;3O/24:1;(2OH)
MS2 spectra Precursor: 710.66498
Measurement
710.675
310.3055
60.0432
Relative abundance
292.2999
692.6552
m/z
Reference

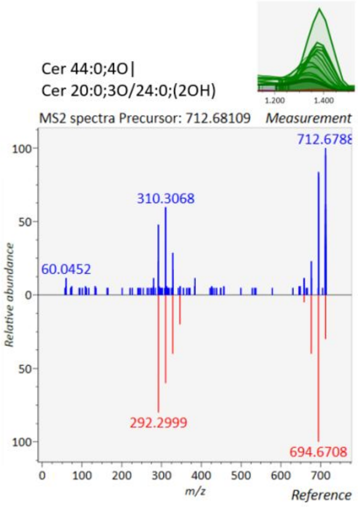
Cer 44:0;4O|
Cer 20:0;3O/24:0;(2OH)
MS2 spectra Precursor: 712.68109
Measurement
712.6788
310.3068
60.0452
Relative abundance
292.2999
694.6708
m/z
Reference
